# Synthesis and Antibacterial Evaluation of New Sulfone Derivatives Containing 2-Aroxymethyl-1,3,4-Oxadiazole/Thiadiazole Moiety

**DOI:** 10.3390/molecules22010064

**Published:** 2016-12-31

**Authors:** Shihu Su, Xia Zhou, Guoping Liao, Puying Qi, Linhong Jin

**Affiliations:** 1 State Key Laboratory Breeding Base of Green Pesticide and Agricultural Bioengineering, Key Laboratory of Green Pesticide and Agricultural Bioengineering, Ministry of Education, Research and Development Center for Fine Chemicals, Guizhou University, Guiyang 550025, China; happytt365@foxmail.com (S.S.); lgp899017@163.com (G.L.); qpuying000@163.com (P.Q.); 2 Zunyi Agricultural Products Quality and Safety Inspection and Testing Center, Room 698, Shanghai Road, Huichuan District, Zunyi 563000, Guizhou, China

**Keywords:** synthesis, 1,3,4-oxadiazole/thiadiazole sulfone, aryloxymethyl, antibacterial activity, structure-activity relationship

## Abstract

Sulfones are one of the most important classes of agricultural fungicides. To discover new lead compounds with high antibacterial activity, a series of new sulfone derivatives were designed and synthesized by introducing the aroxymethyl moiety into the scaffold of 1,3,4-oxadiazole/thiadiazole sulfones. Antibacterial activities against three phytopathogens (*Xanthomonas oryzae pv. oryzae*, *Ralstonia solanacearum*, *Xanthomonas axonopodis pv. citri*.) were assayed in vitro. As compared to the control of commercial fungicides and some reported sulfone fungicides, seven compounds **5I-1**–**5I-7** exerted remarkably higher activities with EC_50_ values ranging from 0.45–1.86 μg/mL against *X. oryzae* and 1.97–20.15 μg/mL against *R. solanacearum*. Exhilaratingly, **5I-1**, **5I-2** and **5I-4** displayed significant in vivo activity against *X. oryzae* with protective effect of 90.4%, 77.7%, and 81.1% at 200 μg/mL, respectively, much higher than that exhibited by Bismerthiazol (25.6%) and Thiadiazole-copper (32.0%). And the differential phytotoxicity of active derivatives was preliminarily checked. The results demonstrated that derivative of 2-aroxymethyl-1,3,4-oxadiazole/thiadiazole sulfone can serve as potential alternative bactericides for the management of plant bacterial diseases.

## 1. Introduction

*Xanthomonas*, a large genus of gram-negative plant-associated bacteria, causes important diseases such as bacterial blight of rice by *Xanthomonas oryzae pv. Oryzae* (*X. oryzae*) and citrus canker by *Xanthomonas axonopodis pv. citri*. (*X. axonopodis*) leading to serious quality and yield losses in the host crop [1]. *Ralstonia solanacearum* (*R. solanacearum*), a soil-borne bacterium, is responsible for bacterial wilt on more than 200 plant species including important crops such as potato, tomato, tobacco and banana [2,3]. Controlling those pathogens with bactericides such as copper based bactericides serves as the most effective way to cure plant disease [4,5]. However, the application of many currently available antibacterial agents is compromised by their low efficiency, phytotoxic effect and high accumulation in the soil [6,7]. For example, Bismerthiazol, one of the major bactericides for the control of rice bacterial leaf blight, only shows the control efficiency of 25.49% at a high dosage of 200 μg/mL [8]. Furthermore, the emergence of bactericide resistance [9,10] coupled with above drawbacks has led to the rising need for development of high-efficient bactericides to effectively control those agricultural diseases. 

**Figure 1:**
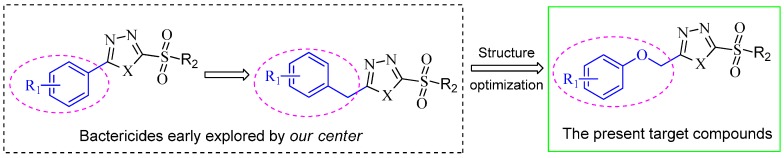
Strategic Design for the Target Compounds.

**Figure 2:**
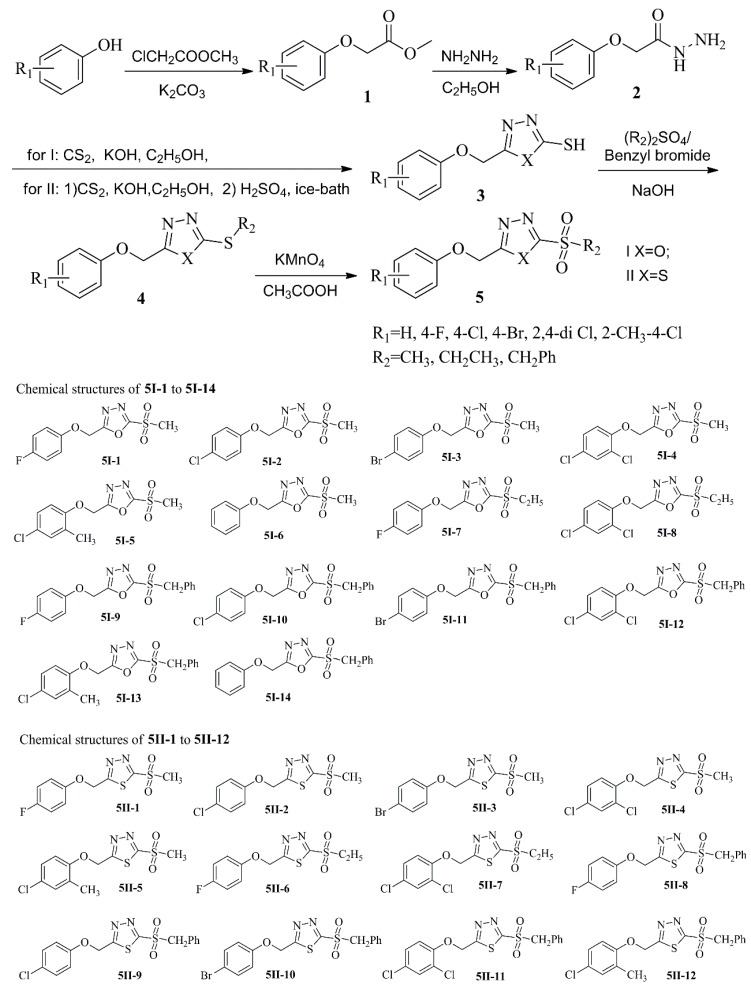
Synthetic Route to the Target Compounds **5I-1**–**5I-14** and **5II-1**–**5II-1**.

Extensive biochemical studies on sulfone derivatives bearing suitably substituted heterocyclic units have confirmed that those molecular building blocks, as a common pharmacophore model are effective against various phytopathogens in agriculture [11,12,13]. In this regard, many bactericidal agents based on heterocyclic sulfones have been patented and employed for crop protection [4,12,13,14,15,16]. For example, 1,3,4-oxadiazole/thiadiazole bearing sulfone derivatives showed potent antibacterial activities against *X. oryzae* and *R. solanacearum* [4]. We have sought new synthetic compounds related to phenyl/benzyl substituted 1,3,4-oxadiazole sulfone that have similar antibacterial action [4,15,16]. Quite interestingly, it was demonstrated that replacement of phenyl group with benzyl fragment at the 2-position of the 1,3,4-oxadiazole/thiadiazole unit (Figure 1) could significantly enhance the flexibility of the molecular backbone to combine with the receptor protein of pathogenic bacteria [14,15]. Another evidence indicated that the incorporation of an aroxymethyl group to the heterocyclic component can serve as an effective approach for enhancing antibacterial activity [17]. Intriguingly, in the course of structure optimization, the modifying of the oxime ether pharmacophore (strobilurins) with a phenoxymethyl side chain lead to the successful landing of Kresoxim-methy [18]. Fascinated by these investigations, herein we explored to build the heterocyclically substituted sulfones incorporating an aroxymethyl fragment with -O-attached to the heterocycle through linker (-CH_2_-). The design strategy and synthetic route are schematically depicted in Figure 1 and Figure 2 respectively. In our synthetic design, the aroxymethyl group involved is typical and expected to play an important role in increasing bioactivity. The inhibitory activity of target compounds on growth of various important pathogenic bacteria in vitro and in infected rice were investigated and their structure-activity relationships (SARs) were discussed. Phytotoxicity in tobacco leaves and rice seed were also determined.

## 2. Results and Discussion

### 2.1. Chemistry

Compounds **5I** and **5II** were prepared by analogous processes [17,19,20], as presented in Figure 2. Starting from phenol or substituted phenol, the key intermediate 2-aroxymethyl-1,3,4-oxadiazole-2-thiol **3** was synthesized in four steps involving esterification [17], hydrazidation, salt formation, and cyclization [19]. Subsequently, thiol 3 was converted into its corresponding thioether derivative **4** by thioetherification with dimethyl/diethyl sulfate or benzyl bromide. Finally, the target sulfonyl oxadiazole/thiadiazole **5I/II** was obtained through oxidation of the thioether **4** with potassium permanganate [20]. The structures of all the target compounds were characterized by elemental analyses, and ^1^H, ^13^C NMR and MS(ESI).

### 2.2. In Vitro Antibacterial Activity

The antibacterial activities of all the compounds were evaluated in vitro against *X. oryzae*, *R. solanacearum* and *X. axonopodis* via the turbidimeter test [21]. To make a judgment on the bactericidal potency of the synthesized compounds, three commercial antibacterials, Bismerthiazol, Kocide 3000 and Thiodiazole-copper were selected as the positive controls. As shown in Table 1, most of the products **5I** exhibited better or equivalent antibacterial activities compared to positive controls against the tested plant bacteria. Among them, compounds **5I-1**–**5I-7** appeared highly active for *X. oryzae* and *R. solanacearum* with an observed control efficacy of 100% both at 200 and 100 μg/mL. Interestingly, **5I-1**–**5I-7** exhibited inhibition rates of 65%–100% at 200 μg/mL and 48%–90% at 100 μg/mL against *X. axonopodis* which were also superior to that of positive controls. On the other hand, the present synthesis and bioactive tests were parallel conducted on **5II**. While no anticipated bioactivity was observed, except **5II-1**, **5II-2** showed better activities than positive controls against *X. oryzae* with inhibition rates of 84%–99% at 200 μg/mL. It was thus confirmed that compounds **5I** bearing 1,3,4-oxidiazole moiety were more potent in combating tested bacteria and displayed significantly higher activity as compared to compounds **5II** and positive controls.

The compounds acting better than positive controls (Table 1) were further performed for dose response with lower gradient concentration for their EC_50_ values (Table 2). Compounds **5I-1**–**5I-7** revealed outstanding activity against *X. oryzae* with EC_50_ values of 0.45, 1.44, 1.67, 0.72, 1.67, 1.86, 0.52 μg/mL respectively, which are about 1/50–1/200 fraction lower than 96.2 μg/mL displayed by Bismerthiazol. Meanwhile their EC_50_ (1.97–20.51 μg/mL) values against *R. solanacearum*, were shown to be much lower compared to the figure 45.91 μg/mL displayed by Kocide 3000. Additionally, **5I-1**–**5I-7** inhibited the growth of *X. axonopodis* with EC_50_ values ranging from 24.89 to 96.83 μg/mL. Although they were not as effective to the other two type of bacteria, but still superior to Bismerthiazol (119.5 μg/mL). More over **5I-3** is the most active against *X. axonopodis.*

In particular, **5I-1** bearing 4-F substituted phenoxymethyl, acted best for combating *X. oryzae* and *R. solanacearum* with EC_50_ values of 0.45 and 1.97 μg/mL respectively, which were far better than three commercial positive controls. Besides, the compounds **5I-2**, **5I-4**, **5I-7** with EC_50_ values of 1.44, 0.72, 0.52 μg/mL against *X. oryzae* and 13.42, 20.51, 7.75 μg/mL against *R. solanacearum*, respectively, appeared to be promising bactericides against plant bacterial diseases.

### 2.3. In Vivo Antibacterial Activity

Having established outstanding bactericidal activity of **5I-1**, **5I-2**, **5I-4** in vitro, we further explored their antibacterial potency in vivo against rice bacterial leaf blight via the leaf-cutting method [22,23] under greenhouse condition at a concentration of 200 μg/mL. Bismerthiazol and Thiodiazole-copper served as positive controls for this investigation. It was observed that all inoculated plants in 14 days exhibited blight symptoms with 100% morbidity. Plants for blank control (no bacteria inoculations) appeared absence of bacterial infection throughout the testing time. The plants for negative control (inoculated but treated with no compounds) showed severe bacterial blight symptoms with about 20.0 cm long and spreading blight lesions. While the plants treated with tested compounds were relatively healthy and had obvious shorter lesions ranged from1.9 to 4.9 cm. **5I-1** afforded a maximum of lesion reduction. It can be seen (Table 3) that, comparing to the untreated control, **5I-1**, **5I-2** and **5I-4** reduced bacterial leaf blight incidence by 90.4%, 77.7% and 81.1% respectively, which were superior to that of Bismerthiazol (25.6%) and Thiodiazole-copper (32.0%). 

### 2.4. Structure-Activity Relationship (SAR) Analyses

On the basis of the activity values shown in Table 1 and Table 2, a preliminary conclusion of the structure-activity relationship could be deduced. First, the antibacterial studies with both series compounds against tested bacteria revealed that compounds **5**I displayed significantly higher antibacterial activity than the corresponding 1,3,4-thiadiazole **5II**. Apparently the presence of heterocyclic unit 1,3,4-oxadiazole was critical to achieve significant inhibitory effect. Thus, for example, 1,3,4-oxadiazole sulfones **5I-1**–**5I-7** may serve as more potent source of antibacterial compounds compared to their corresponding 1,3,4-thiadiazole derivatives **5II**. The difference may lie in that the oxygen atom in oxadiazole is more electronegative but less bulky than sulfur atom in thiadiazole which causing different interaction with the surrounding residues. However, the strategy of oxygen–sulfur bioisosterism substitution may not necessarily lead to one way enhancement of structure bioactivity. For example, a similar trend was documented that presence of 1,3,4-oxadiazole moiety is more potential than 1,3,4-thiadiazole in imparting the antifungal activity [13]. While 1,3,4-thiadiazol-2(3*H*)-ones displayed much higher inhibition activity than the corresponding 1,3,4-oxadiazol-2(3*H*)-ones as human protoporphyrinogen oxidase inhibitors [24,25].

Further antibacterial tests on *X. oryzae* and *R. solanacearum*, have revealed that 4-substituted mono halogenated phenoxymethyl derivatives (R_1_ = 4-Cl, 4-F, 4-Br) exhibited significant antibacterial activity which appears to decrease with increase in the size of the halogen substituent. Thus the order follows the trend **5I-1**(F) > **5I-2**(Cl) > **5I-3**(Br) indicating that 4-F substituted derivative is most favorable in imparting antibacterial activity. At this point, it is in line with the previous report [13,15] that bulky groups are normally disfavored in similar structures. In addition, introduction of stronger electron-withdrawing substituents (F > Cl > Br) in the para-position of the benzene ring might assist in enhancing antibacterial activity. Specifically, when compound **5I-6**(R_1_ = H) was concerned, it is not bound to be the most potent among the 4-halogenated compounds with taking account the above two factors. 

With regard to the nature of R_2_ substituent attached to the sulfonyl unit, similar trend in terms of activity was noted with increase in size of the alkyl group:CH_3_ > CH_2_CH_3_ > CH_2_Ph, which means that methyl-sulfonyl derivatives are most active. The ethyl-sulfonyl bearing oxadiazole showed potent antibacterial activity, close to but slightly lower than the corresponding methylated one. Whereas incorporation of relatively bulky benzyl group in the sulfonyl unit led to dramatic reduction of antibacterial activity. This trend has been amply demonstrated in the observed decreasing order of activity with two different series of halogen substituents e.g., **5I-1** > **5I-7** > **5I-9**(R_1_ = F) and **5I-4** > **5I-8** > **5I-12**(R_1_ = 2,4-di Cl). A similar decreasing behavior of activity with growing size of alkyl group attached to the sulfonyl unit was also observed with **5II** series of compounds wherein oxadiazole moiety is replaced by thiadiazole skeleton. 

Finally when the results are compared with those of previously reported sulfone analogs (Table 4), where the positive controls were same and acted in identical level [4,14,15,16], compound **5I-1** provides an ideal example of being associated with exceptionally high activity against *X. oryzae* (0.45 μg/mL) and *R. solanacearum* (1.67 μg/mL). In this regard, 2-(4-fluorophenoxymethyl)-5-(methylsulfonyl)-1,3,4-oxadiazole (**5I-1**) is even more potent than the previously reported fungicides such as 2-(4-fluorophenzyl)-5-(methylsulfonyl)-1,3,4-oxadiazole [16] and 2-(4-fluorobenzyl)-5-(methylsulfonyl)-1,3,4-oxadiazole [15]. It worth to note that when the molecular weights are considered, the same mass concentration means lower molar concentration, for the present new compounds possess the higher molecular weight with an -O-, or OCH_2_ group added. Similar bioactive trend could be observed on **5I-2**(4-Cl) or **5I-4**(2,4-di Cl) with their corresponding analog bearing 2-(4-chlorophenyl) or 2-(2,4-dichlorobenyl) substituent on heterocyclic side chain. It can be further derived from earlier reported data and our present investigation (Table 4) that the nature of 2-substituent in the 1,3,4-oxadiazole ring also plays a significant role in the design of final desired structure. The antibacterial activity with 2-substituents appears to follow the trend: phenyl < benzyl < phenoxymethyl. The fact that the presence of substituted phenoxymethyl substituent at the 2-position of 1,3,4-oxadiazole moiety helps to enhance biological activity may be attributed to the improved structural flexibility offered by sp^3^-hybridized methylene (-CH_2_-), which helps to combine the receptor protein of pathogenic bacteria [15]. Furthermore, in accordance with the prediction put forward by CoMSIA 3D-QSAR, hydrogen-bond interaction offered by the oxygen atom of the alkyl aryl ether in the designated region in between phenyl and heteroalkyl group could be responsible for the enhanced bioactivity [14]. At the same time the electron withdrawing ability of the aroxymethyl group makes the compound more potent by reducing electron density on the heterocyclic system and increasing structure flexibility and stability.

### 2.5. Phytotoxic Activity

We preliminarily assessed the possible toxicity of **5I-1**, **5I-2**, **5I-4** on rice germination and tobacco leaf inoculation [27,28].

**Figure 3:**
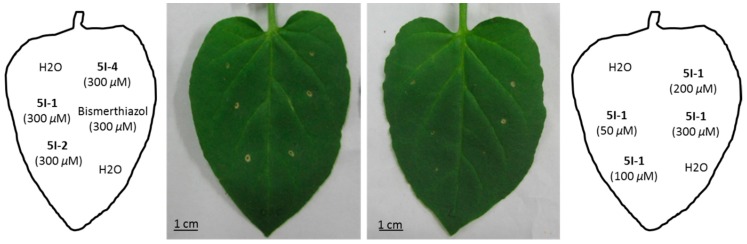
Phytotoxicity assay on tobacco plant representative examples of tobacco leaves four days after inoculation.

Initially, phytotoxic activity on tobacco leaves was assessed by infiltrating 50 μL of a 50, 100, 200 and 300 μM solution of each compound into the mesophylls of the leaves. Bismerthiazol was also included for comparison purposes, and as a reference control. After 4-day of innoculation, a brown necrotic area of around 0–0.3 cm diameter was observed (Figure 3). It appeared s no obvious difference among the inoculation spots. **5I-1**, **5I-2**, **5I-4** and Bismerthiazol appeared no or negligible phytotoxic effect on tobacco plant, with a necrosis between 0.2–0.3 cm at 300 μM concentration. And there’s no dose-response effect with a clear phytotoxicity increase from 50 to 300 μM for all treatment.

**Figure 4:**
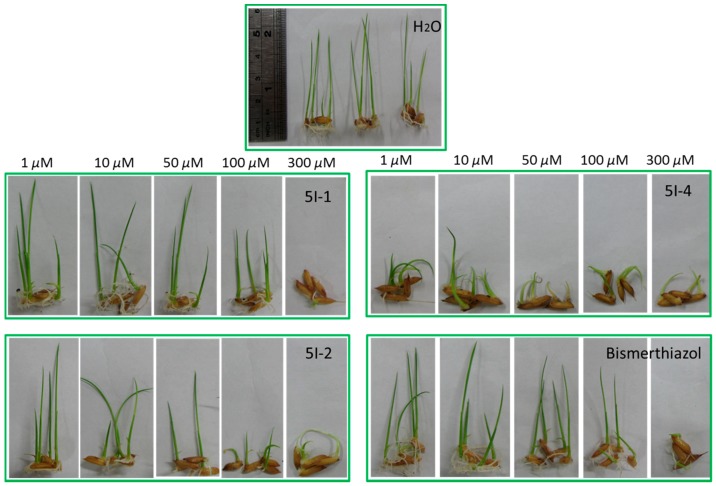
Phytotoxicity assay on rice seeds germination.

Further assays were carried out with rice germination test. Phytotoxic effects were quantified through shoot lengths of seedlings grown in the presence of decreasing concentrations of compounds (Table 5). Toxic effects were dose dependent. The high concentrations (300 μM) of all chemicals severely affected seedling development (Figure 4). **5I-1**, **5I-2** and **5I-4** were divergent on the phenotypic effects. Remarkably, **5I-4** severely inhibited both root growth and shoot development even at the lowest concentration 1 μM. **5I-2** predominantly inhibited root growth while allowing moderate shoot development. While, **5I-1**, barely effected root growth or inhibited shoot development at concentration 1–100 μM, similar to the appearance caused by Bismerthiazol. However, for the target synthetic compounds, phytotoxicity was only observed at concentrations many times higher than their bactericidal EC_50_ in terms of molar. While Bismerthiazol showed the phytotoxicity on 300 μM, which is around same level as bactericidal EC_50_ 119 μg/mL (about 333 μM).

## 3. Experimental

### 3.1. Chemicals and Instruments

All the reagents and solvents were purchased from Aladdin (Shanghai, China) and used without further purification. The progress of the reactions and purity of compounds were assessed by thin-layer chromatography (TLC) with GF_254_ silica-gel precoated sheets (Merck KGaA, Darmstadt, Germany) using hexane/ethyl acetate as eluent; the melting points of the products were determined on an XT-4 binocular microscope (Beijing Tech Instrument Co., Beijing, China). ^1^H and ^13^C NMR (solvent CDCl_3_ or DMSO-*d*_6_) spectral studies were conducted on a JEOL-ECX 500 NMR spectrometer (JEOL, Tokyo, Japan) using TMS as internal standard. Elemental analysis was performed on an Elemental Vario-III CHN analyzer (Elementar Analysensysteme GmbH, Hanau, Germany). Mass spectral studies were conducted on an Agilent LC/MSD Trap VL (Agilent, Santa Clara, CA, USA) mass spectrometer.

### 3.2. General Synthetic Procedure for the Target Compounds ***5I*** and ***5II***

The synthetic route to target compounds is shown in Figure 2 which has been designed partly based on previously reported methods [18,19,20].

#### 3.2.1. Synthesis of Substituted Phenoxyacetohydrazide (**2**) as Intermediate

To an oven-dried round bottomed flask with a stirring bar was added a solution of substituted phenol (0.01 mol, 1 eq.) and K_2_CO_3_ (1.2 eq.) in 50 mL of dry DMF. Excess ethyl chloroacetate dissolved in 10 mL of DMF was then slowly added through a dropping funnel to the above solution. The resulting mixture was stirred at 90 °C for 5–6 h. The reaction was quenched with saturated NaCl solution, then extracted with dichloromethane(DCM), washed with brine, dried over MgSO_4_ and then concentrated on a rotary evaporator affording methyl 2-phenoxyacetate **1** (80%–95% yield) which was used for the next step without further purification [17].This product 1 (0.009 mol, 1 eq.) was dissolved in 30 mL of ethanol and then placed in a round-bottomed flask equipped with a stirring bar; 80% of hydrazine hydrate (0.018 mol, 2 eq.) was slowly added to this mixture at room temperature through a dropping funnel. The resulting solution was heated up to reflux temperature and stirring was continued under this condition for another 6 h. The reaction mixture was filtered through celite, the filtrate was washed with brine, dried over MgSO_4_, and then concentrated in vacuo. The crude phenoxyacetohydrazide **2** was used directly without further purification in the next step. 

#### 3.2.2. Synthesis of the Key Intermediate 2-Thiol-5-Substituted-1,3,4-Oxadiazole/Thiadiazole (**3**)

For X=O (**3I**), to a 100 mL three necked rounded-bottomed flask was added a solution of intermediate 2 (0.009 mol, 1 eq.) and KOH (0.0108 mol, 1.2 eq.) in 50 mL absolute ethanol. This mixture was first digested and then a diluted solution carbon disulfide (0.0108 mol, 1.2 eq) in 10 mL of absolute ethanol was slowly added. The resulting mixture was stirred for 1–2 h and then refluxed at 80 °C till a homogeneous solution was obtained [19]. The alcohol was removed under reduced pressure on a rotary evaporator. The solution was washed with added brine followed by treatment with dilute hydrochloric acid solution. The pH was adjusted to 5–6 when a precipitate appeared which was subsequently filtered to afford a white solid 5-(phenoxymethyl)-1,3,4-oxadiazole-2-thiol (**3I**) (yield 60%–75%). 

For X=S (**3II**), to a 100 mL three necked rounded-bottomed flask was added a solution of intermediate 2 (0.009 mol, 1 eq.), and KOH (0.0108 mol, 1.2 eq.) in 50 mL of absolute ethanol. This mixture was first digested and then a diluted solution carbon disulfide (0.0108 mol, 1.2 eq) in 10 mL of absolute ethanol was slowly added. The resulting mixture was stirred for 5–6 h and then filtered through celite. The filtrate was concentrated in vacuo. The crude potassium 2-(2-phenoxyacetyl) hydrazinecarbodithioate (**2**–**3**) was washed with absolute ethanol alcohol and used directly in the next step for the preparation of **3II**. **2**–**3**(0.01 mol) was crushed into small powder and then slowly added to 50 mL H_2_SO_4_ (98%). An exothermic reaction ensued, the solution was stirred on an ice salt bath for 2 h, and the temperature was kept temperature below 5 °C in order to control the violence of the reaction. The progress of the reaction was monitored by TLC; the reaction was quenched by adding crushed ice equivalent to 200 mL of water. A white precipitate appeared which was filtered and then washed with water. The solid was subsequently dissolved in 10% NaOH solution and filtered again. The filtrate was then acidified with hydrochloric acid (HCl) to afford a white solid which was filtered to obtain the desired 5-(phenoxymethyl)-1,3,4-thiadiazole-2-thiol **3II**.

#### 3.2.3. Synthesis of the Intermediate 2-Thiol-5-Substituted-1,3,4-Oxadiazole/Thiadiazole (**4**)

To a solution of 2-thiol-5-substituted-1, 3, 4-oxadiazole/thiadiazole **3** (2 mmol) and sodium hydroxide (2.4 mmol, 1.2 eq.) in water (10 mL), dimethyl sulfate/diethyl sulfate (2.4 mmol, 1.2 eq.) was added drop wise with constant stirring at room temperature. The reaction was monitored for its completion by thin layer chromatography (TLC), quenched with saturated NaCl solution, then extracted with dichloromethane (3 × 20 mL). The solvent layer was washed with brine, dried over Na_2_SO_4_ and then concentrated on a rotary evaporator affording a yellow oil, 2-thiol-5-substituted-1, 3, 4-oxadiazole/ thiadiazole (**4**) (70%–80% yield). 

#### 3.2.4. Synthetic Procedure for the Target Compounds **5**

A 50 mL three-neck round-bottom flask equipped with a magnetic stirrer was charged with intermediate **4** and glacial acetic acid (15 mL). The mixture was stirred for 10 min and then 7% potassium permanganate (KMnO_4_, 5 mmol) solution was slowly added with string at room temperature [20]. After the completion of the reaction (assessed by TLC), 10% sodium bisulfite (NaHSO_3_) solution was added to deoxidize the residual KMnO_4_. The reaction mixture were subsequently filtered and the solid obtained was washed with distilled water, dried under vacuum, and recrystallized from ethanol to give compounds **5**. 

*2-(4-Fluorophenoxymethyl)-5-(methylsulfonyl)-1,3,4-oxadiazole* (**5I-1**). White acicular crystal; m.p.: 108–110 °C; ^1^H-NMR (500 MHz, CDCl_3_) 3.50 (s, 3H), 5.32 (s, 2H), 7.12–6.89 (m, 4H); ^13^C-NMR (CDCl_3_, 125 MHz) δ: 42.2, 61.6, 112.7 (d, *J* = 46.1 Hz), 116.2, 116.4 (d, *J* = 1.9 Hz), 116.5, 120.0 (d, *J* = 1.0 Hz), 155.5 (d, *J* = 468.7 Hz), 173.8, 178.9; Anal. calcd. for C_10_H_9_FN_2_O_4_S: C, 44.12; H, 3.33; N, 10.29; found: C, 43.51; H, 3.53; N, 10.33. MS (ESI) (*m*/*z*): 273.2/[M + H]^+^.

*2-((4-Chlorophenoxy)methyl)-5-(methylsulfonyl)-1,3,4-oxadiazole* (**5I-2**). White solid; m.p.: 113–114 °C; ^1^H-NMR (500 MHz, CDCl_3_) δ 7.50–7.40 (m, 2H, Ar-H), 6.96–6.87 (m, 2H, Ar-H), 5.33 (s, 2H, -CH2-), 3.50 (s, 3H, -CH_3_); ^13^C-NMR (125 MHz, CDCl_3_) δ 164.3, 163.5, 156.3, 132.8, 116.7, 115.3, 59.9, 43.0; Anal. calcd. for C_10_H_9_ClN_2_O_4_S: C, 36.23; H, 3.21; N, 8.42. Found: C, 36.05; H, 2.72; N, 8.41; MS (ESI) (*m*/*z*): 357.2/[M + Na]^+^.

*2-((4-Bromophenoxy)methyl)-5-(methylsulfonyl)-1,3,4-oxadiazole* (**5I-3**). White solid; m.p.: 97–99 °C; ^1^H-NMR (500 MHz, CDCl_3_) δ 7.30 (ddd, *J* = 8.8, 5.8, 2.9 Hz, 2H, Ar-H), 6.98–6.90 (m, 2H, Ar-H), 5.52 (s, 2H, -CH_2_-), 3.48 (s, 3H, -CH_3_); ^13^C-NMR (125 MHz, CDCl_3_) δ 172.5, 170.1, 155.6, 129.95, 129.9, 127.9, 116.2, 116.2, 65.1, 43.1; Anal. calcd. for C_10_H_9_BrN_2_O_4_S: C, 41.50; H, 3.71; N, 9.71. Found: C, 41.60; H, 3.14; N, 9.70; MS (ESI) (*m*/*z*): 311.2/[M + Na]^+^.

*2-((2,4-Dichlorophenoxy)methyl)-5-(methylsulfonyl)-1,3,4-oxadiazole* (**5I-4**). White solid; m.p.: 123–124 °C; ^1^H-NMR (500 MHz, CDCl_3_) δ 7.42 (d, *J* = 2.5 Hz, 1H, Ar-H), 7.25–7.22 (m, 1H, Ar-H), 7.05 (d, *J* = 8.8 Hz, 1H, Ar-H), 5.39 (s, 2H, -CH2-), 3.50 (s, 3H, -CH_3_); ^13^C-NMR (125 MHz, CDCl_3_) δ 163.9, 163.6, 151.8, 130.8, 128.8, 128.1, 125.2, 116.4, 61.5, 43.0; Anal. calcd. for C_10_H_8_Cl_2_N_2_O_4_S: C, 37.54; H, 2.96; N, 8.97. Found: C, 37.17; H, 2.50; N, 8.67; MS (ESI) (*m*/*z*): 323.3/[M + H]^+^.

*2-((4-Chloro-2-methylphenoxy)methyl)-5-(methylsulfonyl)-1,3,4-oxadiazole* (**5I-5**). White solid; m.p.: 96–98 °C; ^1^H-NMR (500 MHz, CDCl_3_) δ 7.17 (d, *J* = 2.5 Hz, 1H, Ar-H), 7.14 (dd, *J* = 8.6, 2.6 Hz, 1H, Ar-H), 6.86 (d, *J* = 8.6 Hz, 1H, Ar-H), 5.34 (s, 2H, -CH_2_-), 3.50 (s, 3H, -CH_3_), 2.23 (s, 3H, Ar-CH_3_); ^13^C-NMR (125 MHz, CDCl_3_) δ 164.6, 163.4, 154.1, 131.2, 129.6, 127.5, 126.7, 112.9, 60.3, 43.0, 16.1; Anal. calcd. for C_11_H_11_ClN_2_O_4_S: C, 43.91; H, 3.72; N, 9.35. Found: C, 43.64; H, 3.66; N, 9.25; MS (ESI) (*m*/*z*): 325.2/[M + Na]^+^.

*2-(Methylsulfonyl)-5-(phenoxymethyl)-1,3,4-oxadiazole* (**5I-6**). White solid; m.p.: 66–67 °C; ^1^H-NMR (500 MHz, CDCl_3_) δ: 7.37–7.29 (m, 2H, Ar-H), 7.05 (t, *J* = 7.4 Hz, 1H, Ar-H), 7.00–6.97 (m, 2H, Ar-H), 4.94 (s, 2H, -CH_2_-), 3.46 (s, 3H, -CH_3_); ^13^C-NMR (125 MHz, CDCl_3_) δ: 157.4, 154.8, 153.7, 129.8, 122.5, 115.0, 60.7, 49.0; Anal. calcd. for C_10_H_10_N_2_O_4_S: C, 47.51; H, 4.33; N, 11.33. Found: C, 47.24; H, 3.96; N, 11.02; MS (ESI) (*m*/*z*): 277.3/[M + Na]^+^.

*2-(Ethylsulfonyl)-5-((4-fluorophenoxy)methyl)-1,3,4-oxadiazole* (**5I-7**). White solid; m.p.: 106–108 °C; ^1^H-NMR (500 MHz, CDCl_3_) δ 7.12–6.90 (m, 4H), 5.32 (s, 2H), 3.58 (q, *J* = 7.4 Hz, 2H), 1.51 (t, *J* = 7.4 Hz, 3H); ^13^C-NMR (125 MHz, CDCl_3_) δ164.6, 162.7, 153.4, 116.6, 116.5, 116.4, 116.4, 115.9, 60.6, 50.2, 6.9; Anal. calcd. for C_11_H_11_FN_2_O_4_S: C, 46.15; H, 3.87; N, 9.79. Found: C, 45.35; H, 3.84; N, 9.85; MS (ESI) (*m*/*z*): 309.3/[M + Na]^+^.

*2-((2,4-Dichlorophenoxy)methyl)-5-(ethylsulfonyl)-1,3,4-oxadiazole* (**5I-8**). White solid; m.p.: 88–89 °C; ^1^H-NMR (500 MHz, DMSO-*d*_6_) δ 7.62 (d, *J* = 2.5 Hz, 1H, Ar-H), 7.39 (dt, *J* = 21.6, 5.7 Hz, 2H, Ar-H), 5.66 (s, 2H, -CH_2_-), 3.74 (q, *J* = 7.3 Hz, 2H, -CH_2_CH_3_), 1.26 (t, *J* = 7.3 Hz, 3H, -CH_3_); ^13^C-NMR (125 MHz, DMSO-*d*_6_) δ 164.6, 161.9, 151.7, 129.6, 128.2, 126.3, 122.9, 116.4, 61.1, 49.5, 6.6; Anal. calcd. for C_11_H_10_Cl_2_N_2_O_4_S: C, 39.49; H, 3.48; N, 8.65. Found: C, 39.18; H, 2.99; N, 8.31; MS (ESI) (*m*/*z*): 359.1/[M + Na]^+^.

*2-(Benzylsulfonyl)-5-((4-fluorophenoxy)methyl)-1,3,4-oxadiazole* (**5I-9**). White sold; m.p.: 94–96 °C; ^1^H-NMR (CDCl_3_, 500 MHz) δ 7.42–7.23 (m, 5H), 7.06–6.88 (m, 4H), 5.21 (s, 4H), 4.75 (s, 4H); ^13^C-NMR (CDCl_3_, 125 MHz) δ 164.7, 162.6, 158.4 (d, *J* = 240.9 Hz), 153.3, 131.2, 130.0, 129.3, 124.6, 116.5, 116.3 (t, *J* = 4.1 Hz), 62.1, 60.4; Anal. calcd. for C_16_H_13_FN_2_O_4_S: C, 55.17; H, 3.76; N, 8.04. Found: C, 55.17; H, 3.66; N, 8.29; MS (ESI) (*m*/*z*): 371.1/[M + Na]^+^.

*2-(Benzylsulfonyl)-5-((4-chlorophenoxy)methyl)-1,3,4-oxadiazole* (**5I-10**). White solid; m.p.: 126–128 °C; ^1^H-NMR (500 MHz, CDCl_3_) δ 7.38–7.26 (m, 7H, Ar-H), 6.95–6.85 (m, 2H, Ar-H), 5.23 (s, 2H, -CH_2_-), 4.75 (s, 2H, -CH_2_-); ^13^C-NMR (125 MHz, CDCl_3_) δ 164.5, 162.6, 155.7, 131.2, 130.0, 129.9, 129.3, 127.8, 124.5, 116.2, 62.1, 59.8; Anal. calcd. for C_16_H_13_ClN_2_O4_S_: C, 52.27; H, 4.13; N, 7.61. Found: C, 52.68; H, 3.59; N, 7.68; MS (ESI)(*m*/*z*): 387.2/[M + Na]^+^.

*2-(Benzylsulfonyl)-5-((4-bromophenoxy)methyl)-1,3,4-oxadiazole* (**5I-11**). White solid; m.p.: 142–143 °C; ^1^H-NMR (500 MHz, CDCl_3_) δ 7.57–7.47 (m, 2H, Ar-H), 7.42–7.23 (m, 5H, Ar-H), 7.10–7.01 (m, 2H, Ar-H), 5.55 (s, 2H, -CH_2_-), 5.20 (s, 2H, -CH2-); ^13^C-NMR (125 MHz, CDCl_3_) δ 164.4, 162.7, 156.2, 132.8, 131.2, 130.0, 129.3, 124.5, 116.7, 115.2, 62.1, 59.7; Anal. calcd. for C_16_H_13_BrN_2_O_4_S: C, 46.83; H, 3.93; N, 6.70. Found: C, 46.96; H, 3.20; N, 6.84; MS (ESI) (*m*/*z*): 433.1/[M + Na]^+^.

*2-(Benzylsulfonyl)-5-((2,4-dichlorophenoxy)methyl)-1,3,4-oxadiazole* (**5I-12**). White solid; m.p.: 121–122 °C; ^1^H-NMR (500 MHz, CDCl_3_) δ 7.68 (d, *J* = 2.6 Hz, 1H, Ar-H), 7.46 (dd, *J* = 8.9, 2.6 Hz, 1H, Ar-H), 7.40–7.29 (m, 6H, Ar-H), 5.67 (s, 2H, -CH_2_-), 5.21 (s, 2H, -CH_2_-); ^13^C-NMR (125 MHz, CDCl_3_) δ 164.6, 162.0, 151.6, 131.3, 129. 7, 129.3, 128.7, 128.2, 126.2, 125.7, 122.8, 116.0, 60.7, 60.5; Anal. calcd. for C_16_H_12_Cl_2_N_2_O_4_S: C, 48.39; H, 3.43; N, 7.32. Found: C, 48.13; H, 3.03; N, 7.02; MS (ESI) (*m*/*z*): 399.4/[M + H]^+^.

*2-(Benzylsulfonyl)-5-((4-chloro-2-methylphenoxy)methyl)-1,3,4-oxadiazole* (**5I-13**). Yellow crystal; m.p.: 144–145 °C; ^1^H-NMR (500 MHz, CDCl_3_) δ 7.39–7.35 (m, 1H, Ar-H), 7.32 (d, *J* = 1.4 Hz, 1H, Ar-H), 7.31–7.29 (m, 2H, Ar-H), 7.27 (s, 2H, Ar-H), 7.26–7.24 (m, 1H, Ar-H), 7.11 (d, *J* = 8.8 Hz, 1H, Ar-H), 5.55 (s, 2H, -CH_2_-), 5.21 (s, 2H, -CH_2_-), 2.17 (s, 3H, Ar-CH_3_); ^13^C-NMR (125 MHz, CDCl_3_) δ 174.6, 169.8, 165.8, 162.4, 154.6, 131.8, 130.9, 129.8, 129.4, 129.2, 127.1, 126.3, 125.93, 114.4, 61.0, 60.6, 16.2; Anal. calcd. for C_17_H_15_ClN_2_O_4_S: C, 53.31; H, 4.23; N, 7.26. Found: C, 53.90; H, 3.99; N, 7.39; MS (ESI) (*m*/*z*): 401.2/[M + Na]^+^.

*2-(Benzylsulfonyl)-5-(phenoxymethyl)-1,3,4-oxadiazole* (**5I-14**). White solid; m.p.: 95–96 °C; ^1^H-NMR (500 MHz, CDCl_3_) δ 7.40–7.24 (m, 7H, Ar-H), 7.06 (ddd, *J* = 11.7, 8.3, 0.9 Hz, 3H, Ar-H), 5.53 (s, 2H, -CH_2_-), 5.20 (s, 2H, -CH_2_-); ^13^C-NMR (125 MHz, CDCl_3_) δ 165.4, 161.9, 157.0, 131.3, 129.7, 129.3, 128.8, 125.8, 122.0, 114.8, 60.5, 59.5; Anal. calcd. for C_16_H_14_N_2_O_4_S: C, 58.52; H, 4.60; N, 8.66. Found: C, 58.17; H, 4.27; N, 8.48; MS (ESI) (*m*/*z*): 353.2/[M + Na]^+^.

*2-((4-Fluorophenoxy)methyl)-5-(methylsulfonyl)-1,3,4-thiadiazole* (**5II-1**). White solid; m.p.: 107–109 °C; ^1^H-NMR (500 MHz, CDCl_3_) δ 7.08–7.00 (m, 2H, Ar-H), 6.99–6.92 (m, 2H, Ar-H), 5.52 (s, 2H, -CH_2_-), 3.48 (s, 3H, -CH_3_); ^13^C-NMR (125 MHz, CDCl_3_) δ 172.9, 170.0, 159.3, 157.4, 153.1, 116.6, 116.4, 116.2, 116.2, 65.5, 43.1; Anal. calcd. for C_10_H_9_FN_2_O_3_S_2_: C, 41.64; H, 3.49; N, 9.93. Found: C, 41.66; H, 3.15; N, 9.72; MS (ESI) (*m*/*z*): 311.2/[M + Na]^+^.

*2-((4-Chlorophenoxy)methyl)-5-(methylsulfonyl)-1,3,4-thiadiazole* (**5II-2**). White solid; m.p.: 119–120 °C; ^1^H-NMR (500 MHz, CDCl_3_) δ 7.46–7.26 (m, 2H, Ar-H), 7.15 (dd, *J* = 5.8, 3.2 Hz, 2H, Ar-H), 5.73 (s, 2H, -CH_2_-), 3.66 (s, 3H, -CH3); ^13^C-NMR (125 MHz, CDCl_3_) δ 172. 7, 170.6, 156.5, 130.0, 126.3, 117.4, 64.9, 43.6; Anal. calcd. for C_10_H_9_ClN_2_O_3_S_2_: C, 39.53; H, 3.32; N, 9.20. Found: C, 39.41; H, 2.98; N, 9.19; MS (ESI) (*m*/*z*): 327.1/[M + Na]^+^.

*2-((4-Bromophenoxy)methyl)-5-(methylsulfonyl)-1,3,4-thiadiazole* (**5II-3**). Yellow crystal; m.p.: 114–115 °C; ^1^H-NMR (500 MHz, CDCl_3_) δ 7.49–7.40 (m, 2H, Ar-H), 6.94–6.85 (m, 2H, Ar-H), 5.52 (s, 2H, -CH_2_-), 3.47 (s, 3H, -CH_3_); ^13^C-NMR (125 MHz, CDCl_3_) δ 172.5, 170.1, 156.1, 132.9, 116.7, 115.2, 65.0, 43.1; Anal. calcd. for C_10_H_9_BrN_2_O_3_S_2_: C, 33.29; H, 2.99; N, 7.76. Found: C, 34.39; H, 2.60; N, 8.02; MS (ESI) (*m*/*z*): 373.1/[M + Na]^+^.

*2-((2,4-Dichlorophenoxy)methyl)-5-(methylsulfonyl)-1,3,4-thiadiazole* (**5II-4**). Yellow crystal; m.p.: 160–162 °C; ^1^H-NMR (500 MHz, CDCl_3_) δ 7.45 (d, *J* = 2.5 Hz, 1H, Ar-H), 7.25 (dd, *J* = 8.8, 2.5 Hz, 1H, Ar-H), 6.98 (d, *J* = 8.8 Hz, 1H, Ar-H), 5.57 (s, 2H, -CH_2_-), 3.49 (s, 3H, -CH_3_); ^13^C-NMR (125 MHz, CDCl_3_) δ 172.0, 170.3, 151.6, 130.8, 128.1, 124.6, 120.0, 115.1, 66.2, 43.2; Anal. calcd. for C_10_H_8_Cl_2_N_2_O_3_S_2_: C, 35.37; H, 2.81; N, 8.26. Found: C, 35.41; H, 2.38; N, 8.26; MS (ESI) (*m*/*z*): 361.1/[M + Na]^+^.

*2-((4-Chloro-2-methylphenoxy)methyl)-5-(methylsulfonyl)-1,3,4-thiadiazole* (**5II-5**). White solid; m.p.: 160–161 °C; ^1^H-NMR (500 MHz, CDCl_3_) δ 7.19 (d, *J* = 2.5 Hz, 1H, Ar-H), 7.16 (dd, *J* = 8.6, 2.6 Hz, 1H, Ar-H), 6.81 (d, *J* = 8.6 Hz, 1H, Ar-H), 5.52 (s, 2H, -CH_2_-), 3.49 (s, 3H, -CH_3_), 2.27 (s, 3H, Ar-CH_3_); ^13^C-NMR (125 MHz, CDCl_3_) δ 172.9, 170.0, 153.8, 131.3, 129.0, 127.4, 126.8, 112.3, 65.2, 43.1, 16.3; Anal. calcd. for C_11_H_11_ClN_2_O_3_S_2_: C, 39.95; H, 3.95; N, 8.57. Found: C, 41.44; H, 3.48; N, 8.79; MS (ESI) (*m*/*z*): 341.2/[M + Na]^+^.

*2-(Ethylsulfonyl)-5-((4-fluorophenoxy)methyl)-1,3,4-thiadiazole* (**5II-6**). Yellow crystal; m.p.: 89–90 °C; ^1^H-NMR (500 MHz, CDCl_3_) δ 7.04 (dd, *J* = 9.1, 8.1 Hz, 2H, Ar-H), 6.95 (dd, *J* = 9.1, 4.2 Hz, 2H), Ar-H, 5.51 (s, 2H, -CH_2_-), 3.60 (q, *J* = 7.4 Hz, 2H, -CH_2_CH_3_), 1.47 (t, *J* = 7.4 Hz, 3H, -CH3); ^13^C-NMR (125 MHz, CDCl3) δ 172.9, 169.1, 159.3, 157.4, 153.2, 116.6, 116.4, 116.2, 116.2, 65.6, 50.3, 7.2; Anal. calcd. for C11H11FN2O3S2: C, 43.19; H, 4.09; N, 9.19. Found: C, 43.70; H, 3.67; N, 9.27; MS (ESI) (*m*/*z*): 325.2/[M + Na]^+^. 

*2-((2,4-Dichlorophenoxy)methyl)-5-(ethylsulfonyl)-1,3,4-thiadiazole* (**5II-7**). White solid; m.p.: 127–128 °C; ^1^H-NMR (500 MHz, CDCl_3_) δ 7.45 (d, *J* = 2.5 Hz, 1H, Ar-H), 7.25 (dd, *J* = 8.8, 2.6 Hz, ^1^H, Ar-H), 6.97 (d, *J* = 8.8 Hz, 1H, Ar-H), 5.57 (s, 2H, -CH_2_-), 3.61 (q, *J* = 7.4 Hz, 2H, -CH_2_CH_3_), 1.49 (t, *J* = 7.4 Hz, 3H, -CH_3_); ^13^C-NMR (125 MHz, CDCl_3_) δ 172.1, 169.3, 151.6, 130.7, 128.4, 128.1, 124.6, 115.1, 66.3, 50.2, 7.2; Anal. calcd. for C_11_H_10_Cl_2_N_2_O_3_S_2_: C, 37.18; H, 3.37; N, 7.96. Found: C, 37.40; H, 2.85; N, 7.93; MS (ESI) (*m*/*z*): 375.1/[M + Na]^+^. 

*2-(Benzylsulfonyl)-5-((4-fluorophenoxy)methyl)-1,3,4-thiadiazole* (**5II-8**). White solid; m.p.: 132–134 °C; ^1^H-NMR (500 MHz, CDCl_3_) δ 7.02 (dd, *J* = 9.0, 8.1 Hz, 2H, Ar-H), 6.89 (dd, *J* = 9.2, 4.2 Hz, 2H, Ar-H), 5.46 (s, 2H, -CH_2_-), 4.79 (s, 2H, -CH_2_-); ^13^C-NMR (125 MHz, CDCl_3_) δ 173.4, 168.6, 159.3, 157.4, 153.1, 153.0, 131.3, 129.6, 129.2, 125.9, 116.6, 116.4, 116.2, 116.1, 65.5, 61.9; Anal. calcd. for C_16_H_13_FN_2_O_3_S_2_: C, 52.69; H, 3.89; N, 7.64. Found: C, 52.73; H, 3.60; N, 7.69; MS (ESI) (*m*/*z*): 387.2/[M + Na]^+^. 

*2-(Benzylsulfonyl)-5-((4-chlorophenoxy)methyl)-1,3,4-thiadiazole* (**5II-9**). White solid; m.p.: 140–141 °C; ^1^H-NMR (500 MHz, CDCl_3_) δ 7.33 (ddd, *J* = 5.6, 3.5, 1.6 Hz, 2H, Ar-H), 7.30–7.28 (m, 3H, Ar-H), 7.25 (d, *J* = 1.7 Hz, 2H, Ar-H), 6.87 (dd, *J* = 8.8, 1.8 Hz, 2H, Ar-H), 5.47 (s, 2H, -CH_2_-), 4.79 (s, 2H, -CH_2_-); ^13^C-NMR (125 MHz, CDCl_3_) δ 173.1, 168.7, 155.5, 131.3, 129.9, 129.6, 129.2, 127.8, 125.8, 116.2, 65.0, 61.9; Anal. calcd. for C_16_H_13_ClN_2_O_3_S_2_: C, 50.92; H, 3.93; N, 7.50. Found: C, 50.46; H, 3.44; N, 7.36; MS (ESI) (*m*/*z*): 403.2/[M + Na]^+^. 

*2-(Benzylsulfonyl)-5-((4-bromophenoxy)methyl)-1,3,4-thiadiazole* (**5II-10**). White solid; m.p.: 147–148 °C; ^1^H-NMR (500 MHz, CDCl_3_) δ 7.48–7.39 (m, 2H, Ar-H), 7.39–7.22 (m, 5H, Ar-H), 6.88–6.78 (m, 2H, Ar-H), 5.47 (s, 2H, -CH2-), 4.78 (s, 2H, -CH2-); ^13^C-NMR (125 MHz, CDCl3) δ 173.0, 168.7, 156.0, 132.8, 131.3, 129.6, 129.2, 125.8, 116.7, 115.1, 64.9, 61.9; Anal. Calcd for C_16_H_13_BrN_2_O_3_S_2_: C, 45.37; H, 3.47; N, 6.62. Found: C, 45.18; H, 3.08; N, 6.59; MS (ESI) (*m*/*z*): 449.1/[M + Na]^+^. 

*2-(Benzylsulfonyl)-5-((2,4-dichlorophenoxy)methyl)-1,3,4-thiadiazole* (**5II-11**). White solid; m.p.: 124–125 °C; ^1^H-NMR (500 MHz, CDCl_3_) δ 7.43–7.41 (m, 1H, Ar-H), 7.37–7.27 (m, 5H, Ar-H), 7.23 (ddd, *J* = 8.8, 2.4, 1.7 Hz, 1H, Ar-H), 6.92 (dd, *J* = 8.8, 1.2 Hz, 1H, Ar-H), 5.52 (s, 2H, -CH2-), 4.79 (s, 2H, -CH_2_-); ^13^C-NMR (125 MHz, CDCl_3_) δ 172.5, 168.9, 151.5, 131.3, 130.7, 130.0, 129.2, 128.3, 128.0, 125.8, 124.53, 115.1, 66.1, 61.9; Anal. calcd. for C_16_H_12_Cl_2_N_2_O_3_S_2_: C, 46.74; H, 3.28; N, 6.80. Found: C, 46.27; H, 2.91; N, 6.75; MS (ESI) (*m*/*z*): 437.1/[M + Na]^+^. 

*2-(Benzylsulfonyl)-5-((4-chloro-2-methylphenoxy)methyl)-1,3,4-thiadiazole* (**5II-12**). White solid; m.p.: 125–126 °C; ^1^H-NMR (500 MHz, CDCl_3_) δ 7.39–7.26 (m, 5H, Ar-H), 7.18–7.10 (m, 2H, Ar-H), 6.75 (d, *J* = 8.6 Hz, 1H, Ar-H), 5.46 (s, 2H, -CH_2_-), 4.79 (s, 2H, -CH_2_-), 2.19 (s, 3H, Ar-CH_3_); ^13^C-NMR (125 MHz, CDCl_3_) δ 173.4, 168.5, 153.7, 131.3, 131.2, 129.6, 129.2, 129.0, 127.3, 126.8, 125.87, 112.3, 65.1, 61.9, 16.2; Anal. calcd. for C_17_H_15_ClN_2_O_3_S_2_: C, 51.93; H, 4.02; N, 7.17. Found: C, 51.71; H, 3.83; N, 7.09; MS (ESI) (*m*/*z*): 417.2/[M + Na]^+^.

### 3.3. X-ray Diffraction Analysis

**Figure 5:**
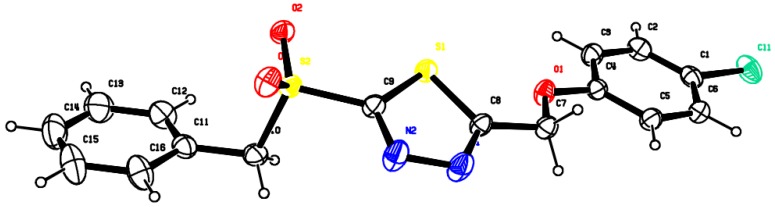
Crystal structure of compound **5II-9**.

Crystal structure of compound **5II-9** is shown in Figure 5. Colorless crystal of compound **5II-9** (0.12 mm × 0.13 mm × 0.13 mm) was mounted on a quartz fiber with protection oil. Cell dimensions and intensities were measured at 293 K on an Xcalibur (Eos, Gemini) diffractometer with graphite monochromated Cu Kα radiation (λ = 1.54184 Å). A total of 23281 reflections were measured, of which 3130 were unique (Rint = 0.0728) in the range of 4.66 < θ < 67.23° (h, −13 to 13; k, −10 to 5; l, −40 to 40), and 2142 observed reflections with I > 2σ (I) were used in the refinement on F^2^. The structure was solved by direct method with the SHELXTL-97 program. All of the non-H atoms were refined anisotropically by full-matrix least-squares to give the final R = 0.0459 and WR = 0.0459. All hydrogen atoms were computed and refined using a riding model. The atomic coordinates for **5II-9** have been deposited at the Cambridge Crystallographic Data Centre. CCDC 1008894 contains the supplementary crystallographic data for this paper.

### 3.4. Antibacterial Bioassay In Vitro

The antibacterial activity against *X. oryzae*, *R. solanacearum*, *X. axonopodis* in vitro were evaluated according to the previously reported turbidmeter test [21]. The working solution concentration was set at 200 and 100 μg/mL. Negative control(DMSO in sterile distilled water containing 0.1% Tween 20) were set up identically to test for absence of compound; Bismerthiazol (5,5′-(methylenediimino)bis-1,3,4-Thiadiazole-2(3*H*)-thione, 20% WP, China Jiangxi Heyi Chem. Ind. Co., Ltd., Pengze, China), Kocide 3000 (Copper hydroxide, 46.1%, DuPont, Wilmington, DE, USA)) and Thiodiazole-copper (2-amino-5-mercapto-1,3,4-thiadiazole-copper, 20% SC, China Zhejiang Longwan Chem. Ind. Co., Ltd., Wenzhou, China) served as positive controls. Approximately 1 mL of stock solution was added to 4 mL nontoxic liquid medium nutrient broth (NB, 3 g of beef extract, 5 g of peptone, 1 g of yeast powder, 10 g of glucose, and 1000 mL of distilled water, pH 7.0 to 7.2) in tubes. Then, approximately 40 μL NB culture media containing bacteria was added to 5 mL of NB solution containing compounds, DMSO or Kocide 3000, Thiodiazole-copper. Then, the inoculated test tubes were incubated at 30 ± 1 °C with continuous shaking at 180 rpm for 24 h. The growth of bacterial culture was monitored with a spectrophotometer by measuring the optical density at 600 nm (OD_600_). The inhibitory rate of bacterial culture growth was calculated using the following equation:

I (%) = (CK − T)/CK × 100. Where “CK” implies the value of the corrected optical density of bacterial growth on untreated NB (negative control), and “T” means the value of corrected optical density of bacterial growth on treated NB; “I” denotes the inhibition rate.

The EC_50_ values of some well performed compounds were evaluated using the above-mentioned method under 5 different concentrations (e.g., 50, 25, 12.5, 6.25, 3.12 μg/mL for *X. oryzae* and *R. solanacearum* and 200, 100, 50, 25, 12.5 μg/mL for *X. axonopodis*). The average EC_50_ was computed from at least three separate analyses for growth inhibition with Log_10_ probit analysis of SPSS 17.0.

### 3.5. Antibacterial Activity Bioassay In Vivo

The in vivo preventive activities of compounds **5I-1**, **5I-2**, **5I-4** on lesion formation caused by *X. oryzae* in rice plant were evaluated according to the following procedure under greenhouse condition [22,23]. Rice seeds were soaked with water overnight and sown in small pots. Then the rooted seedlings were transplanted when they were 20 days old in net-house. The rice plants grown for 35–40 day bearing 3–4 leaves were ready to be treated. The tested compounds were dissolved in 120 μL DMSO and diluted with 30 mL 0.1% Tween-20 to the final concentration of 200 μg/mL. For comparison, parallel test were performed on Bismerthiazol, Thiodiazole-copper (positive control), no-compound (negative control), distilled water (blank control, absence of bacterial infection and compound treatment) with three replicates of each treatment. The seedlings on the 45th day after sowing received foliar spray applications with 4 mL test compounds solution. One day later, the rice plants were clip inoculated with a bacterial suspension of *X. oryzae* culture (10^8^ CFU/mL). Development of bacterial blight lesions was scored 14 days after the inoculation. The disease severity including infection rate and disease index was determined in terms incidence of lesion length and area. The percent disease suppression in the compounds treatment was calculated as compared to disease index of the untreated control in three independent experiments. And the data were analyzed for statistical significance using the least significant difference (LSD) test. Inhibitory effect afforded by the chemical treatment from that of the untreated control was calculated with the following formula:

Disease suppression (%) = (C − T)/C × 100, where C implies disease index in untreated rice leaves; T means disease index in compound treated rice leaves.

### 3.6. Phytotoxic Bioassay

**5I-1**, **5I-2**, **5I-4** were evaluated for their phytotoxicity on rice seed germination and tobacco plant as reference described [27,28].

We initially assessed the possible toxicity on rice seed. For each treatment, a total of 4 surface-sterilized rice seeds (Fengyou Xiangzhan) were germinated in 200 μL water or compounds solution at the appropriate concentration (0, 1, 10, 50,100, and 300 μM) in each well of a 24-well plate for seven days. And 200 μL double-distilled water was implemented in the third day. Five different concentrations of test compound, in triplicate, and reared in an incubator maintained at 28 ± 1 °C with a photoperiod of 14 h light/10 h dark. Bismerthiazol was set as comparative agent. Seedling morphology was recorded and shoot length was measured. Phytotoxicity values in rice germination assays are given as minimal compound concentrations (μM) causing significant alteration of shoot length.

Tobacco (Nicotiana benthamiana) plants were grown from seed in a heated glasshouse and used between 20 and 30 days old. 50 μL chemically solution at 50, 100, 200 or 300 μM were infiltrated into the mesophylls of fully expanded tobacco leaves (previously wounded with a needle) using a micropipette. The plants were kept at standard glasshouse conditions for seven days. Up to six independent inoculations were carried out in a single leaf, and at least three independent inoculations were performed for each compound and concentration, randomly distributed in different leaves and plants. Control infiltrations with water or comparative agent Bismerthiazol at the same molar concentration were performed. The appearance of symptoms on the leaves was followed for four days after infiltration. Toxicity was measured as the lesion diameter.

## 4. Conclusions

In summary, a series of novel aroxymethyl-1,3,4-oxadiazole/thiadiazole sulfone derivatives were obtained via embedding the substituted phenoxymethyl group into scaffold of heterocyclic substituted sulfone. The antibacterial activities were studied by the turbidimeter test against pathogens *X. oryzae*, *R. solanacearum*, and *X. axonopodis*, respectively. 1,3,4-Oxadiazole sulfones **5I** are more potent as antibacterials compared to their corresponding 1,3,4-thiadiazole derivatives 5II and positive controls. Interestingly, as compared to commercial bactericides or our previous reported sulfone analogs (Table 4), compounds **5I-1**–**5I-7** possessed remarkably higher in vitro bactericidal activity against tested bacteria, which indicating that high-efficient target molecules were successfully obtained as we proposed. In vivo tests also revealed that **5I-1**, **5I-2** and **5I-4** exhibited excellent preventive effects against *X. axonopodis* with inhibitory effect of 90.4%, 77.7% and 81.1% respectively. Compared to Bismerthiazol, **5I-1**, **5I-2** exhibited no apparently phytotoxicity in tobacco leaves and rice seed germination with exception that **5I-4** highly suppressed both root and shoot development. The structure-activity relationship (SAR) analyses have suggested that aroxymethyl is found extremely useful to enhance the activity of the target structure and 1,3,4-oxadiazole sulfone derivatives bearing suitably substituted phenoxymethyl group may lead to the development of potent bactericides for crop protection.

## Figures and Tables

**Table 1:** Inhibition rate of Compounds **5I** and **5II** against Pathogenic Bacteria *X. oryzae*, *R. solanacearum* and *X. axonopodis* ^a^.

Compd.	R_1_/R_2_	*X. oryzae*		*R. solanacearum*		*X. axonopodis*	
		200 μg/mL	100 μg/mL	200 μg/mL	100 μg/mL	200 μg/mL	100 μg/mL
**5I-1**	4-F/CH_3_	100	100	100	100	80	72
**5I-2**	4-Cl/CH_3_	100	100	100	100	88	76
**5I-3**	4-Br/CH_3_	100	100	100	100	91	90
**5I-4**	2,4-diCl/CH_3_	100	100	100	100	69	48
**5I-5**	2-Me-4-Cl/CH_3_	100	100	100	100	100	54
**5I-6**	H/CH_3_	100	100	100	100	88	57
**5I-7**	4-F/CH_2_CH_3_	100	100	100	99	65	53
**5I-8**	2,4-diCl/CH_2_CH_3_	100	87	97	43	62	36
**5I-9**	4-F/CH_2_Ph	100	100	33	25	72	50
**5I-10**	4-Cl/CH_2_Ph	90	48	24	19	74	62
**5I-11**	4-Br/CH_2_Ph	56	38	18	13	11	16
**5I-12**	2,4-diCl/CH_2_Ph	98	70	33	20	26	23
**5I-13**	2-Me-4-Cl/CH_2_Ph	33	13	15	8	7	0
**5I-14**	H/CH_2_Ph	95	78	69	37	76	53
**5II-1**	4-F/CH_3_	99	92	50	49	40	36
**5II-2**	4-Cl/CH_3_	84	73	56	45	27	16
**5II-3**	4-Br/CH_3_	30	28	26	7	25	18
**5II-4**	2,4-diCl/CH_3_	16	18	18	15	23	20
**5II-5**	2-Me-4-Cl/CH_3_	42	16	9	8	25	24
**5II-6**	4-F/CH_2_CH_3_	65	55	7	12	15	18
**5II-7**	2,4-diCl/CH_2_CH_3_	20	13	0	0	32	6
**5II-8**	4-F/CH_2_Ph	24	8	30	10	24	21
**5II-9**	4-Cl/CH_2_Ph	19	14	17	18	14	23
**5II-10**	4-Br/CH_2_Ph	10	0	11	1	5	8
**5II-11**	2,4-diCl/CH_2_Ph	37	14	27	26	38	32
**5II-12**	2-Me-4-Cl/CH_2_Ph	5	0	0	0	10	33
**Bismerthiazol ^b^**		72	54	100	99	67	45
**Kocide 3000 ^b^**		69	52	100	100	39	25
**Thiodiazole-copper ^b^**		69	35	50	35	65	47

^a^ Average of three replicates; ^b^ The commercial agricultural antibacterial agents Bismerthiazol, Kocide 3000, and Thiodiazole-copper were used as positive control.

**Table 2:** Antibacterial Activity of Compounds **5I-1**–**5I-7** against *X. oryzae*, *R. solanacearu*, and *X. axonopodis* ^a^.

Compd.	*X. oryzae*			*R. solanacearum*			*X. axonopodis*		
	EC_50_ (μg/mL)	Regression Equation	r	EC_50_ (μg/mL)	Regression Equation	r	EC_50_ (μg/mL)	Regression Equation	r
**5I-1**	0.45 ± 0.06	*y* = 0.90*x* + 5.32	0.88	1.97 ± 0.23	*y* = 2.25*x* + 4.34	0.98	80.46 ± 5.38	*y* = 0.99*x* + 2.77	0.96
**5I-2**	1.44 ± 0.18	*y* = 2.73*x* + 4.57	0.93	13.42 ± 1.54	*y* = 2.79*x* + 1.86	0.92	31.35 ± 3.56	*y* = 1.95*x* + 2.09	0.98
**5I-3**	1.67 ± 0.22	*y* = 2.96*x* + 4.40	0.97	19.61 ± 0.98	*y* = 1.44*x* + 3.14	0.93	24.89 ± 2.52	*y* = 1.52*x* + 2.87	0.95
**5I-4**	0.72 ± 0.15	*y* = 2.28*x* + 5.33	0.89	20.51 ± 1.56	*y* = 2.18*x* + 2.14	0.98	49.05 ± 2.34	*y* = 1.40*x* + 2.58	0.93
**5I-5**	1.67 ± 0.16	*y* = 2.78*x* + 4.38	0.96	14.94 ± 1.27	*y* = 3.43*x* + 0.97	0.95	96.83 ± 5.78	*y* = 1.36*x* + 2.29	0.97
**5I-6**	1.86 ± 0.23	*y* = 1.59*x* + 4.57	0.90	13.55 ± 2.12	*y* = 2.62*x* + 2.04	0.91	85.02 ± 4.32	*y* = 1.06*x* + 2.96	0.96
**5I-7**	0.52 ± 0.16	*y* = 0.99*x* + 5.28	0.96	7.75 ± 1.01	*y* = 1.04*x* + 4.08	0.99	52.23 ± 2.14	*y* = 1.67*x* + 2.14	0.97
Bismerthiazol ^b^	92.61 ± 2.15	*y* = 1.50*x* + 2.05	0.98	59.69 ± 2.56	*y* = 1.21*x* + 2.84	0.98	119.5 ± 5.1	*y* = 1.50*x* + 1.82	0.98
Kocide 3000 ^b^	101.60 ± 5.12	*y* = 160*x* + 1.80	0.99	45.91 ± 6.6	*y* = 4.87*x* − 3.10	0.98	>200	/	/
Thiodiazole-copper ^b^	121.82 ± 3.59	*y* = 1.54*x* + 1.79	0.98	>200	/	/	107.04 ± 1.96	*y* = 2.15*x* + 0.94	0.98

^a^ The statistical analysis was conducted by ANOVA method at the condition of equal variances assumed (*p* > 0.05) and equal variances not assumed (*p* < 0.05); ^b^ The commercial agricultural antibacterial agents Bismerthiazol, Kocide 3000, and Thiodiazole-copper were used as positive control.

**Table 3:** In vivo Inhibitory Effect of Testing Compounds against *X. oryzae* at 200 μg/mL.

Compd.	14 Days after Spraying		
	Morbidity (%) ^c^	Disease Index	Protective Efficiency (%) ^d^
**5I-1**	100.0	15.6	90.4 ± 2.8
**5I-2**	100.0	23.8	77.7 ± 3.4
**5I-4**	100.0	17.5	81.1 ± 1.9
Bismerthiazol	100.0	60.0	25.6 ± 4.4
Thiodiazole-copper	100.0	55.6	32.0 ± 3.0
CK1 ^a^	0.0	0.0	100.0 ± 0.0
CK2 ^b^	100.0	91.1	/

^a^ CK1: blank control sample; ^b^ CK2: negative control sample; ^c^ Bacteria inoculation was successful and all inoculated plants were infected; ^d^ Statistical analysis was conducted via the ANOVA method at a condition of equal variances assumed (*p* > 0.05) and equal variances not assumed (*p* < 0.05).

**Table 4:** The Contribution of 2-Substituents in 1,3,4-Oxadiazole Ring towards Antibacterial Activity.

Plant Bacteria	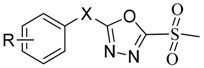 EC_50_ (μg/mL) ^a^			
	R	X = No Atom	X = CH_2_	X = OCH_2_
*X. oryzae*	F	9.89 ± 1.52 ^16^	1.07 ± 0.68 ^15^	0.45 ± 0.06 (**5I-1**)
	4-Cl	23.21 ± 0.98 ^16^	12.23 ± 1.45 ^15^	1.44 ± 0.18 (**5I-2**)
	2,4-di Cl	52.14 ± 1.05 ^16^	1.96 ± 0.99 ^15^	0.72 ± 0.15 (**5I-4**)
*R. solanacearum*	F	8.29 ± 0.56 ^14^	/ ^b^	1.97 ± 0.27 (**5I-1**)
	4-Cl	120.90 ± 2.6 ^14^	NA ^4^	13.42 ± 1.54 (**5I-2**)
	2,4-di Cl	16.55 ± 1.12 ^14^	59.9 ^26^	20.51 ± 1.56 **(5I-4**)

Note: ^a^ The EC_50_ values of the positive control for the reference compounds and present compounds were determined under the same condition and are constantly in same level; ^b^ “/”means no reported data. NA, not active. Superscript [4,14,15,16,26] are the references cited.

**Table 5:** Phytotoxicity of target bactericidal compounds on rice germination ^a^.

Comp.	Inhibition on Rice Germination Shoot Length (cm)				
	1 μM	10 μM	50 μM	100 μM	300 μM
**5I-1**	3.8 ± 0.5	3.8 ± 0.5	3.7 ± 0.6	2.8 ± 0.2	0.1 ± 0.1
**5I-2**	3.7 ± 0.6	3.2 ± 0.3	3.2 ± 0.6	1.5 ± 0.0	0.5 ± 0.1
**5I-4**	1.7 ± 0.3	1.6 ± 0.2	1.0 ± 0.1	1.1 ± 0.0	0.5 ± 0.0
Bismerthiazol ^b^	4.2 ± 0.6	3.5 ± 0.5	3.7 ± 0.4	3.2 ± 0.3	0.1 ± 0.1
H_2_O	4.6 ± 0.2				

^a^ Rice seeds germinated for seven days in H_2_O or increased concentrations of compounds. The size of rice germination shoot length (cm) is considered as a measure of phytotoxicity. The numbers indicate confidence interval of the mean. ^b^ Antibacterial agents Bismerthiazol was used as comparative agent.

## Supplementary Materials

^1^H and ^13^C NMR spectra of all the compounds are presented as Supporting Information; crystallographic data of compound **5II-9** (CCDC 1008894) for this paper could be obtained free of charge from The Cambridge Crystallographic Data Centre via www.ccdc.cam.ac.uk. The supplementary is available online at www.mdpi.com/1420-3049/22/1/64/s1.

## References

[1] Spago F.R., Ishii Mauro C.S., Oliveira A.G., Beranger J.P.O., Cely M.V.T., Stanganelli M.M., Simionato A.S., San Martin J.A.B., Andrade C.G.T.J., Mello J.C.P. (2014). *Pseudomonas aeruginosa* produces secondary metabolites that have biological activity against plant pathogenic *Xanthomonas* species. Crop Prot..

[2] Hayward A.C. (1991). Biology and epidemiology of bacterial wilt caused by *Pseudomonas solanacearum*. Annu. Rev. Phytopathol..

[3] Milling A., Babujee L., Allen C. (2011). *Ralstonia solanacearum*, extracellular polysaccharide is a specific elicitor of defense responses in wilt-resistant tomato plants. PLoS ONE.

[4] Xu W.M., Han F.F., He M., Hu D.Y., He J., Yang S., Song B.A. (2012). Inhibition of tobacco bacterial wilt with sulfone derivatives containing an 1,3,4-Oxadiazole Moiety. J. Agric. Food Chem..

[5] Denny T. (2007). Plant Pathogenic Ralstonia Species. Plant-Associated Bacteria.

[6] Imazaki I., Nakaho K. (2010). Pyruvate-amended modified SMSA medium: Imporved sensitivity for detection of *Ralstonia solanacearum*. J. Gen. Plant Pathol..

[7] Lo Cantore P., Shanmugaiah V., Iacbellis N.S. (2009). Antibacterial activity of essential oil components and their potential use in seed disinfection. J. Agric. Food Chem..

[8] Wang P.Y., Zhou L., Zhou J., Wu Z.B., Xue W., Song B.A., Yang S. (2016). Synthesis and antibacterial activity of pyridinium-tailored 2,5-substituted-1,3,4-oxadiazole thioether/sulfoxide/sulfone derivatives. Bioorg. Med. Chem. Lett..

[9] Zhu X.F., Xu Y., Peng D., Zhang Y., Huang T.T., Wang J.X. (2013). Detection and characterization of bismerthiazol-resistance of *Xanthomonas oryzae*, *pv. oryzae*. Crop Prot..

[10] Zhou X.J., Wang J., Yang Y.W., Zhao T.C., Gao B.D. (2012). Advances in tobacco bacterial wilt disease. Microbiol. China.

[11] Kleefeld G., Diehr H.J., Haas W., Dehne H.W., Brandes W. (1992). Fungicidal Agents Based on Heterocyclic Substituted Sulfones. U.S. Patent.

[12] Xu W.M., He J., He M., Han F.F., Chen X.H., Pan Z.X., Wang J., Tong M.G. (2011). Synthesis and antifungal activity of novel sulfone derivatives containing 1,3,4-oxadiazole moieties. Molecules.

[13] Xu W.M., Yang S., Bhadury P., He J., He M., Gao L.L., Hu D.Y., Song B.A. (2011). Synthesis and bioactivity of novel sulfone derivatives containing 2,4-dichlorophenyl substituted 1,3,4-oxadiazole/thiadiazole moiety as chitinase inhibitors. Pestic. Biochem. Phys..

[14] Li P., Yin J., Xu W.M., Wu J., He M., Hu D.Y., Yang S., Song B.A. (2013). Synthesis, antibacterial activities, and 3D-QSAR of sulfone derivatives containing 1,3,4-oxadiazole moiety. Chem. Biol. Drug Des..

[15] Li P., Shi L., Yang X., Yang L., Chen X.W., Wu F., Shi Q.C., Xu W.M., He M., Hu D.Y. (2014). Design, synthesis, and antibacterial activity against rice bacterial leaf blight and leaf streak of 2,5-substituted-1,3,4-oxadiazole/thiadiazole sulfone derivative. Bioorg. Med. Chem. Lett..

[16] Li S., Li P., Wang W.L., Gao M.N., Wu Z.X., Song X.P., Hu D.Y. (2015). Antibacterial activity and mechanism of action of sulfone derivatives containing 1,3,4-oxadiazole moieties on rice bacterial leaf blight. Molecules.

[17] Wei T.B., Chen J., Xu R., Zhang Y.M. (2009). Synthesis, crystal structure and biological activities of 5-(2-aryloxymethylbenzimidazole-1-carbadehyde)-1,3,4-oxadiazole-2-thine. Chin. J. Org. Chem..

[18] Sauter H., Steglich W., Anke T. (1999). Strobilurins: Evolution of a New Class of Active Substances. Angew. Chem. Int. Ed..

[19] Chen C.J., Song B.A., Yang S., Xu G.F., Bhadury P.S., Jin L.H., Hu D.Y., Li Q.Z., Liu F., Xue W. (2007). Synthesis and antifungal activities of 5-(3,4,5-trimethoxyphenyl)-2-sulfonyl-1,3,4-thiazole and 5-(3,4,5-trimethoxyphenyl)-2-sulfonyl-1,3,4-oxadiazole derivatives. Bioorg. Med. Chem..

[20] Chen Q., Zhu X.L., Jiang L.L., Liu Z.M., Yang G.F. (2008). Synthesis, antifungal activity and comfa analysis of novel 1,2,4-triazolo[1,5-a]pyrimidine derivatives. Eur. J. Med. Chem..

[21] Dalgaard P., Ross T., Kamperman L., Neumeryer K., McMeekin T.A. (1994). Estimation of bacterial growth rates from turbidimetric and viable count data. Int. J. Food Microbiol..

[22] Schaad N.W., Wang Z.K., Di M., Mcbeath J., Peterson G.L., Bonde M.R. (1996). An improved infiltration technique to test the pathogenicity of *Xanthomonas oryzae pv. oryzae* in rice seedlings. Seed Sci. Technol..

[23] Velusamy P., Immanuel J.E., Gnanamanickam S.S., Thomashow L. (2006). Biological control of rice bacterial blight by plant-associated bacteria producing 2,4-diacetylphloroglucinol. Can. J. Microbiol..

[24] Jiang L.L., Tan Y., Zhu X.L., Wang Z.F., Zuo Y., Chen Q., Xi Z., Yang G.F. (2010). Design, synthesis, and 3D-QSAR analysis of novel 1,3,4-oxadiazol-2(3*H*)-ones as protoporphyrinogen oxidase inhibitors. J. Agric. Food Chem..

[25] Zuo Y., Yang S.G., Jiang L.L., Hao G.F., Wang Z.F., Wu Q.Y., Xi Z., Yang G.F. (2012). Quantitative structure–activity relationships of 1,3,4-thiadiazol-2(3*H*)-ones and 1,3,4-oxadiazol-2(3*H*)-ones as human protoporphyrinogen oxidase inhibitors. Bioorg. Med. Chem..

[26] Xu W.M., Song B.A., Yang S., Hu D.Y., Zeng S., He M., Li P., Yin J. Antiacterial Activity of Sulfone Derivatives Containing 1,3,4-Oxadiazole Moiety. Proceedings of the Collection of the 10th National Pesticide Research and Development Conference.

[27] Vilà S., Badosa E., Montesinos E., Planas M., Feliu L. (2016). Synthetic cyclolipopeptides selective against microbial, plant and animal cell targets by incorporation of d-amino acids or histidine. PLoS ONE.

[28] Nadal A., Montero M., Company N., Badosa E., Messeguer J., Montesinos L. (2012). Constitutive expression of transgenes encoding derivatives of the synthetic antimicrobial peptide bp100: Impact on rice host plant fitness. BMC Plant Biol..

