# Supplementary material for: Synthesis and Antibacterial Evaluation of New Sulfone Derivatives Containing 2-Aroxymethyl-1,3,4-Oxadiazole/Thiadiazole Moiety
Source: Molecules. 2016 Dec 31;22(1):64. doi: 10.3390/molecules22010064 (PMC6155626; doi:10.3390/molecules22010064)
Supplement: Supplementary file 1 [file molecules-22-00064-s001.pdf]

Supplementary Materials: Synthesis and Antibacterial Evaluation of New Sulfone Derivatives Containing 2-Aroxymethyl-1,3,4-Oxadiazole/Thiadiazole Moiety

Shihu Su, Xia Zhou, Guoping Liao, Puying Qi and Linhong Jin

^{1}H and ^{13}C NMR spectra of target compound **5**.

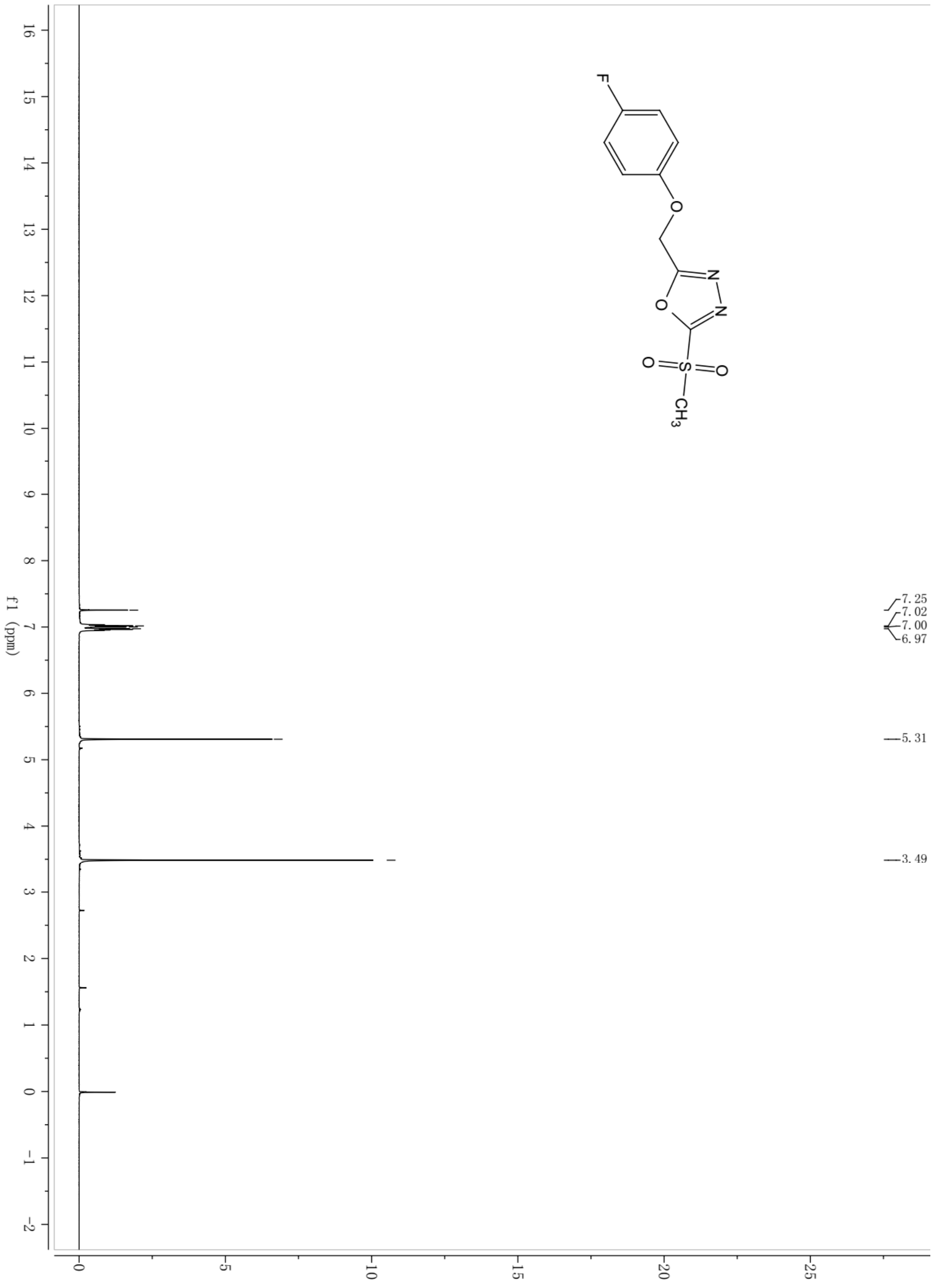

Figure S1. ^{1}H NMR spectrum of compound **5I-1**.

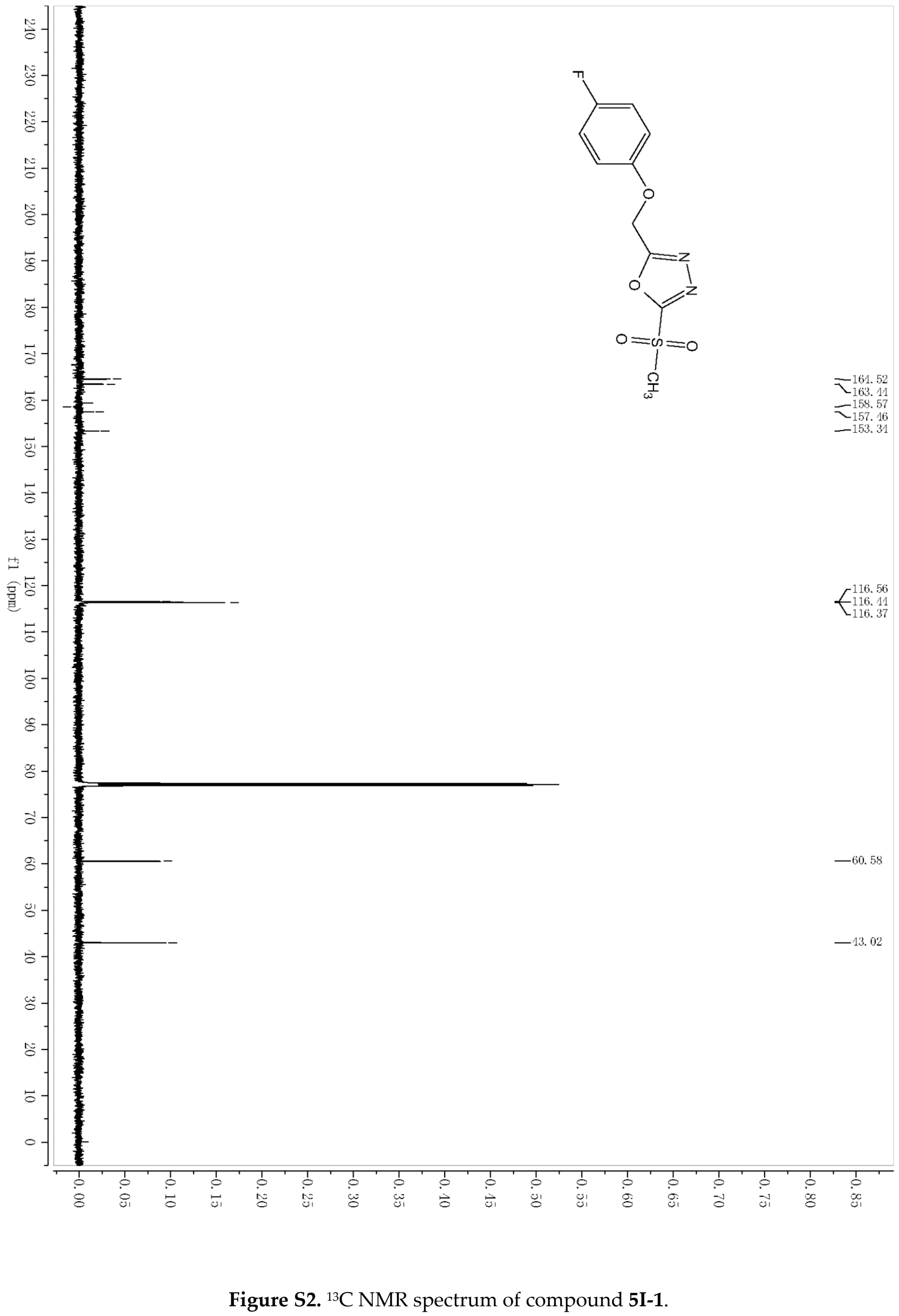

Figure S2. ^{13}C NMR spectrum of compound **5I-1**.

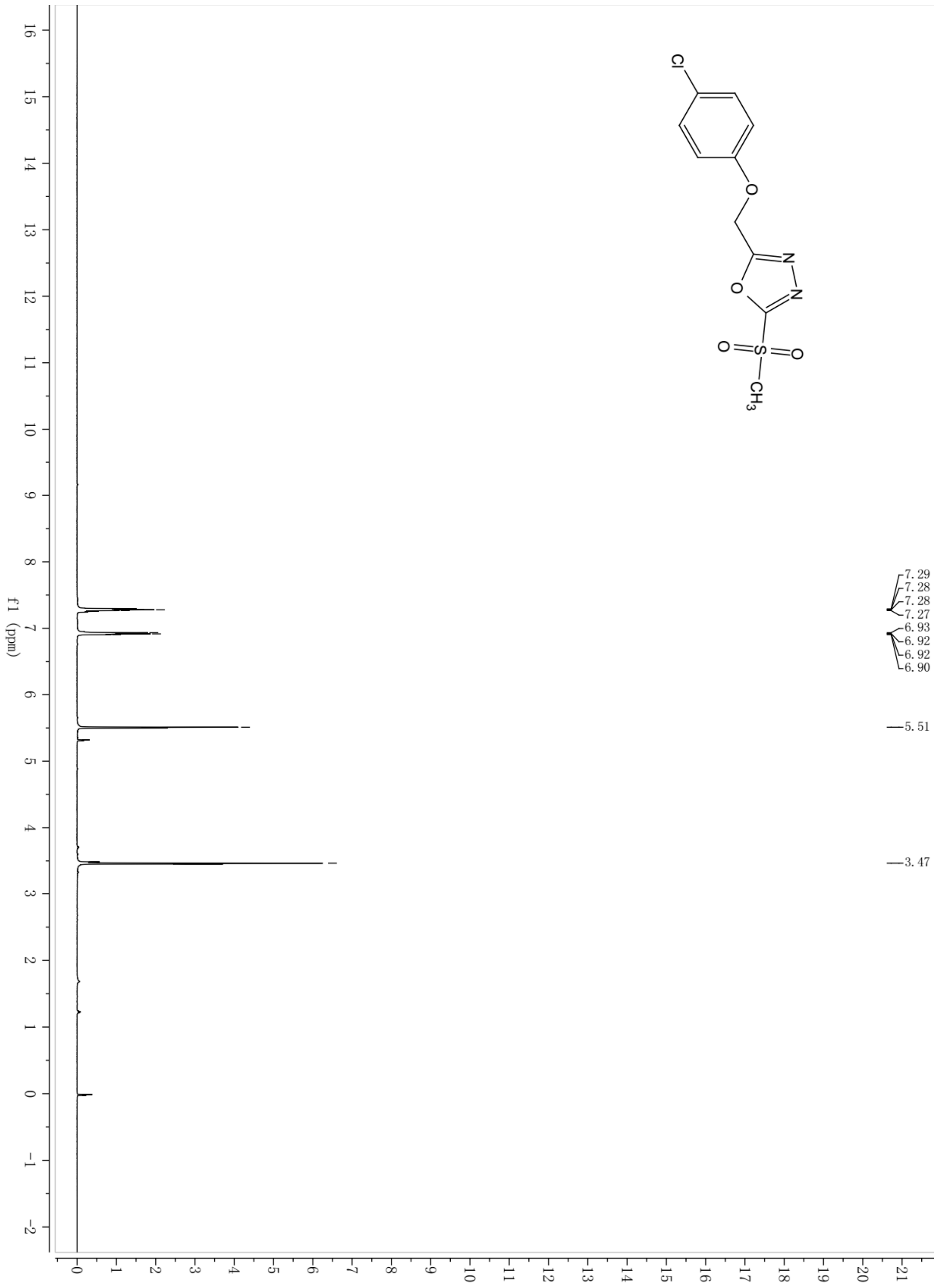

Figure S3. ^{1}H NMR spectrum of compound **5I-2**.

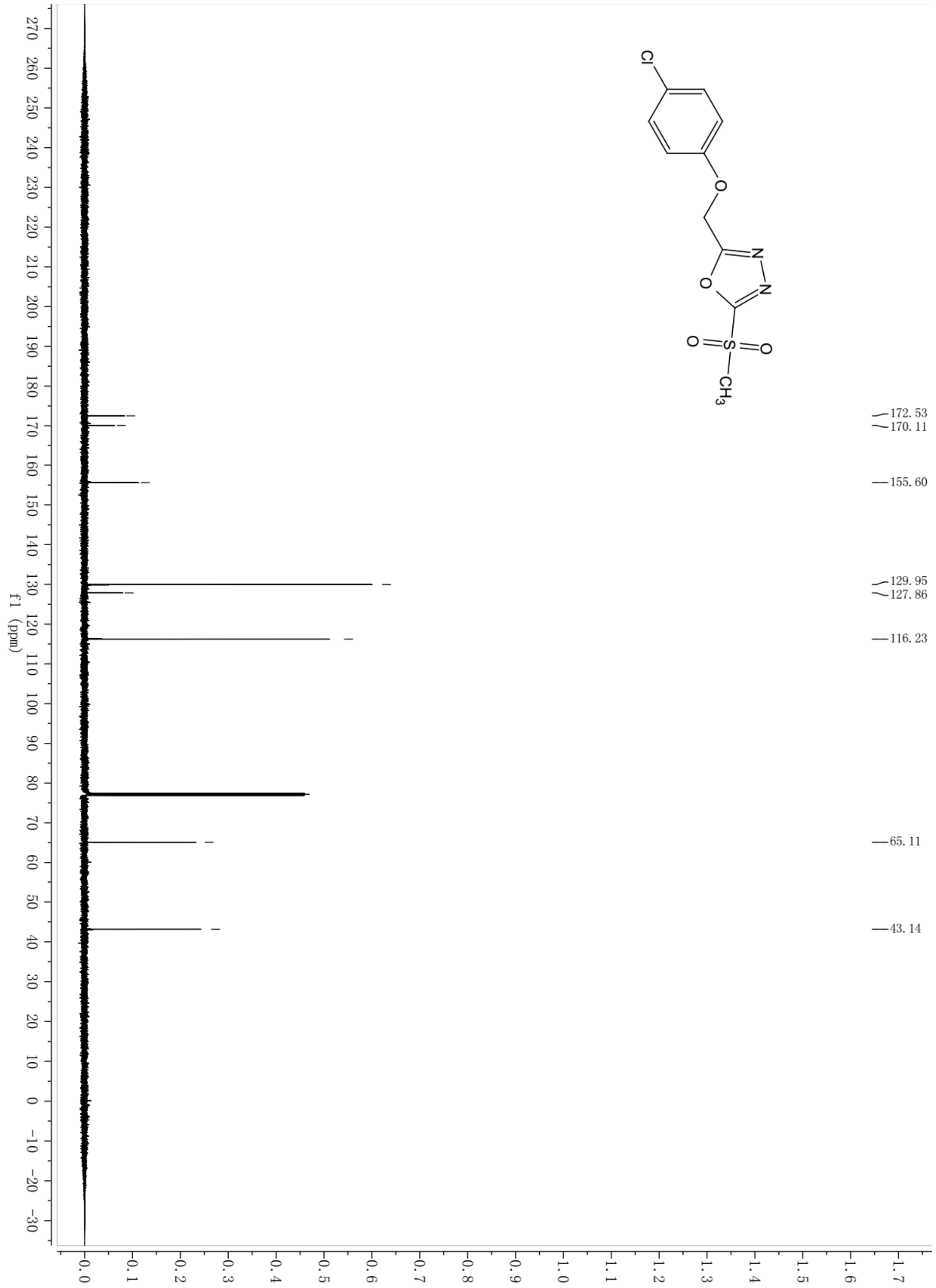

Figure S4. ^{13}C NMR spectrum of compound **5I-2**.

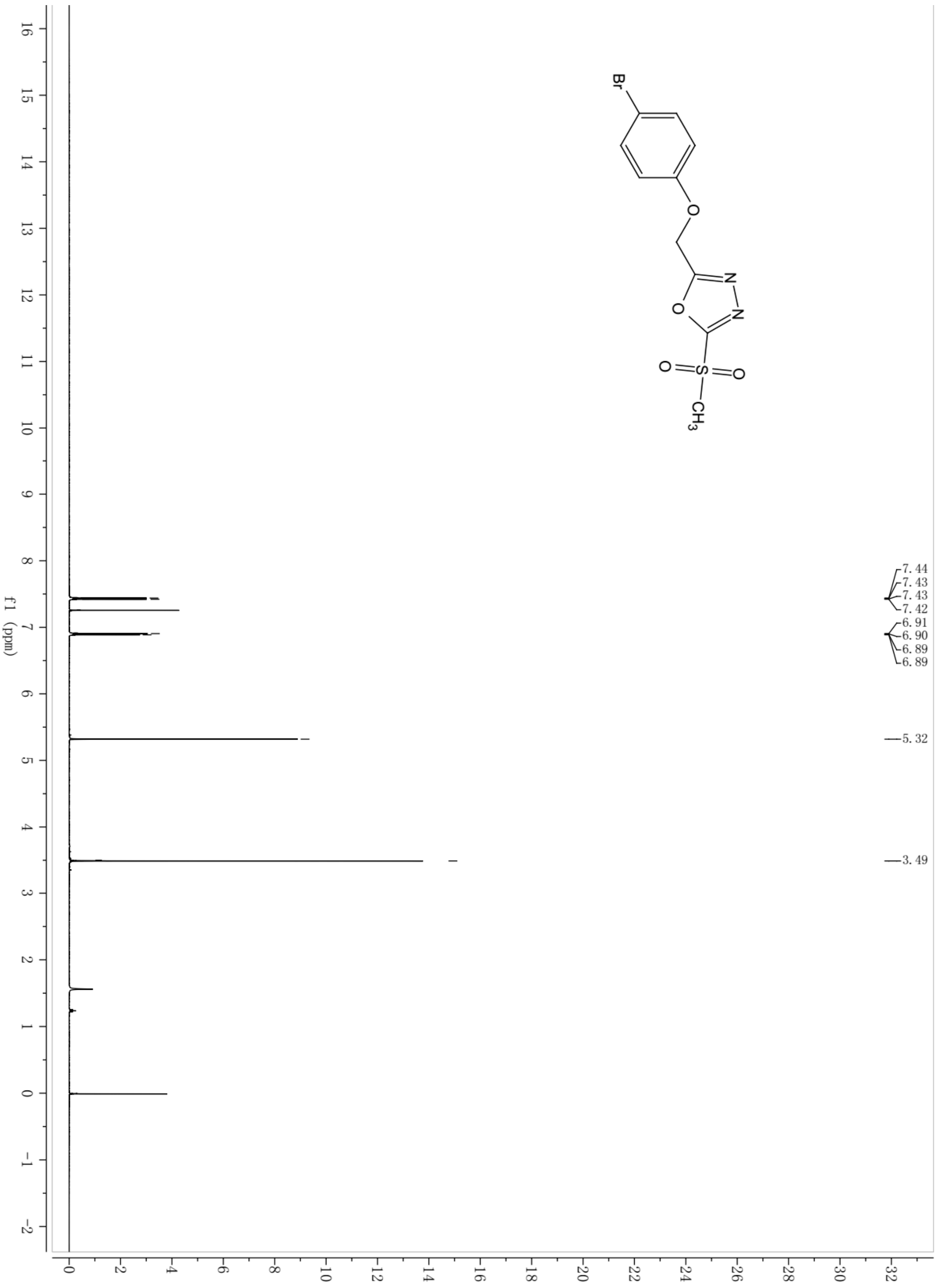

Figure S5. ^{1}H NMR spectrum of compound **5I-3**.

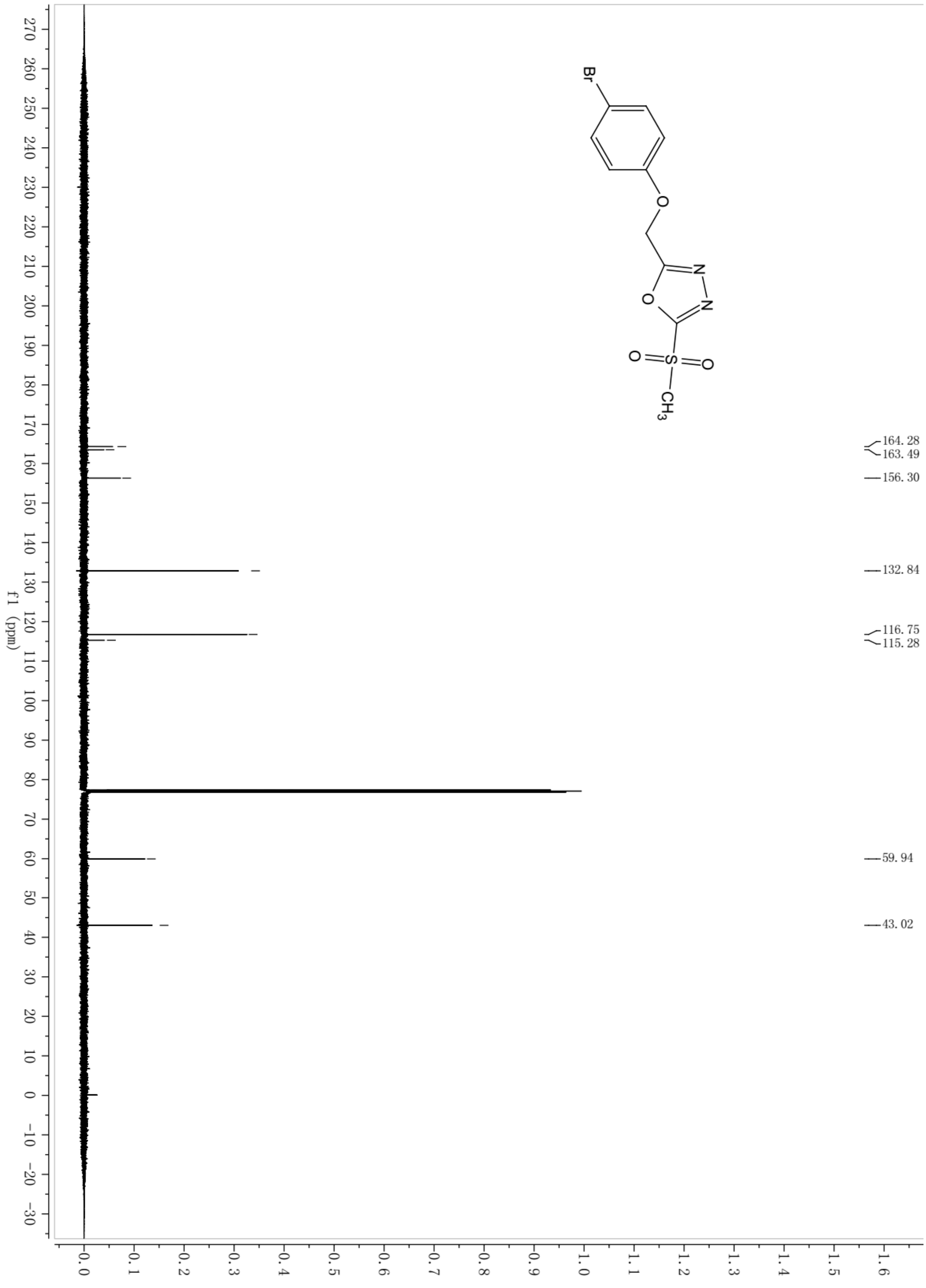

Figure S6. ^{13}C NMR spectrum of compound **5I-3**.

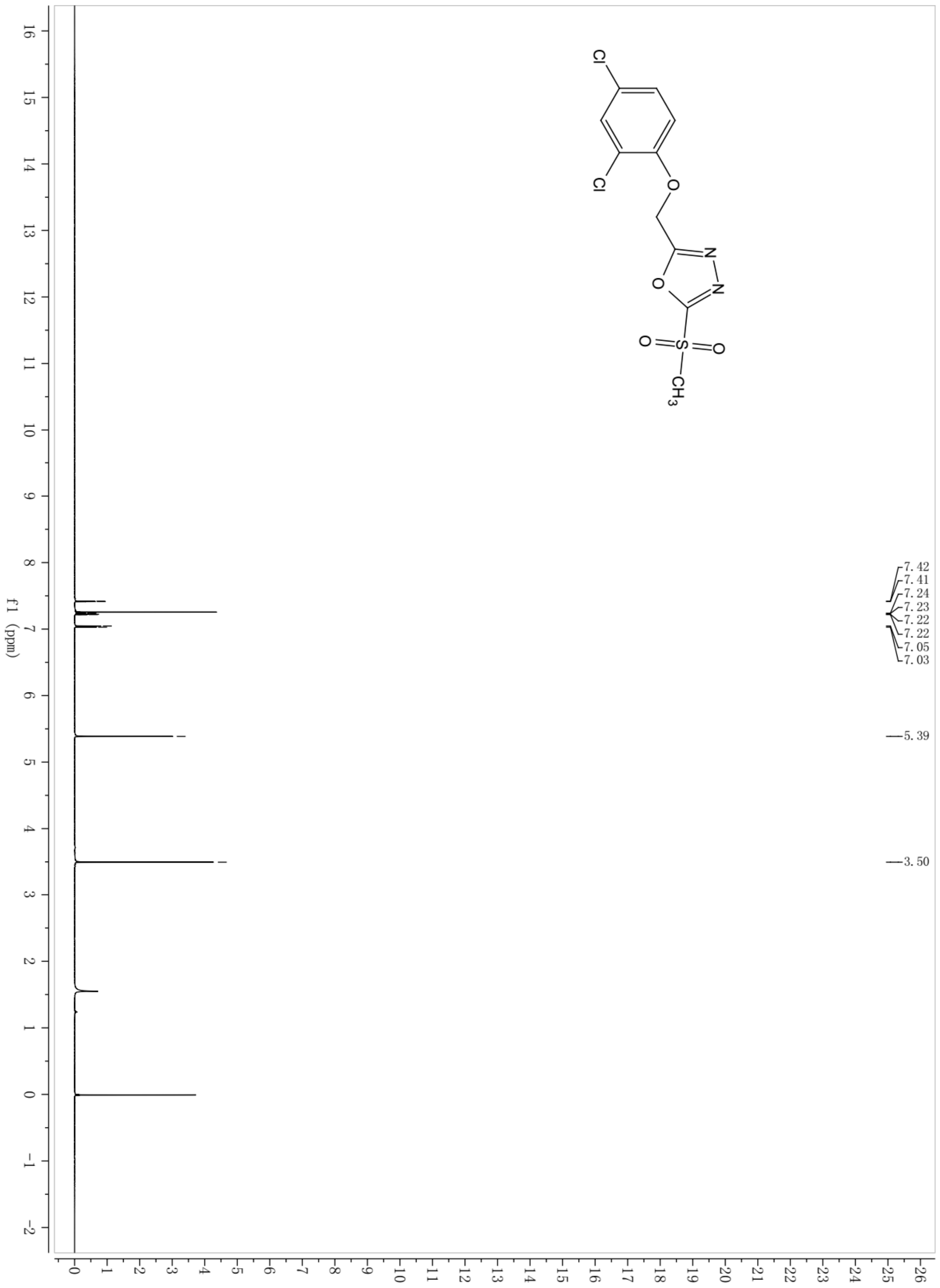

Figure S7. ^{1}H NMR spectrum of compound **5I-4**.

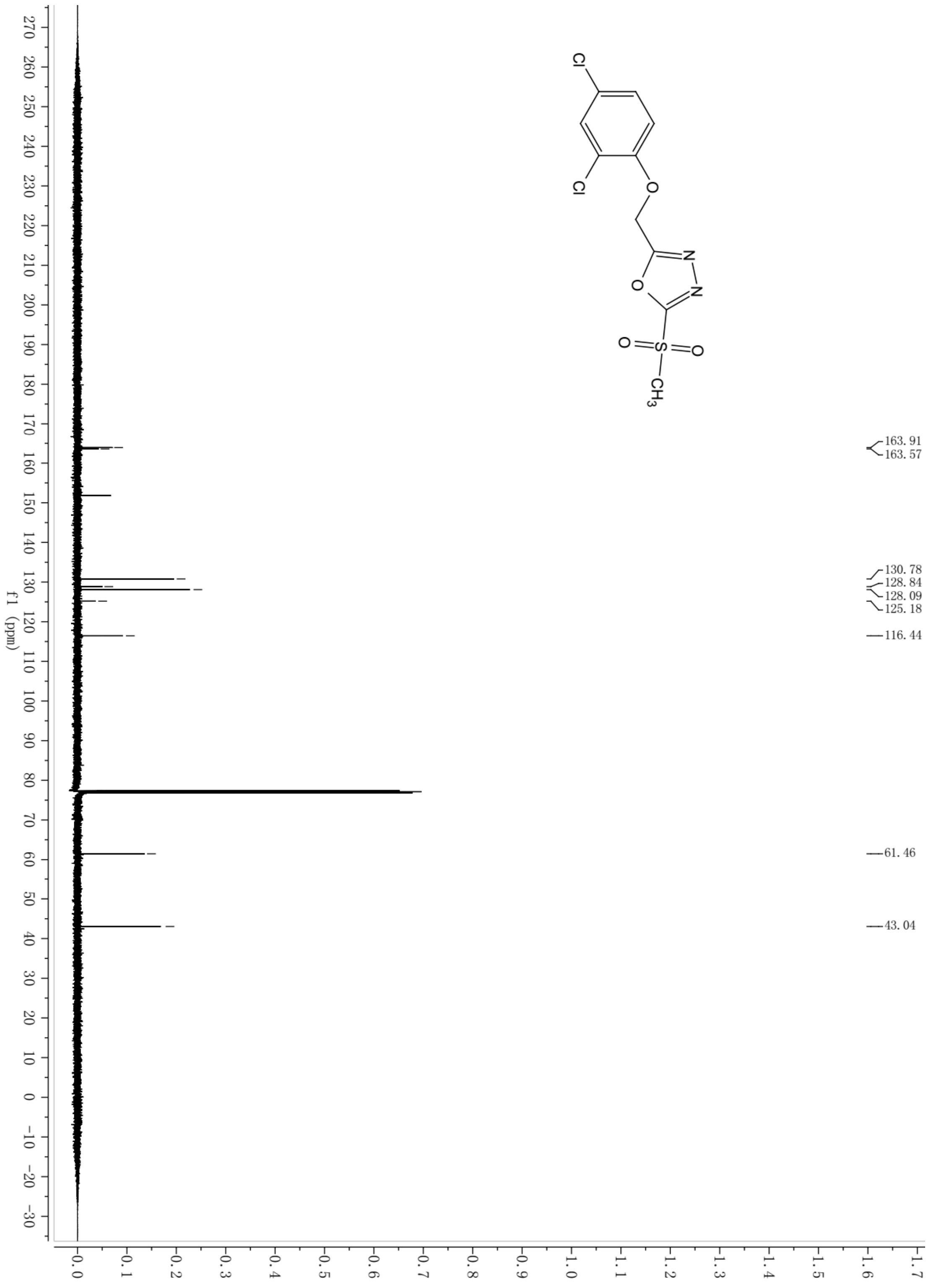

Figure S8. ^{13}C NMR spectrum of compound **5I-4**.

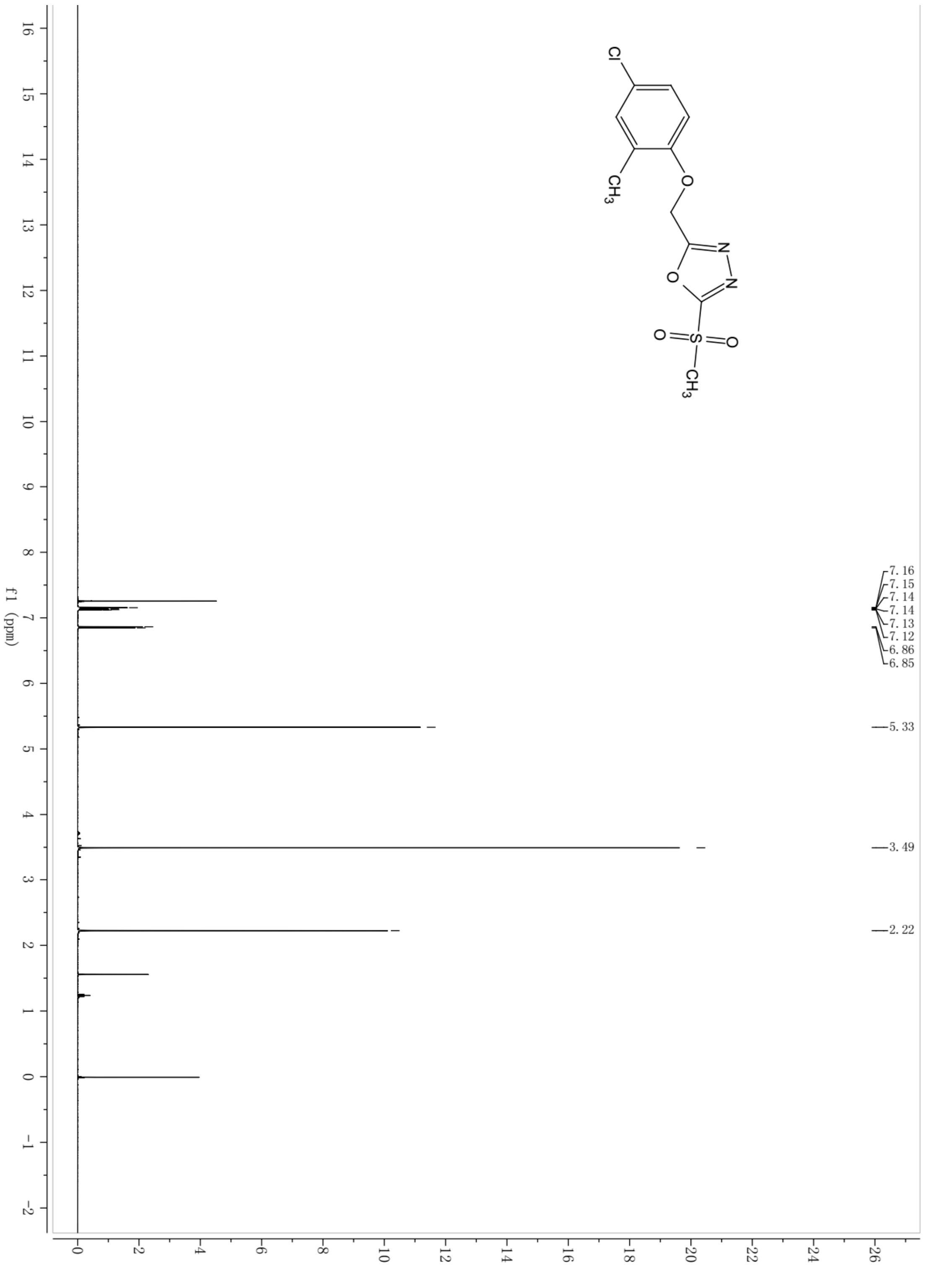

Figure S9. ^{1}H NMR spectrum of compound **5I-5**.

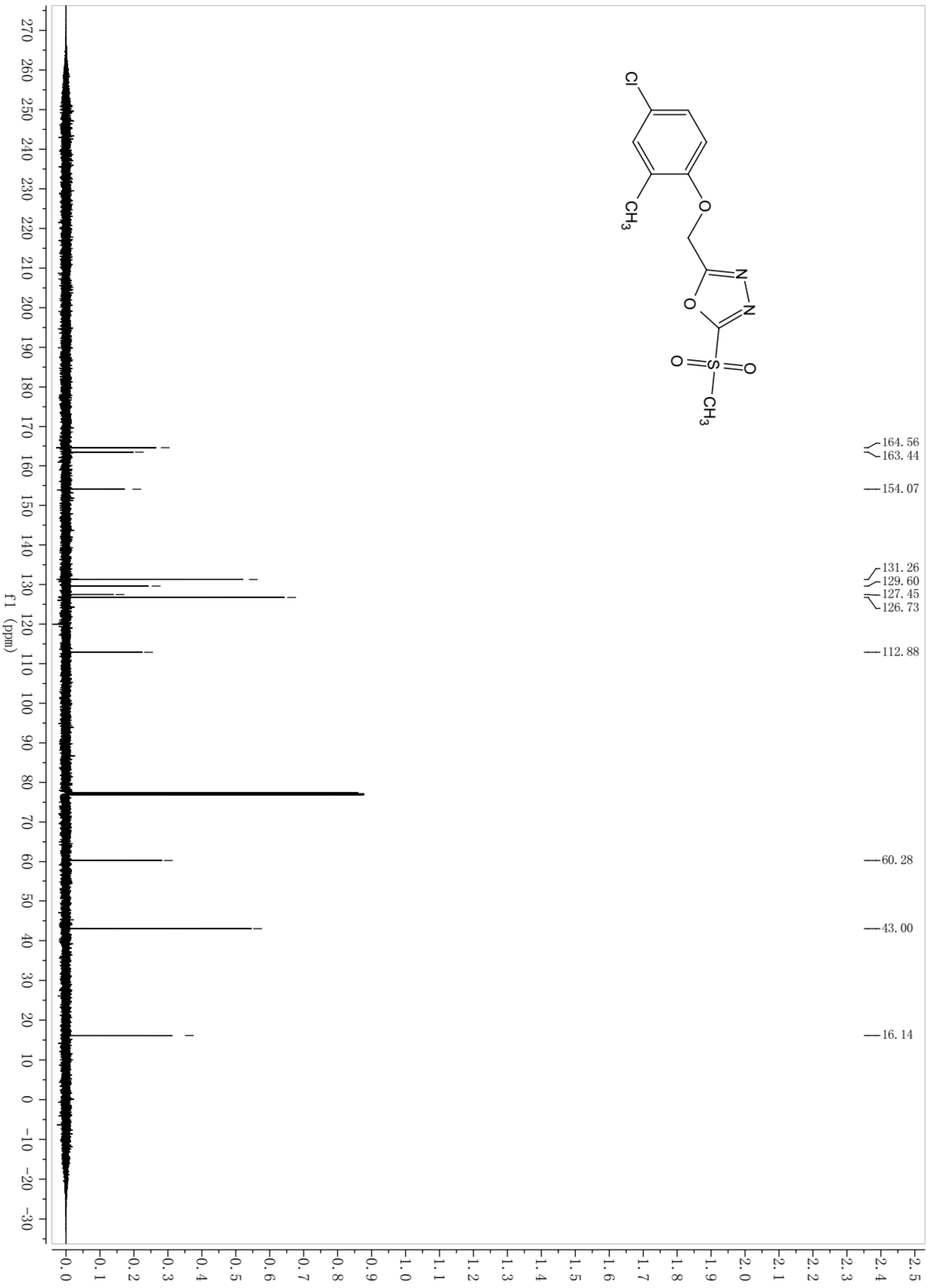

Figure S10. ^{13}C NMR spectrum of compound **5I-5**.

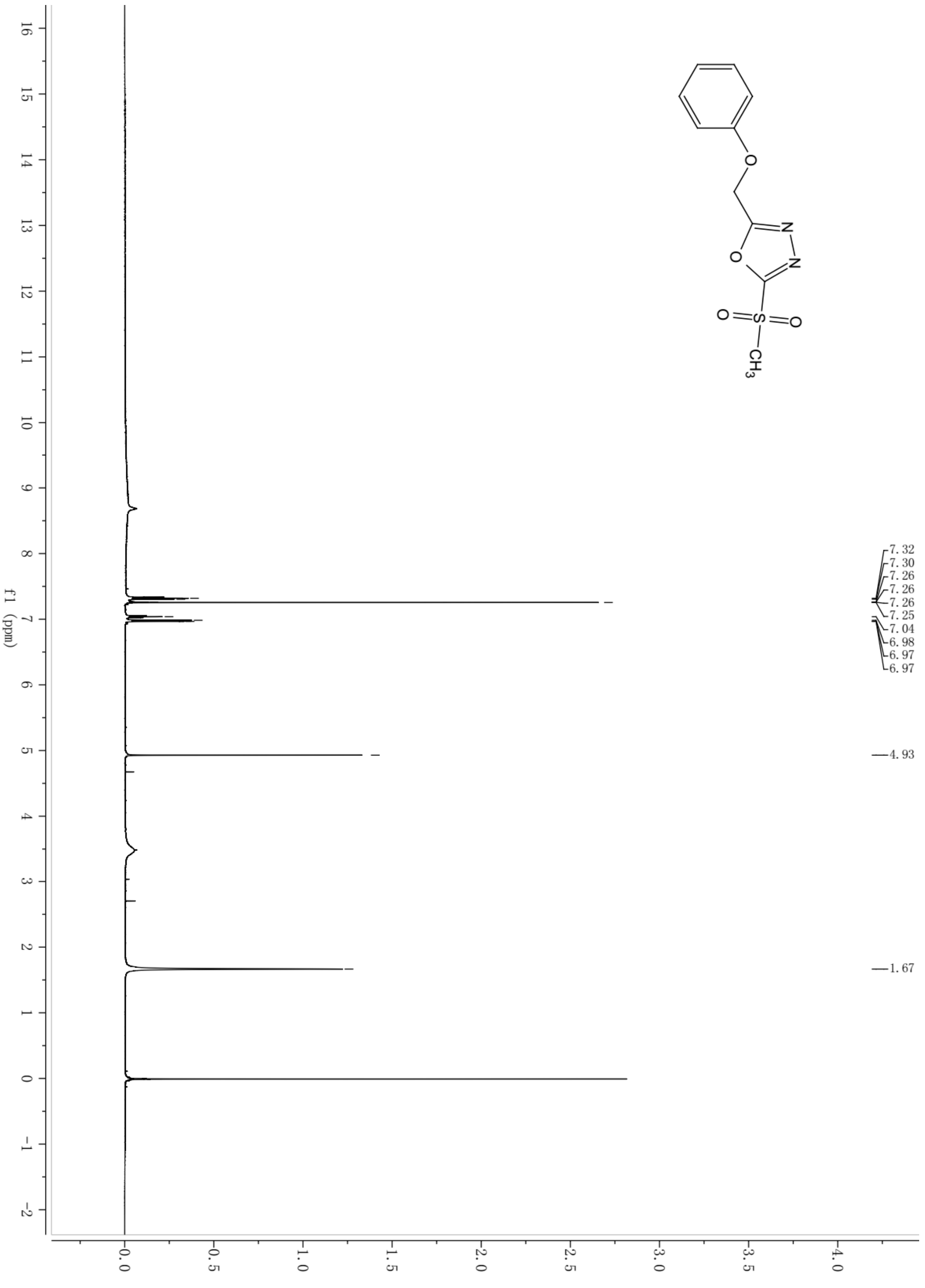

Figure S11. ^{1}H NMR spectrum of compound **5I-6**.

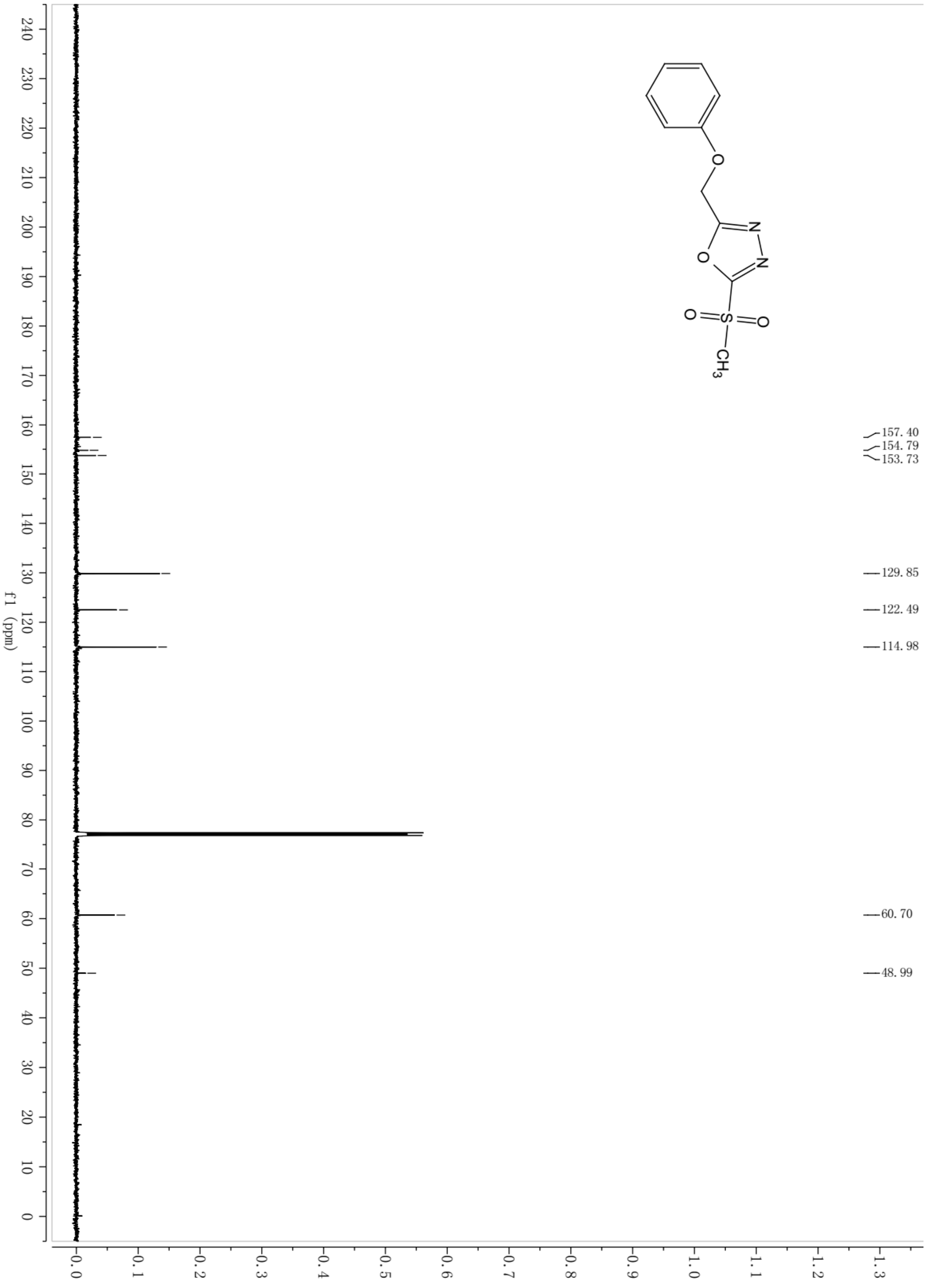

Figure S12. ^{13}C NMR spectrum of compound **5I-6**.

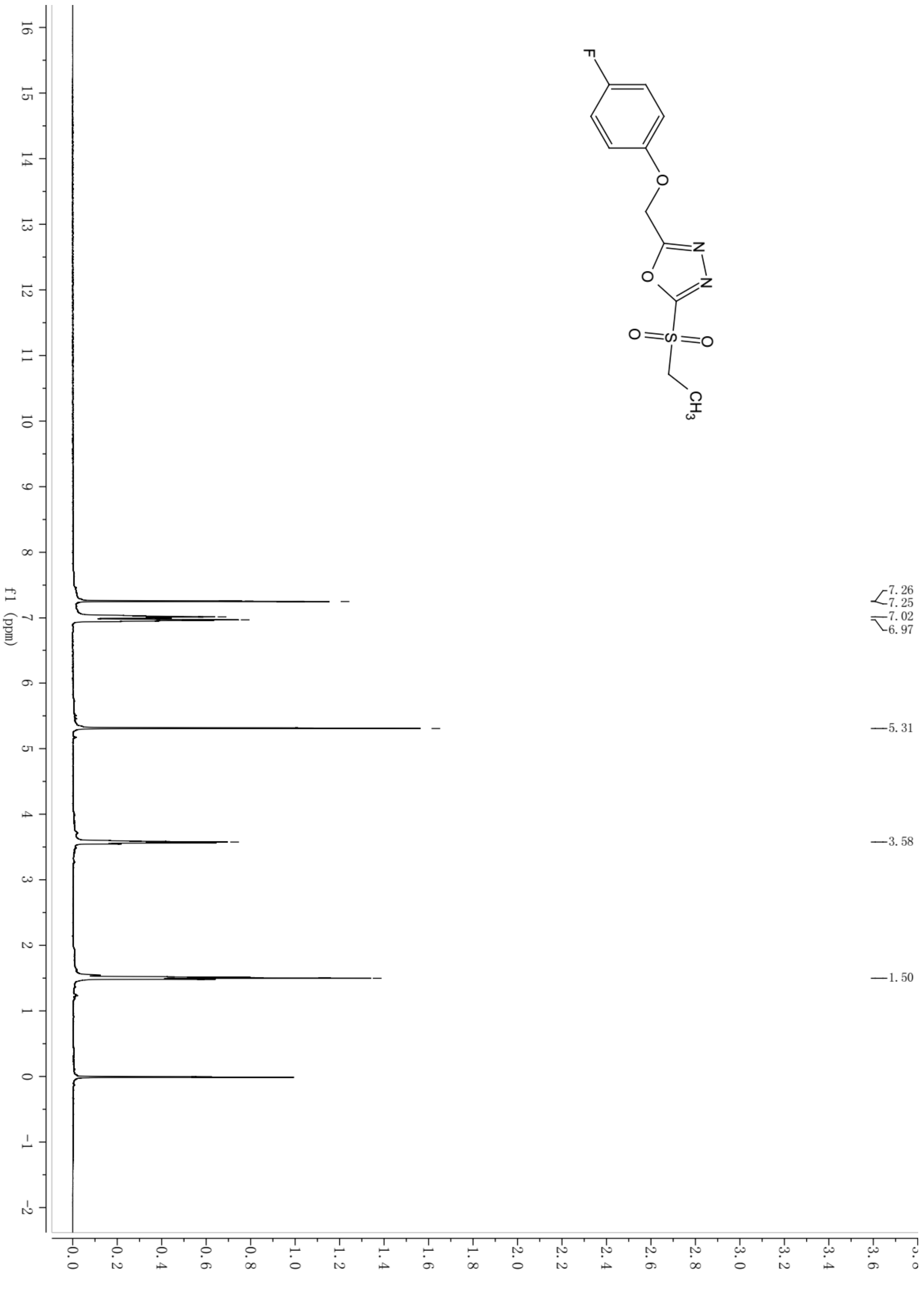

Figure S13. ^{1}H NMR spectrum of compound **5I-7**.

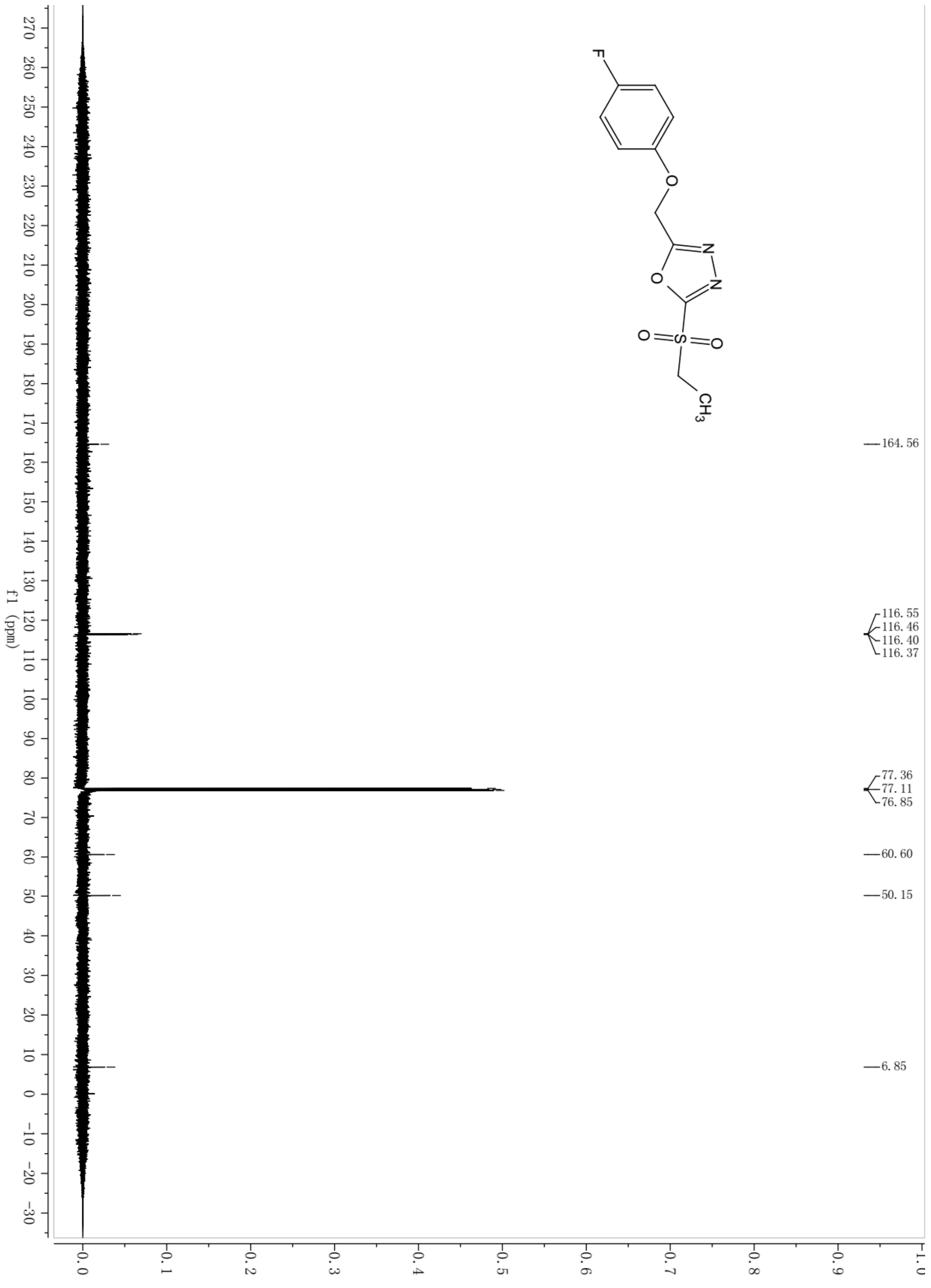

Figure S14. ^{13}C NMR spectrum of compound **5I-7**.

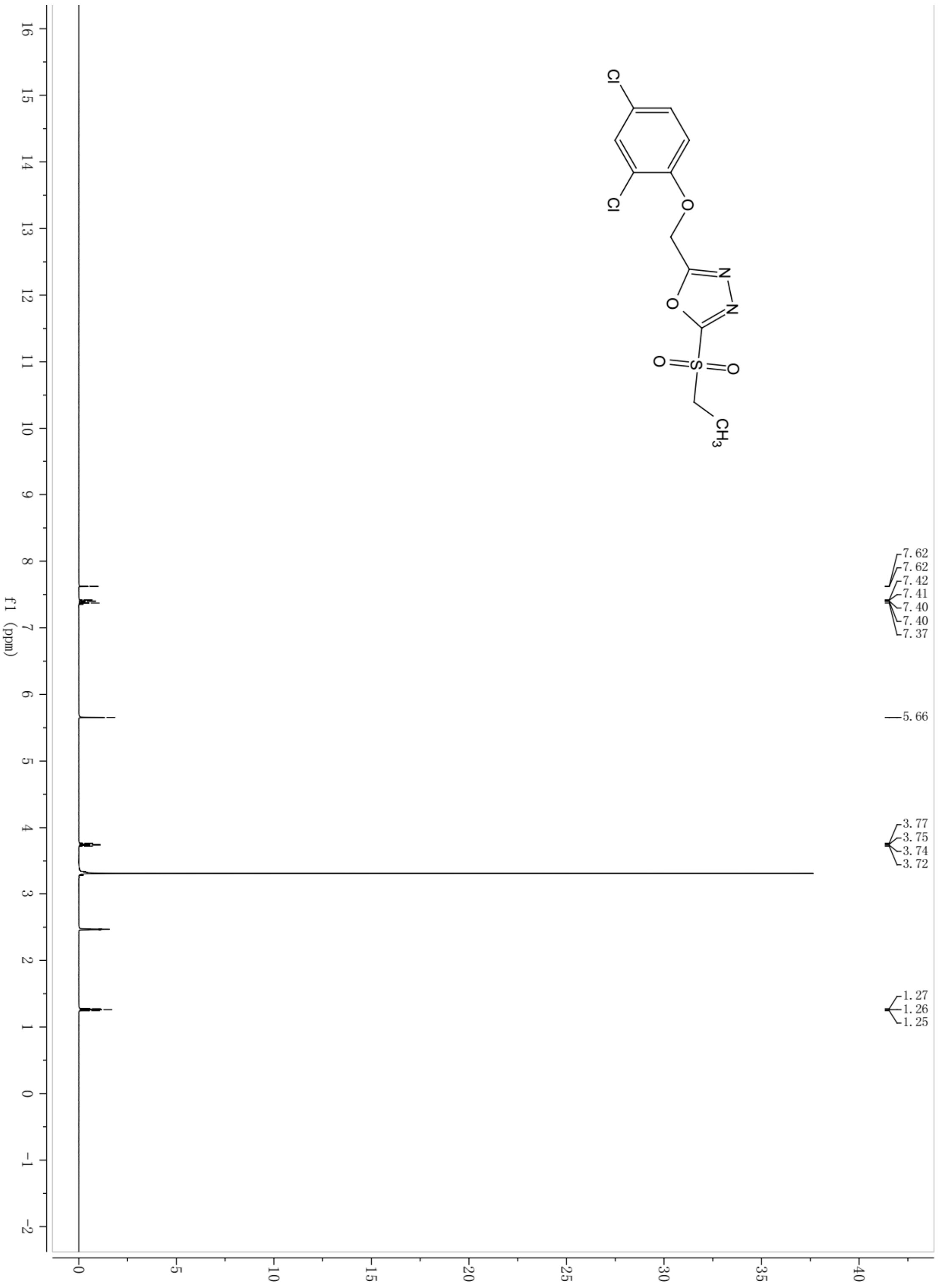

Figure S15. ¹H NMR spectrum of compound **5I-8**.

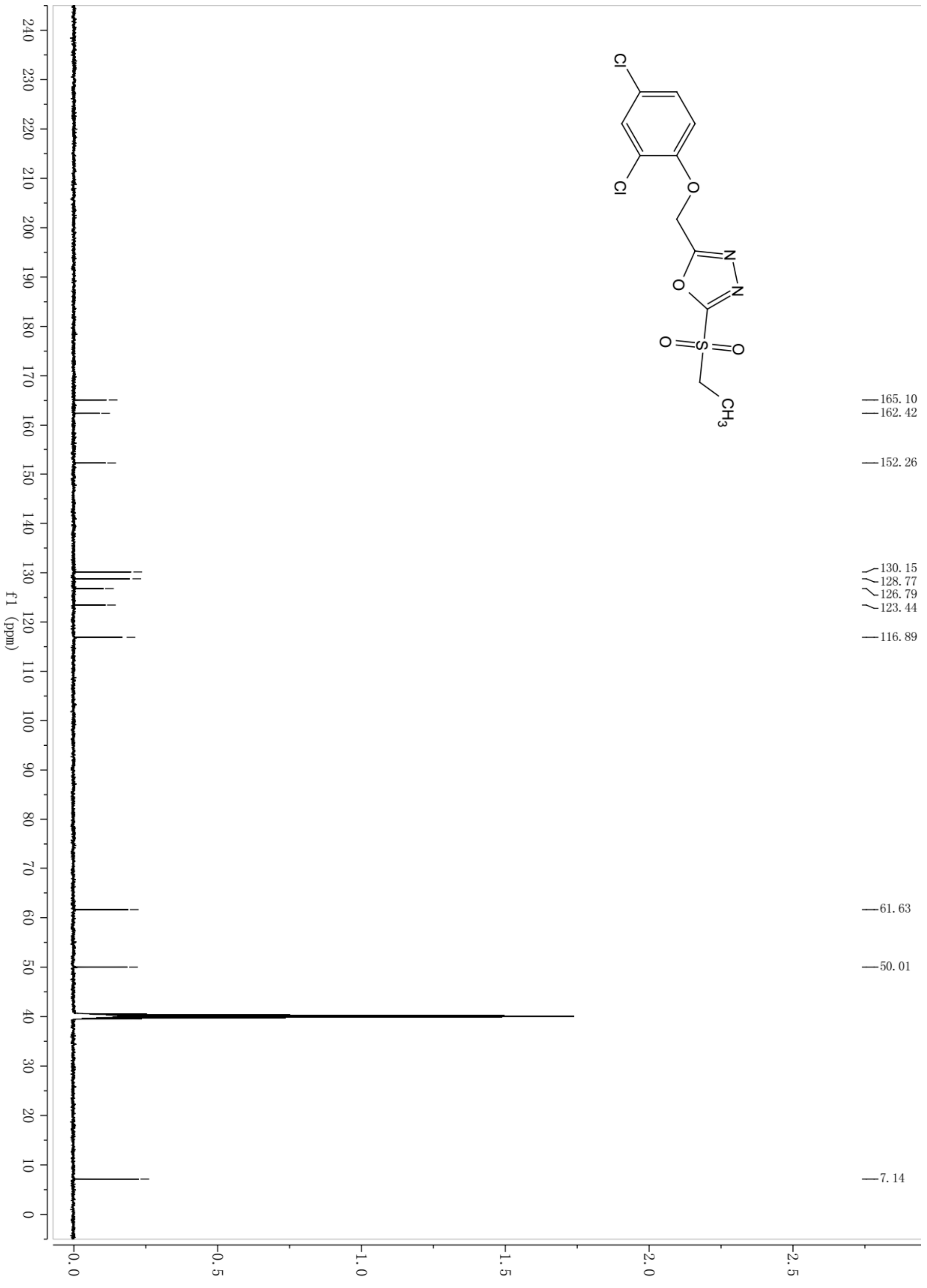

Figure S16. ^{13}C NMR spectrum of compound **5I-8**.

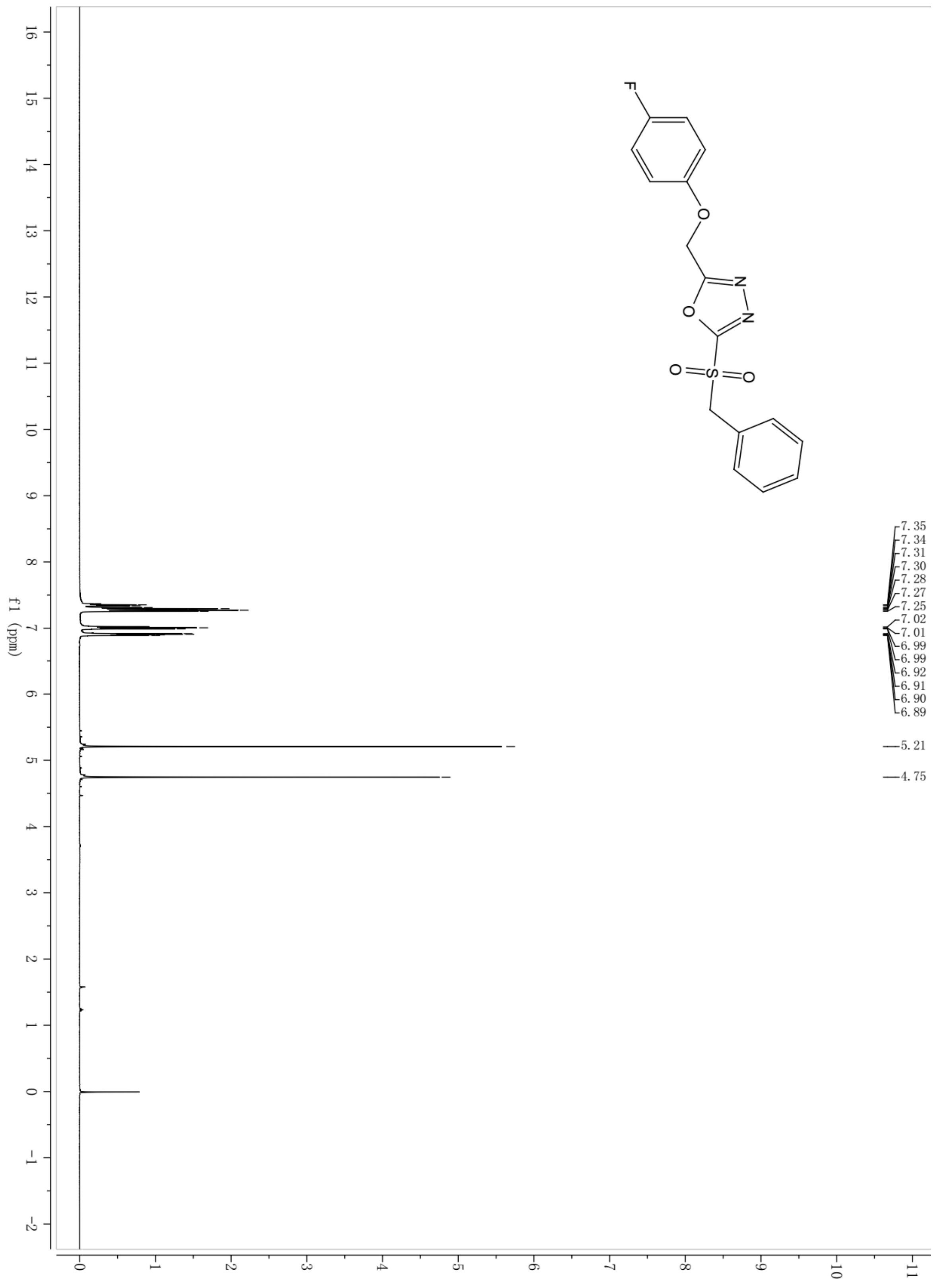

Figure S17. ^{1}H NMR spectrum of compound **5II-9**.

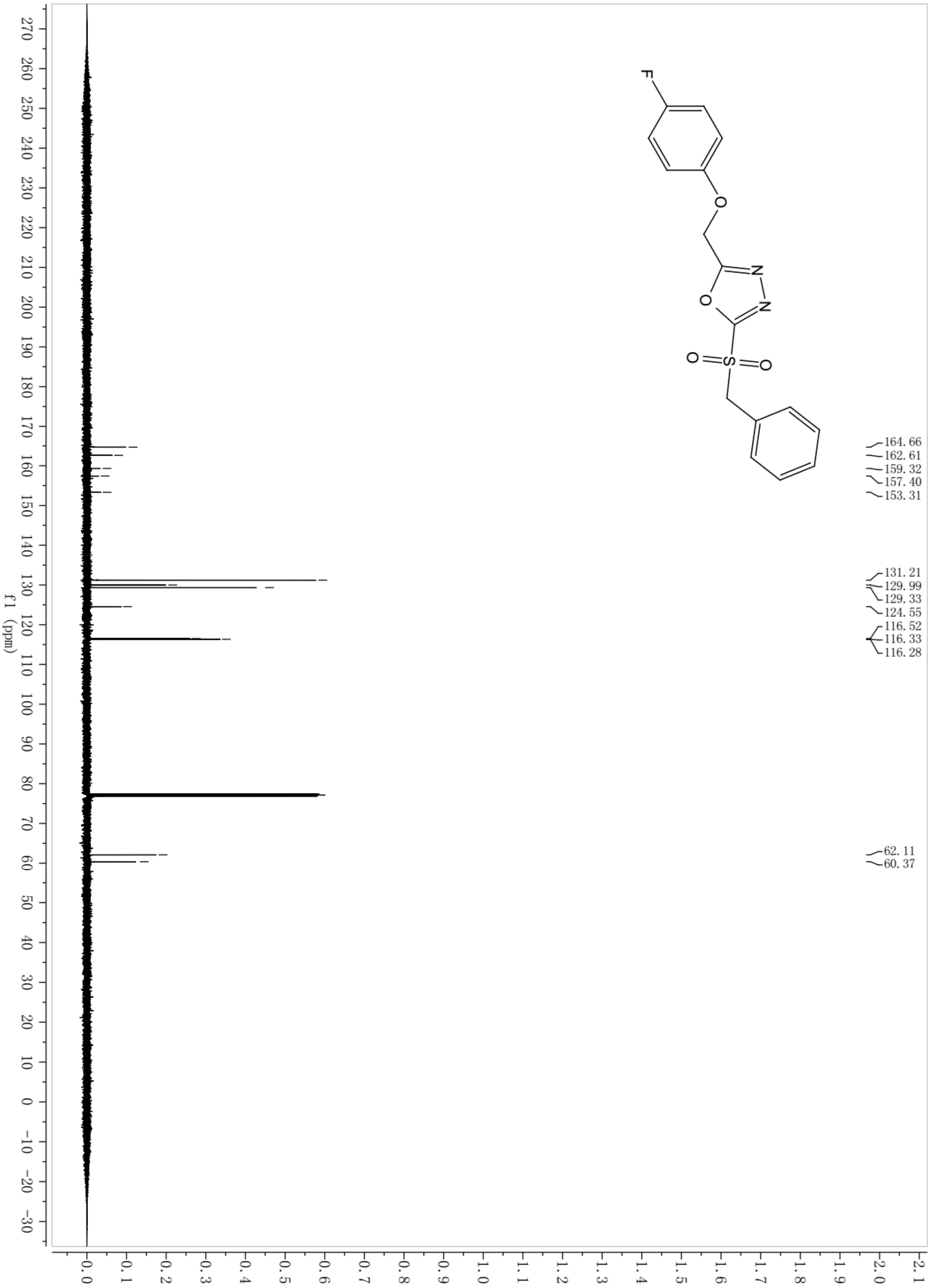

Figure S18. ^{13}C NMR spectrum of compound **5II-9**.

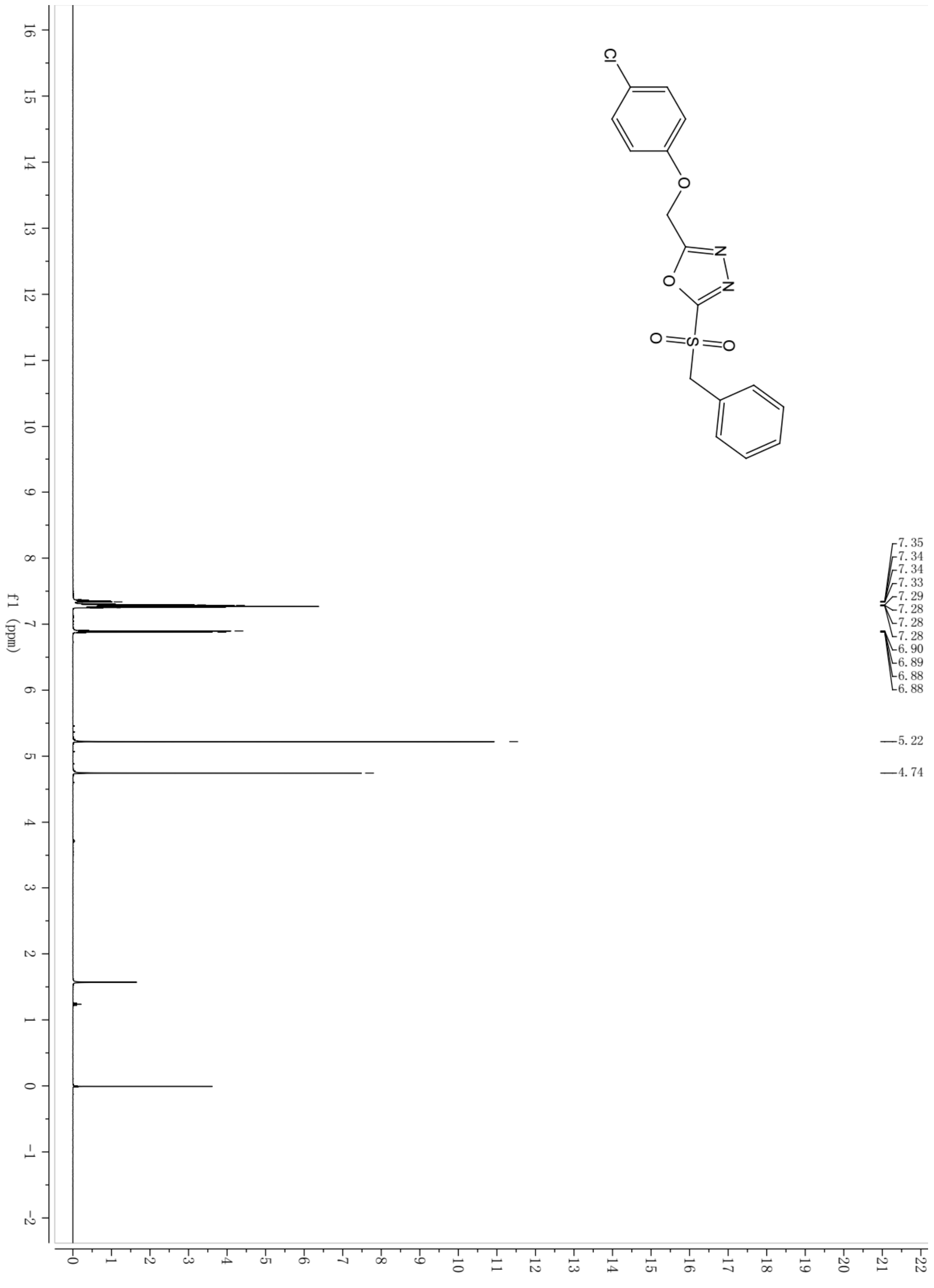

Figure S19. ^{1}H NMR spectrum of compound **5I-10**.

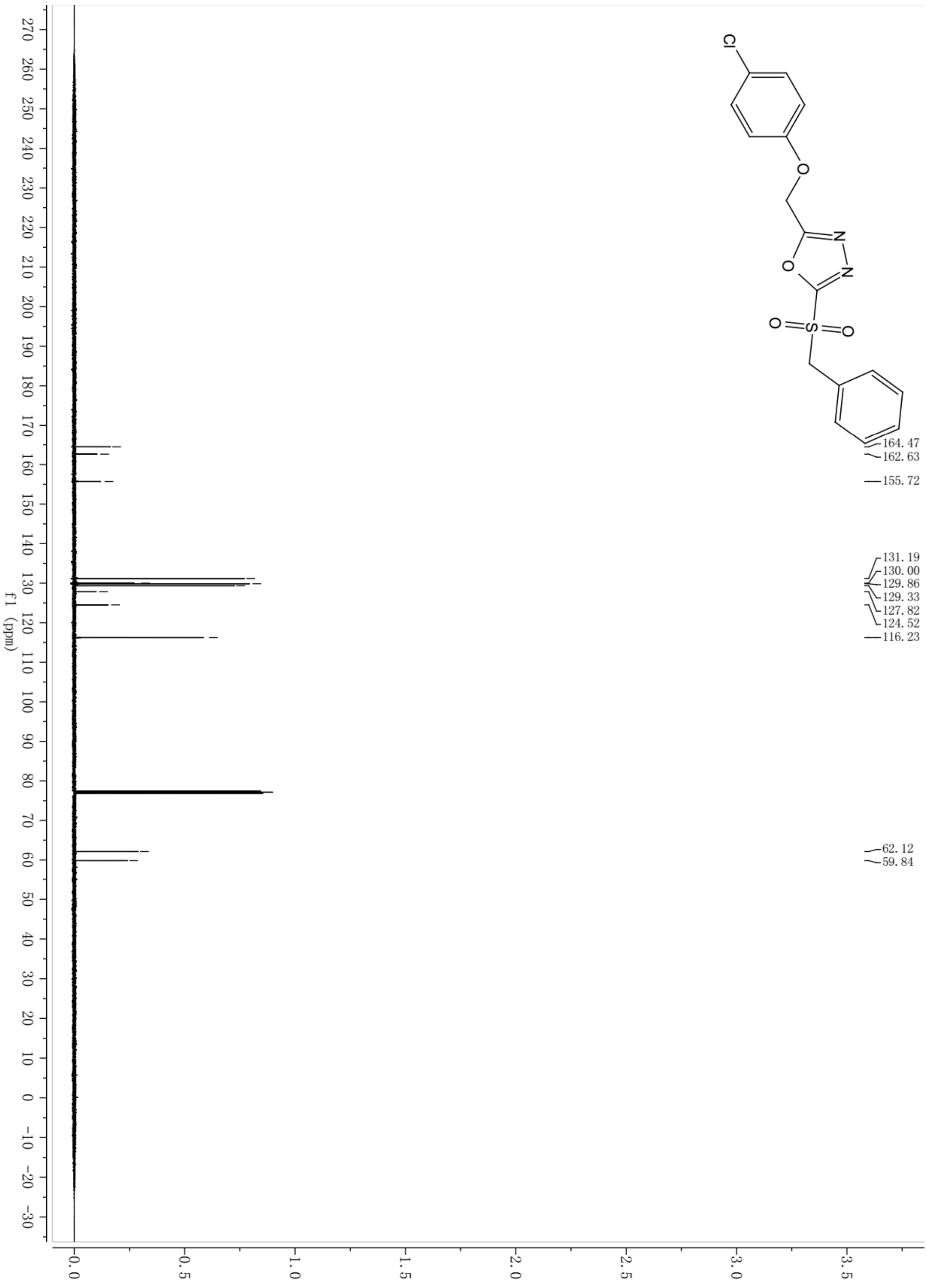

Figure S20. ^{13}C NMR spectrum of compound **5I-10**.

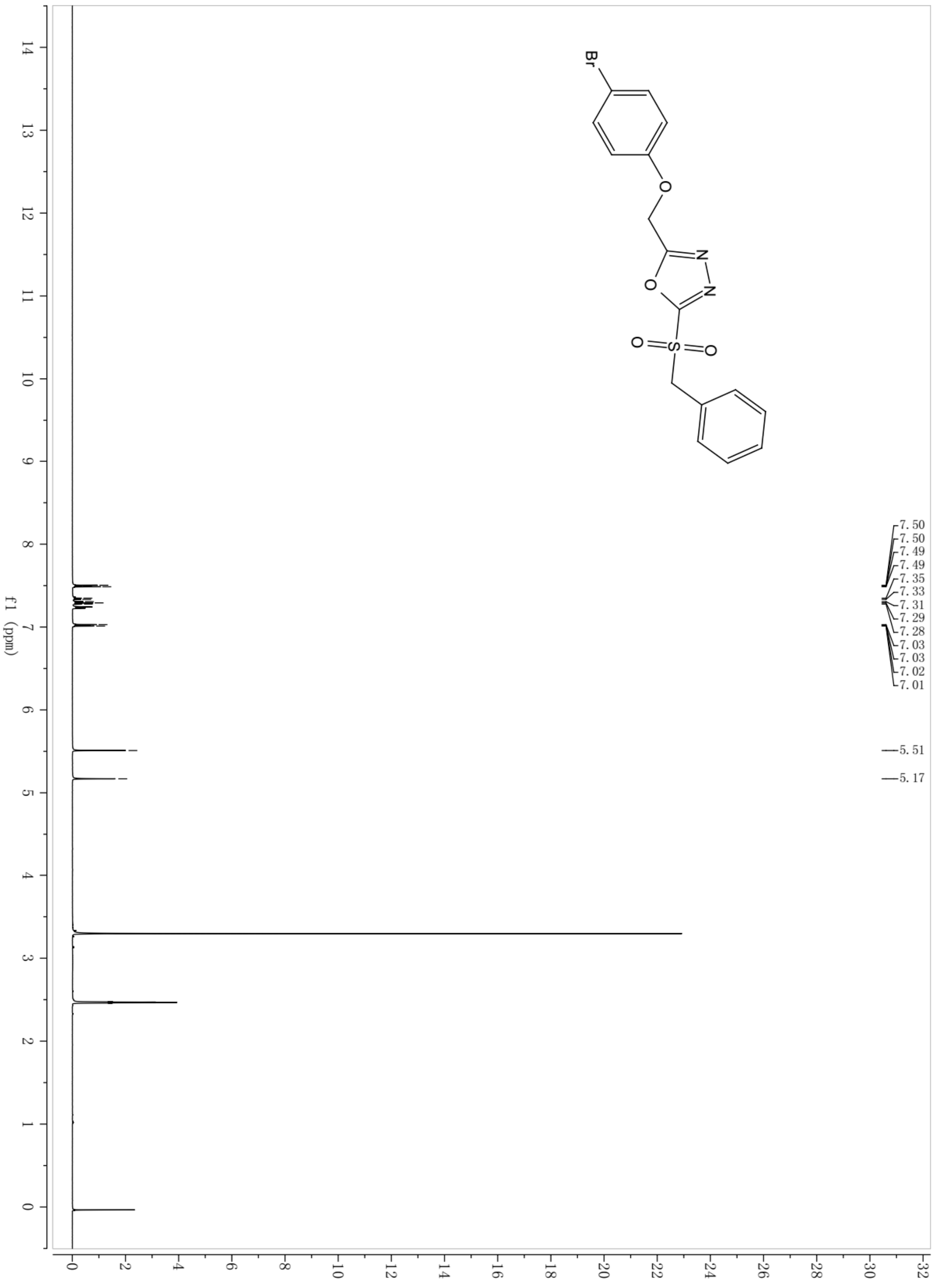

Figure S21. ^{1}H NMR spectrum of compound **5I-11**.

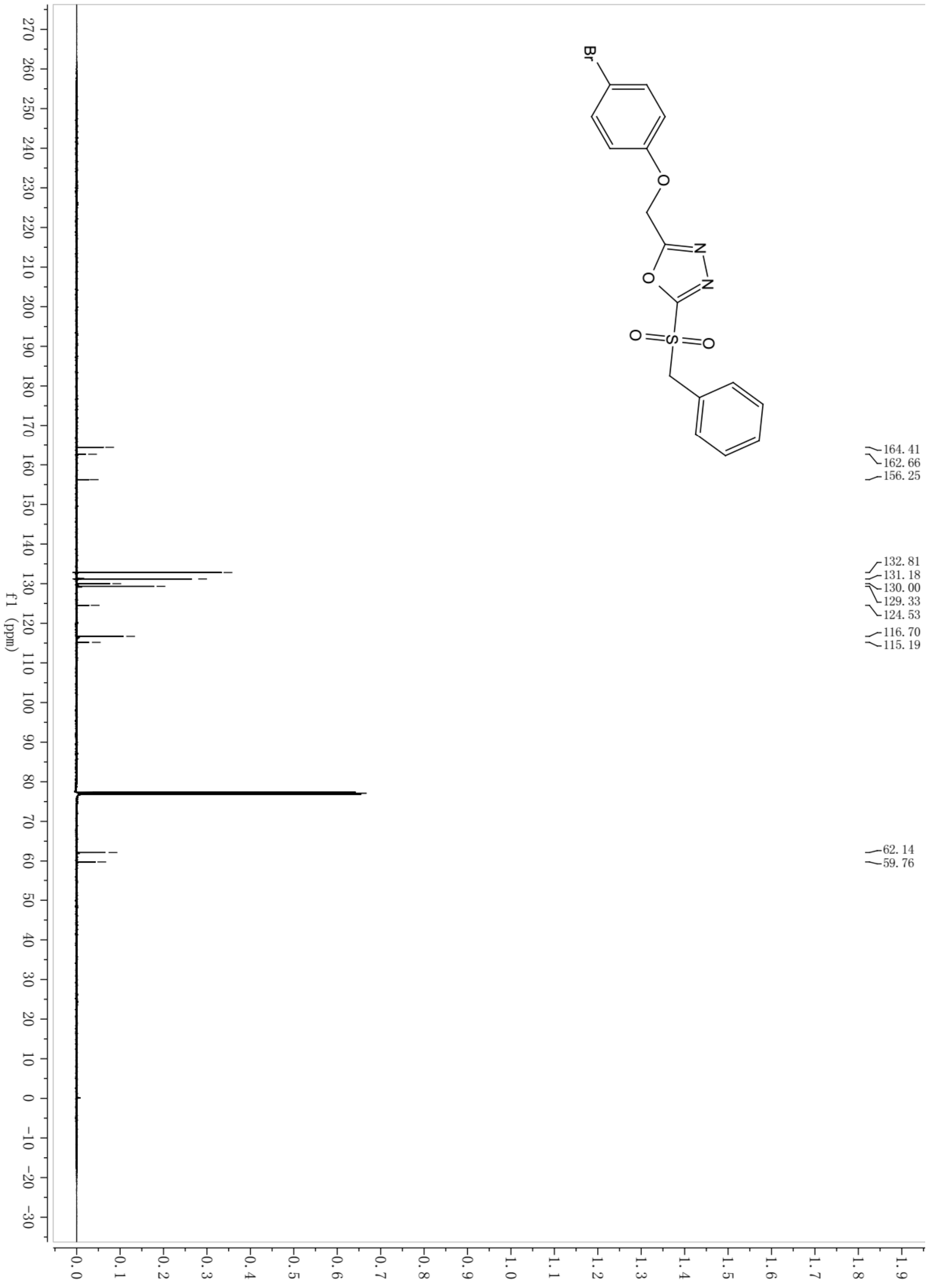

Figure S22. ^{13}C NMR spectrum of compound **5I-11**.

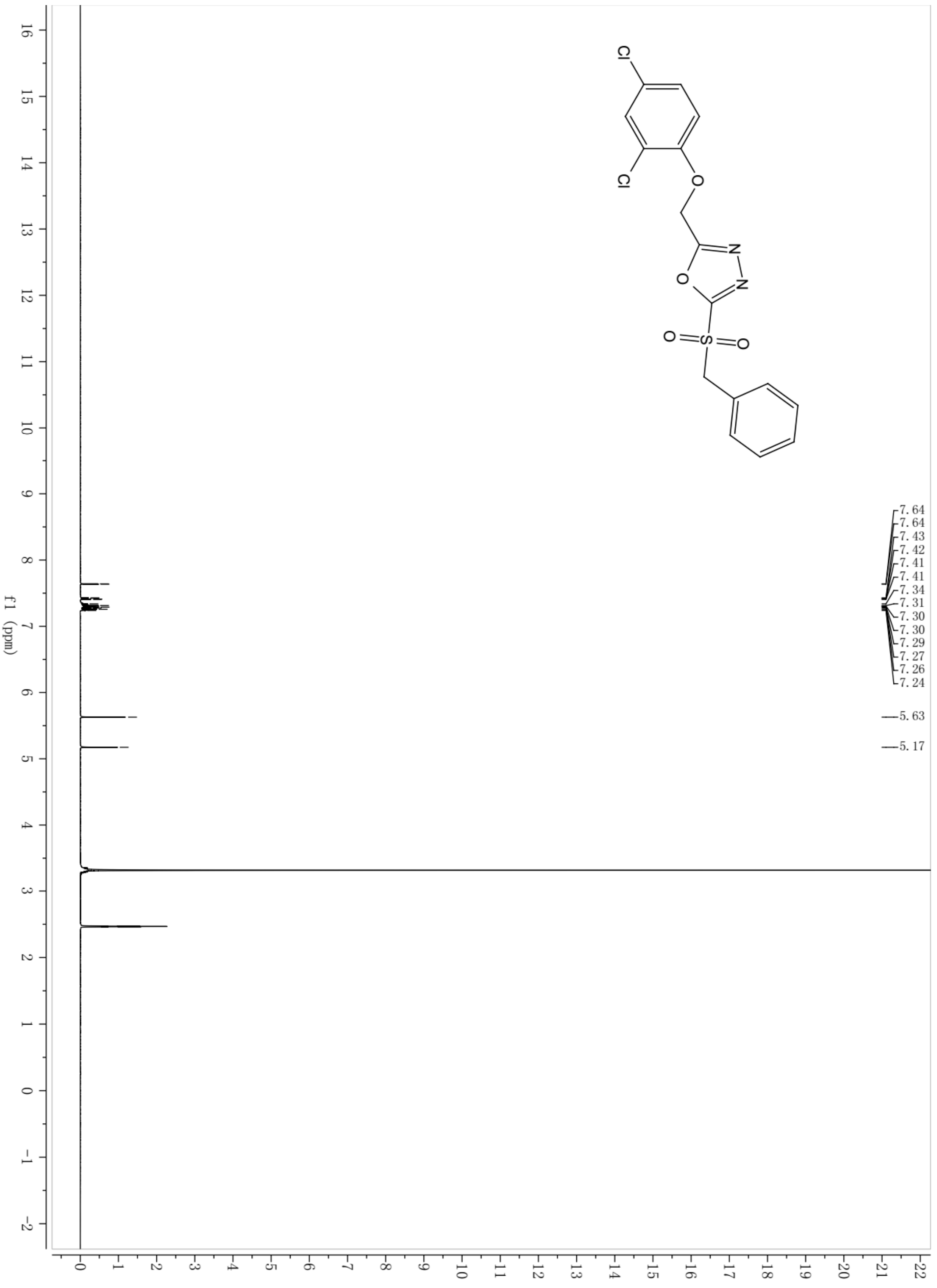

Figure S23. ^{1}H NMR spectrum of compound **5I-12**.

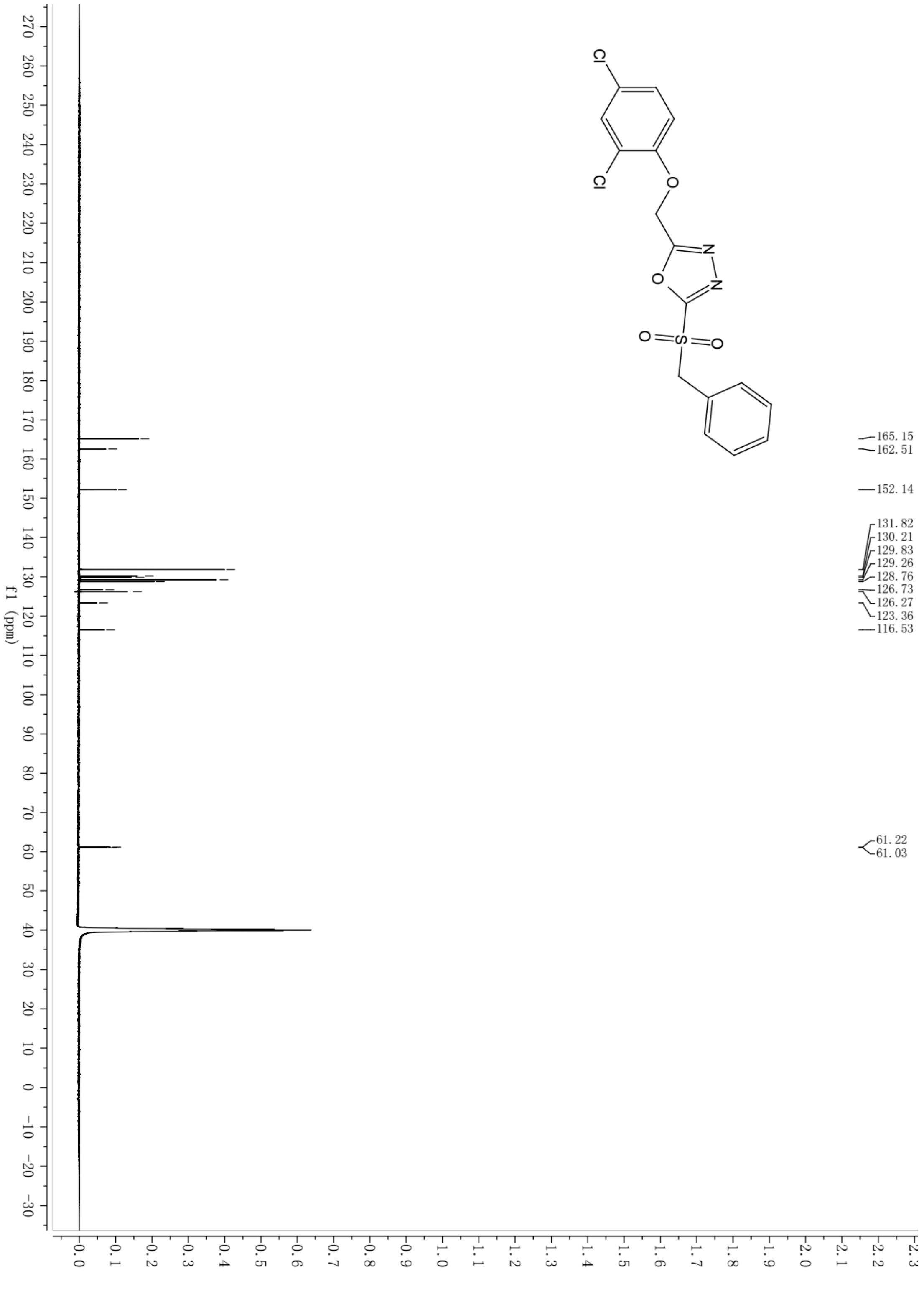

Figure S24. ^{13}C NMR spectrum of compound **5I-12**.

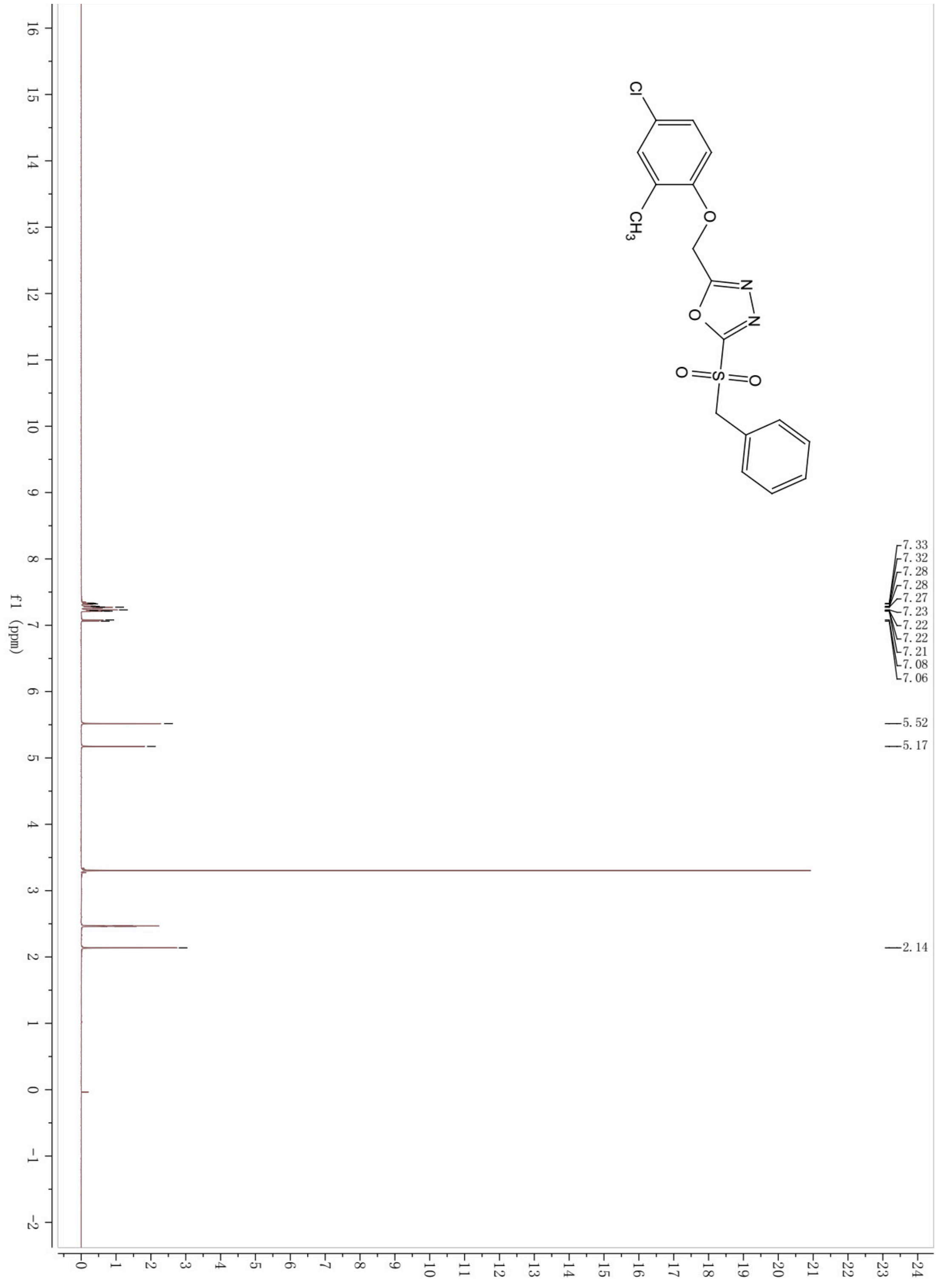

Figure S25. ^{1}H NMR spectrum of compound **5I-13**.

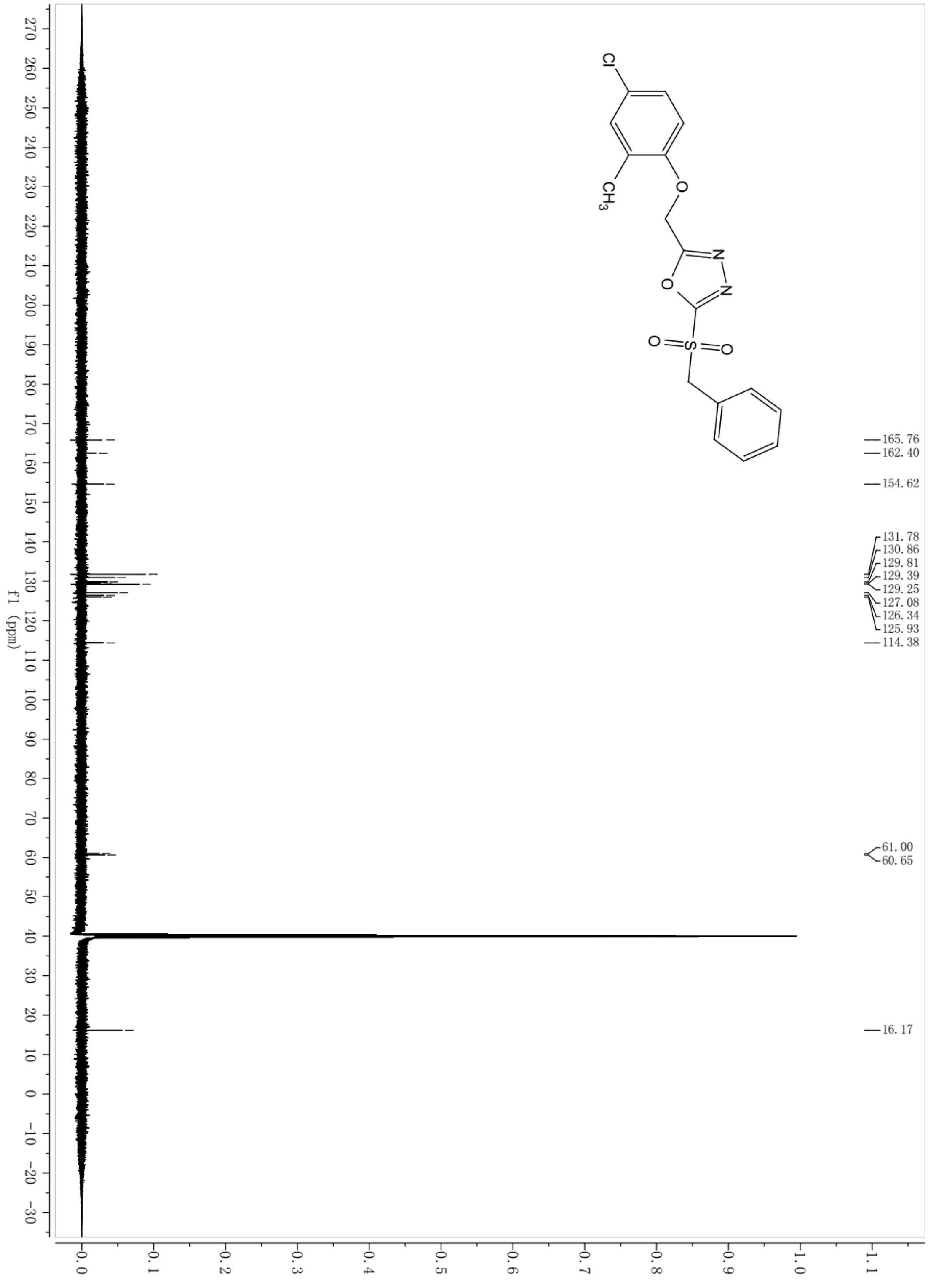

Figure S26. ^{13}C NMR spectrum of compound **5I-13**.

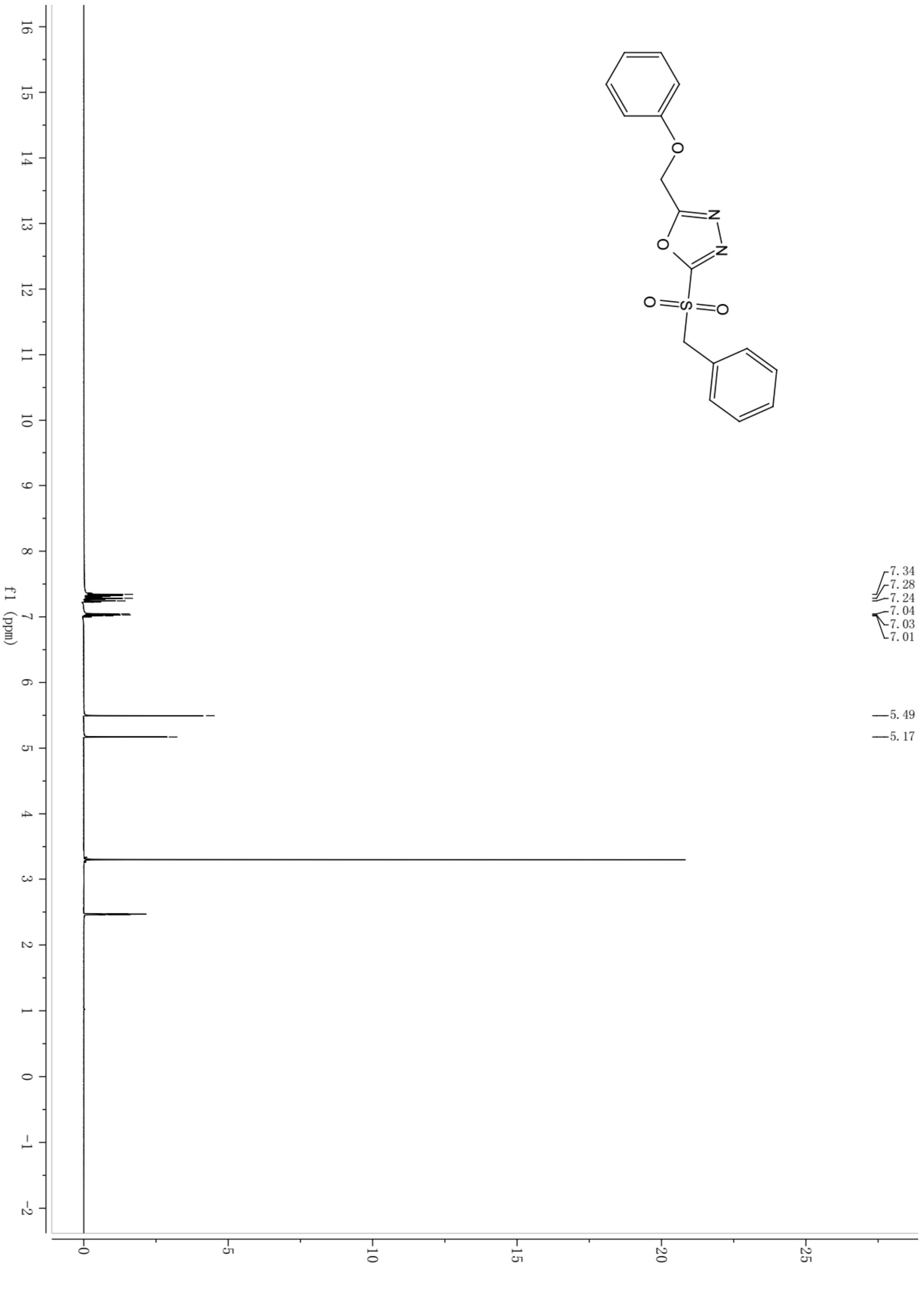

Figure S27. ^{1}H NMR spectrum of compound **5I-14**.

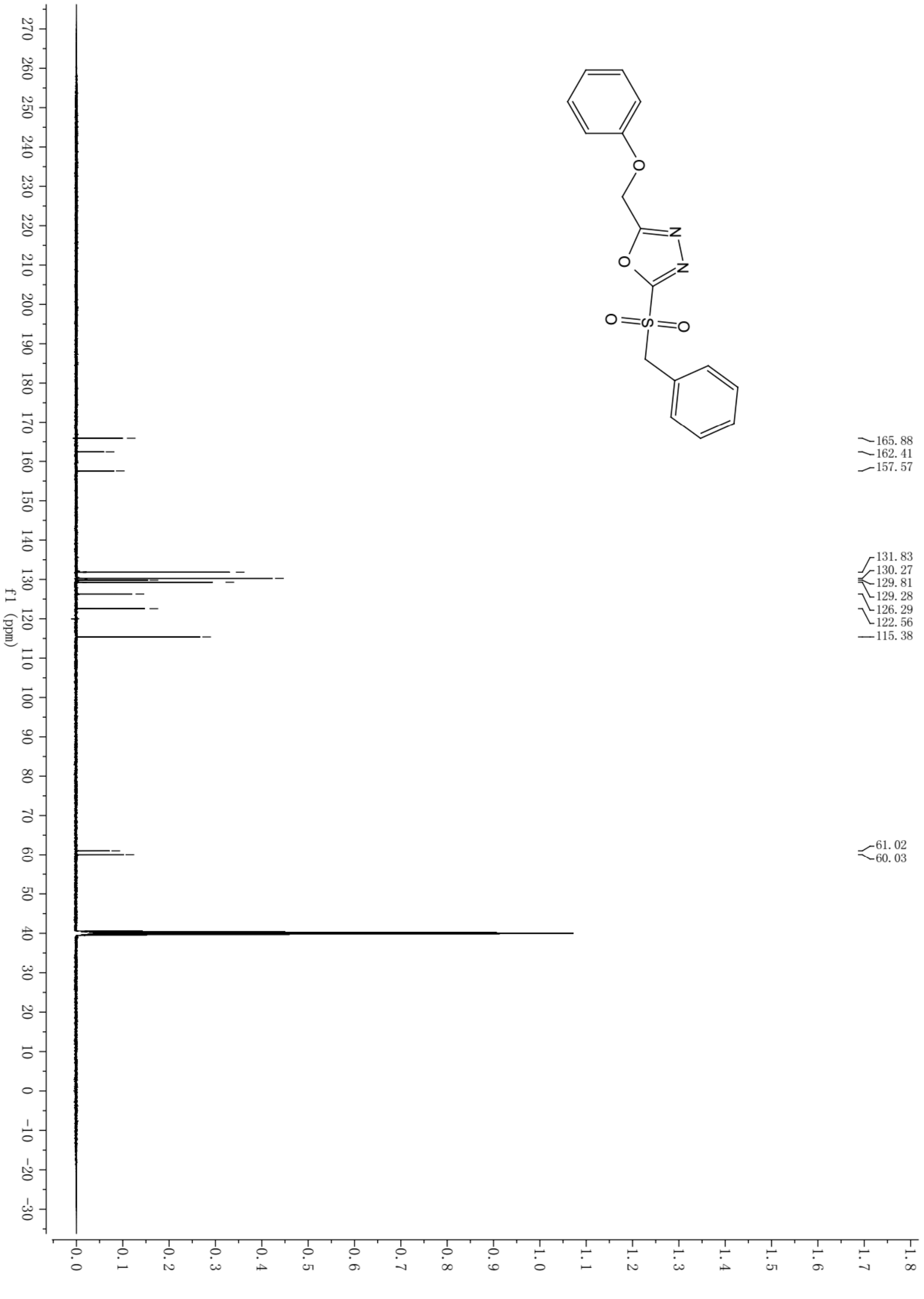

Figure S28. ^{13}C NMR spectrum of compound **5I-14**.

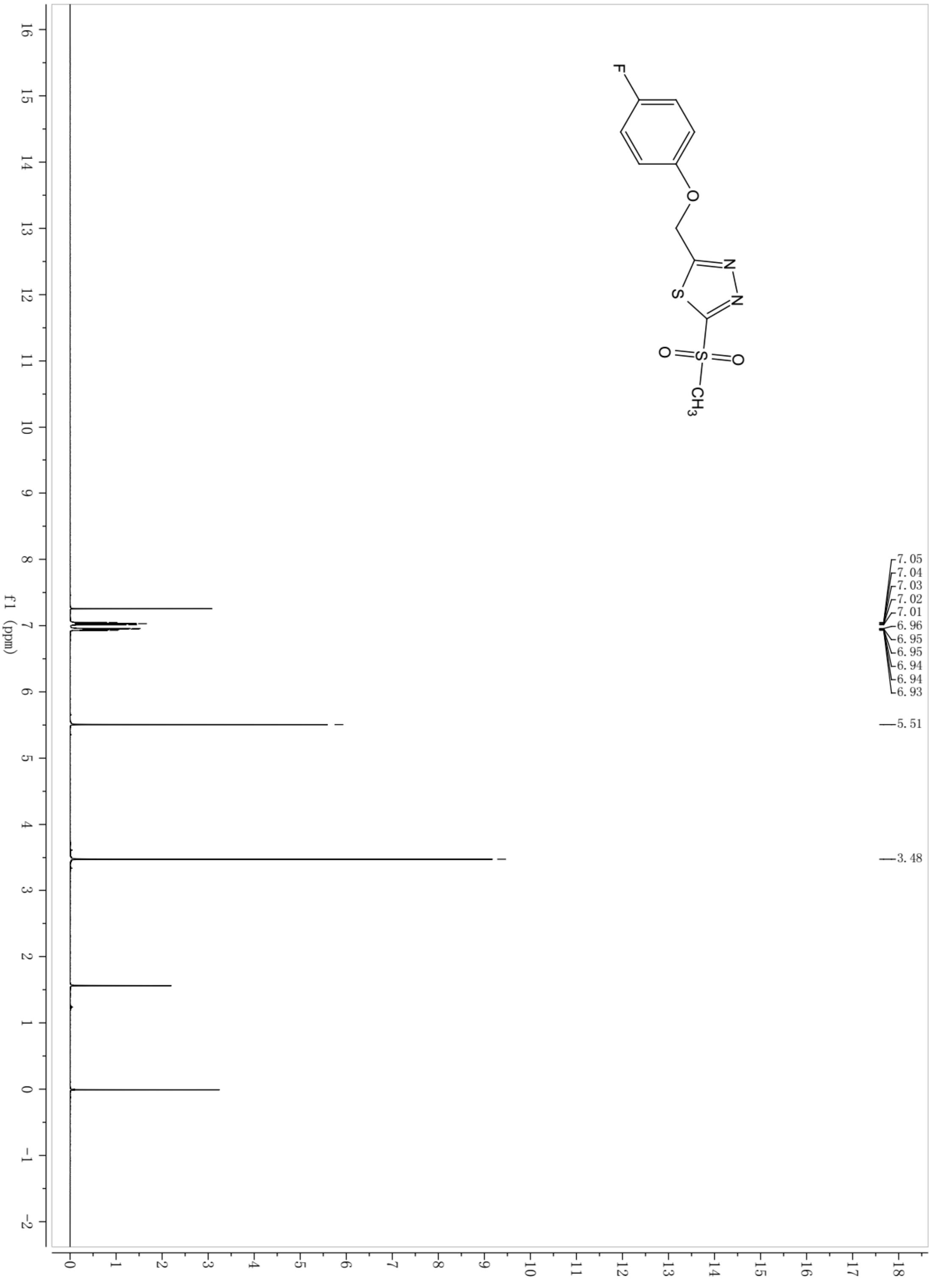

Figure S29. ^{1}H NMR spectrum of compound **5II-1**.

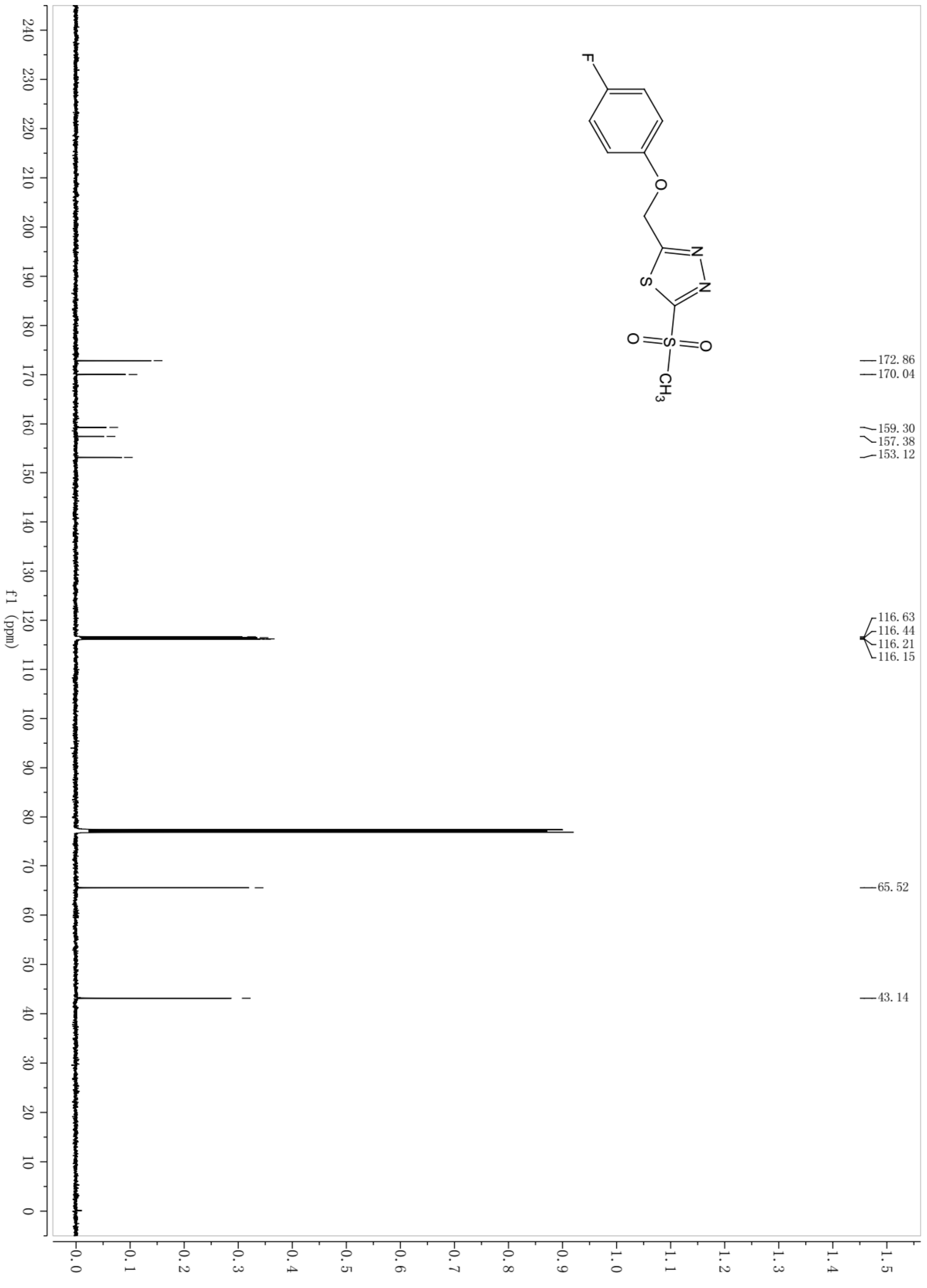

Figure S30. ^{13}C NMR spectrum of compound **5II-1**.

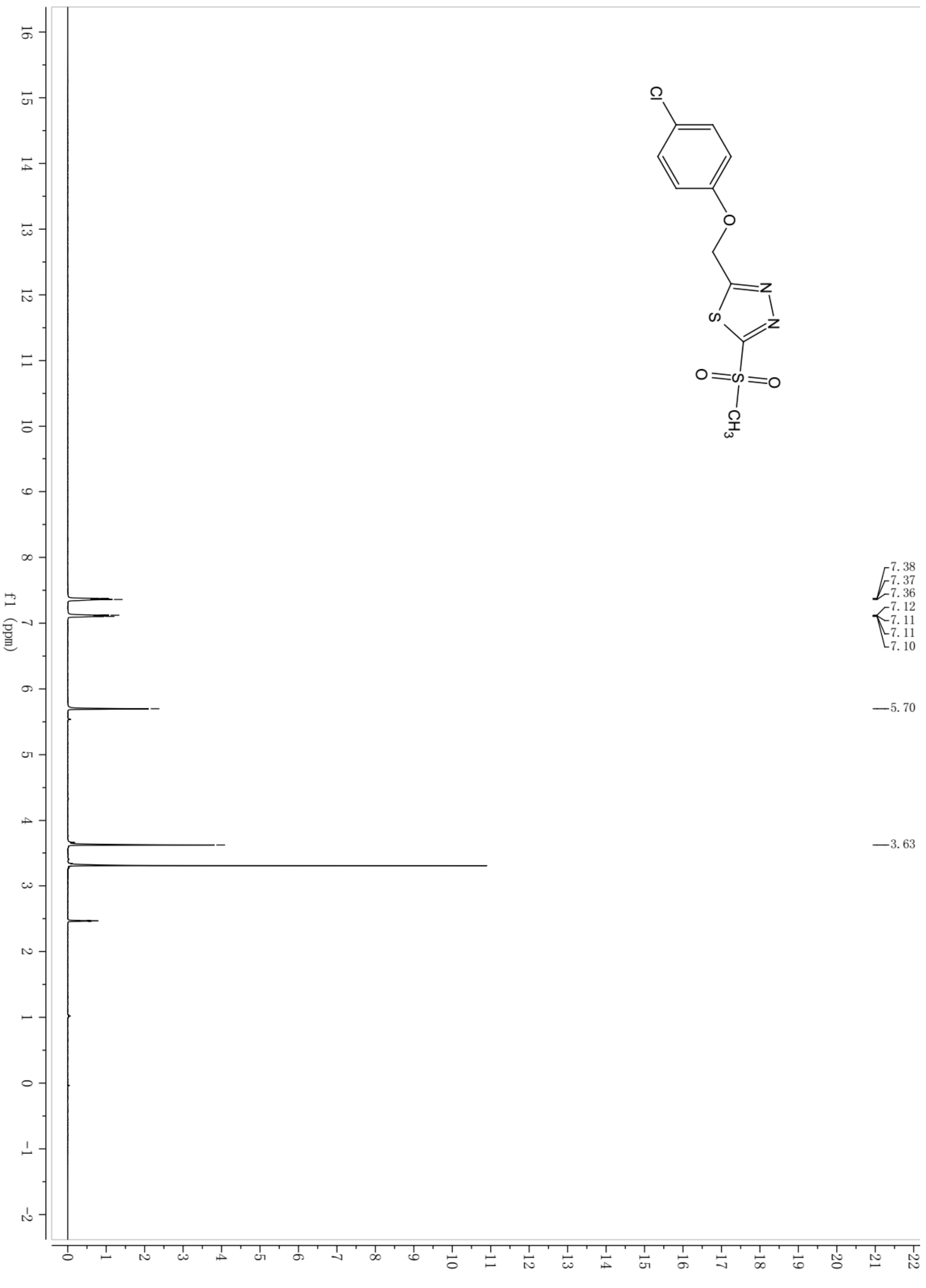

Figure S31. ^{1}H NMR spectrum of compound **5II-2**.

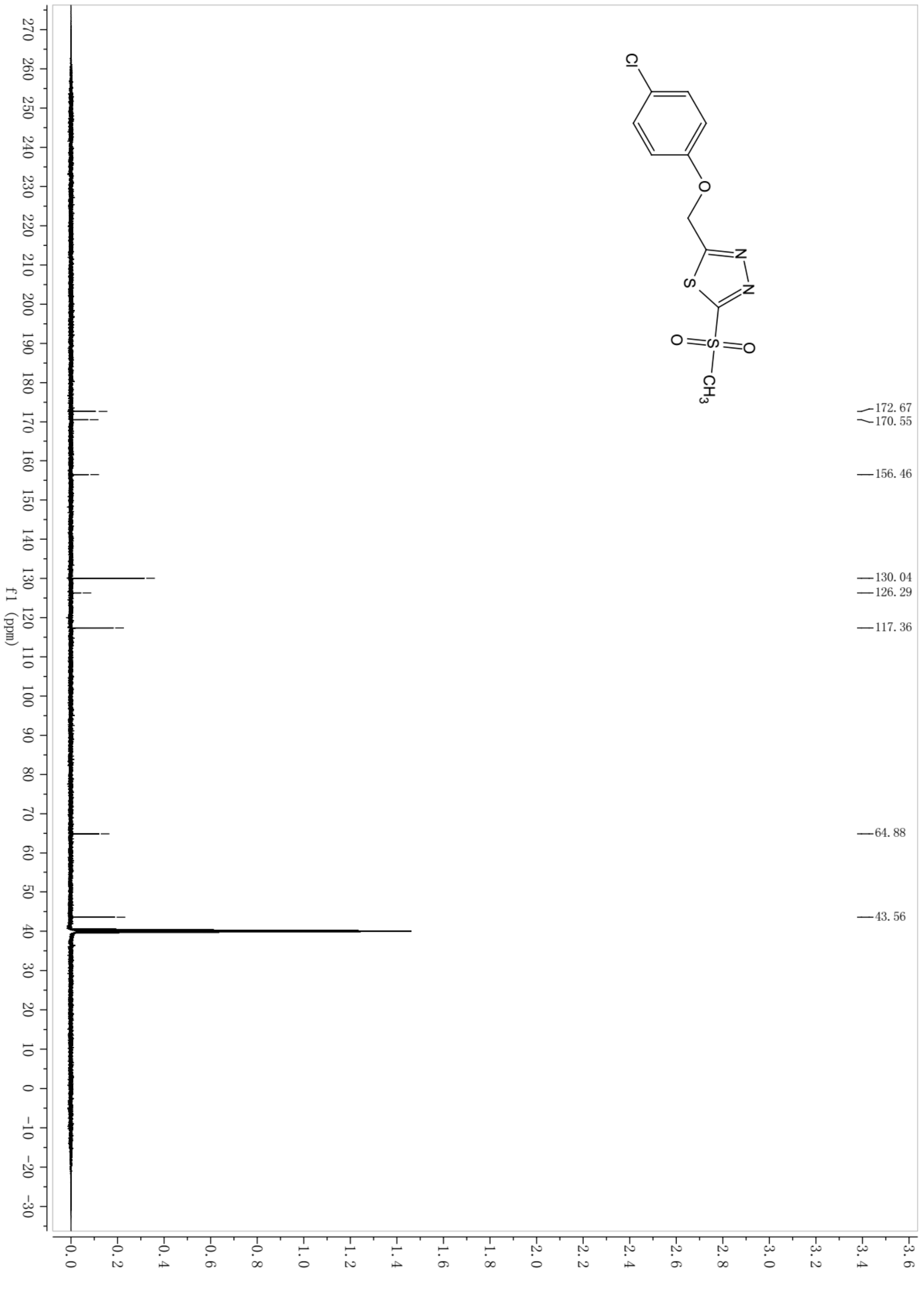

Figure S32. ^{13}C NMR spectrum of compound **5II-2**.

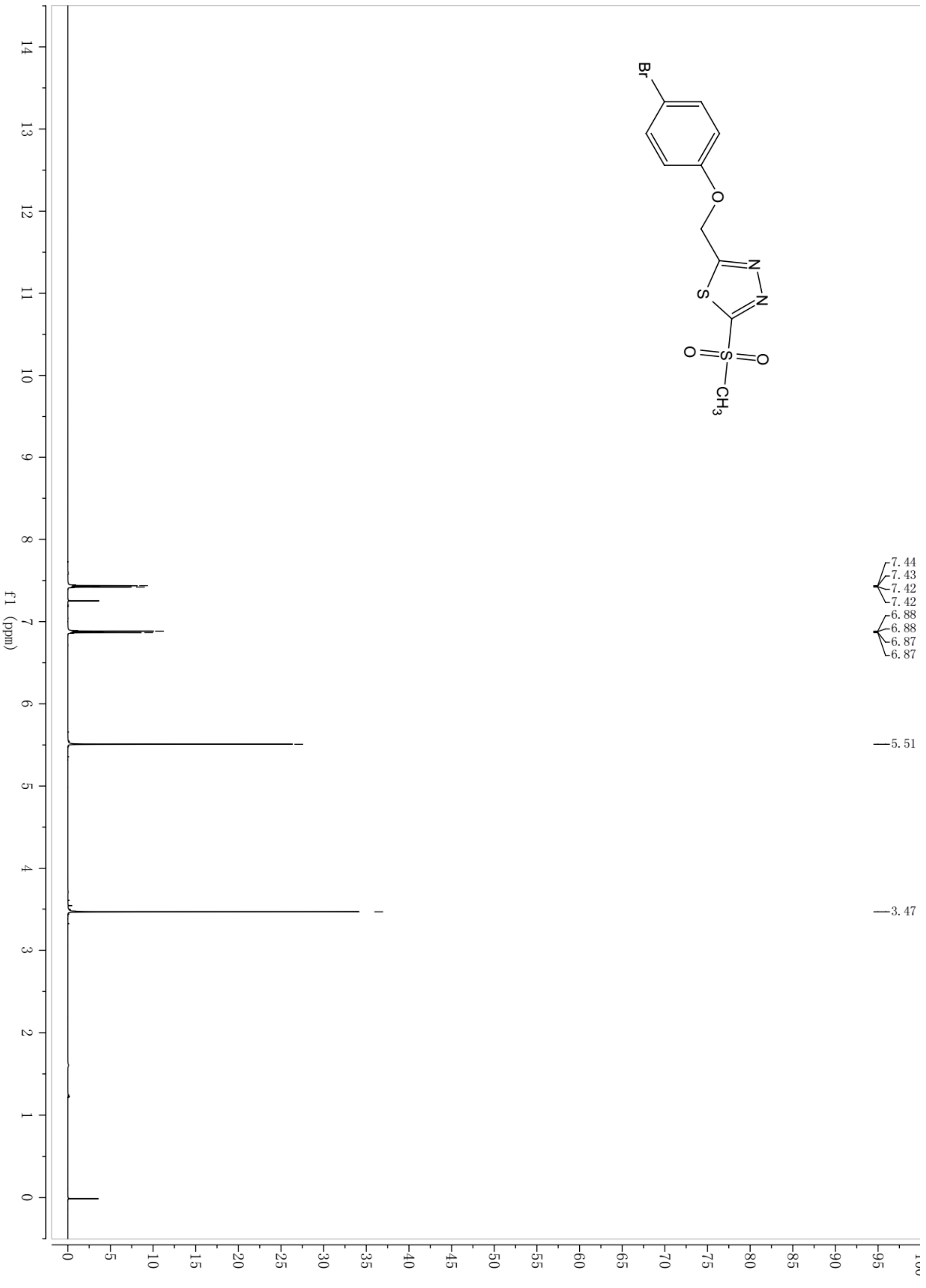

Figure S33. ^{1}H NMR spectrum of compound **5II-3**.

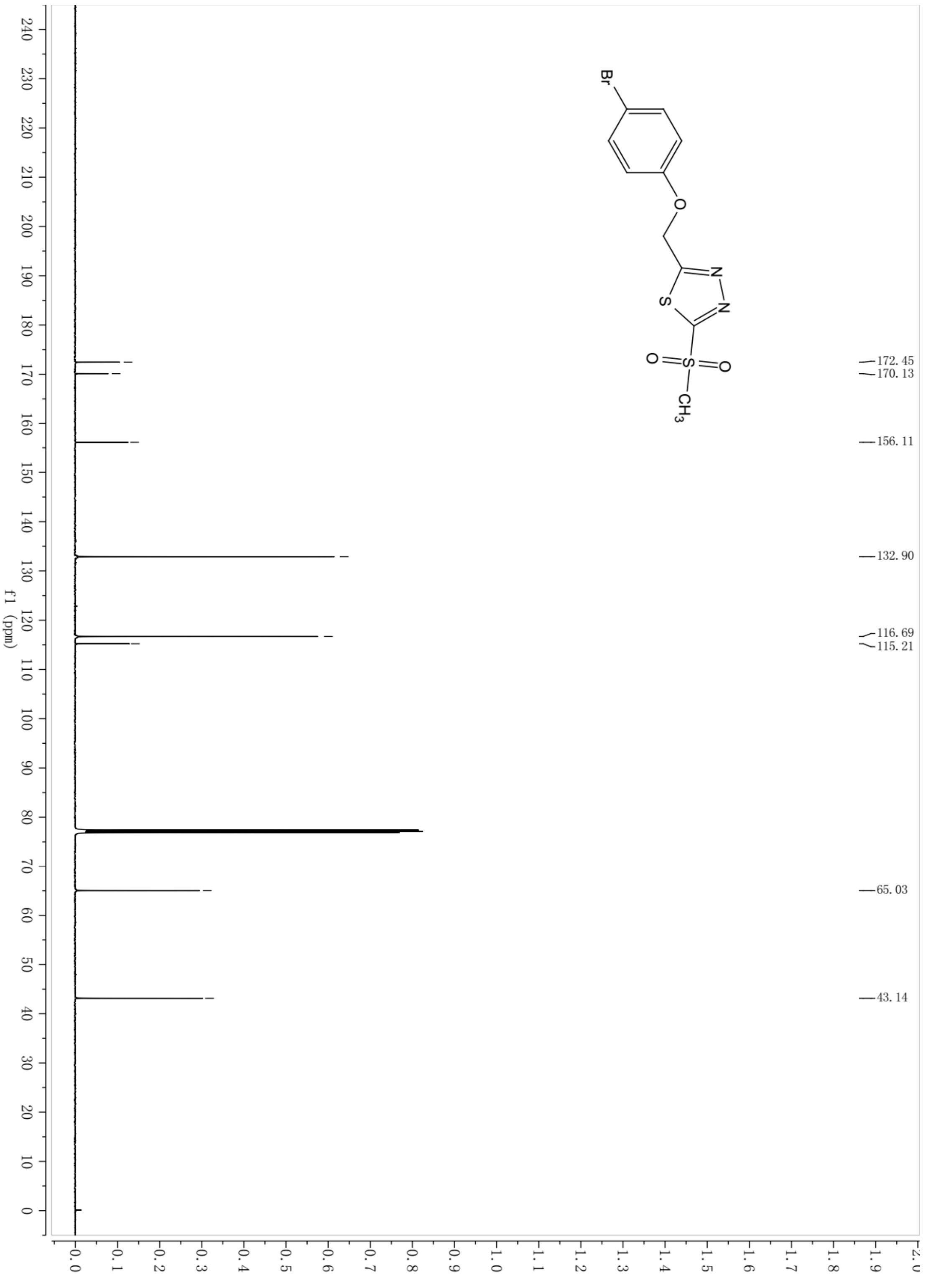

Figure S34. ^{13}C NMR spectrum of compound **5II-3**.

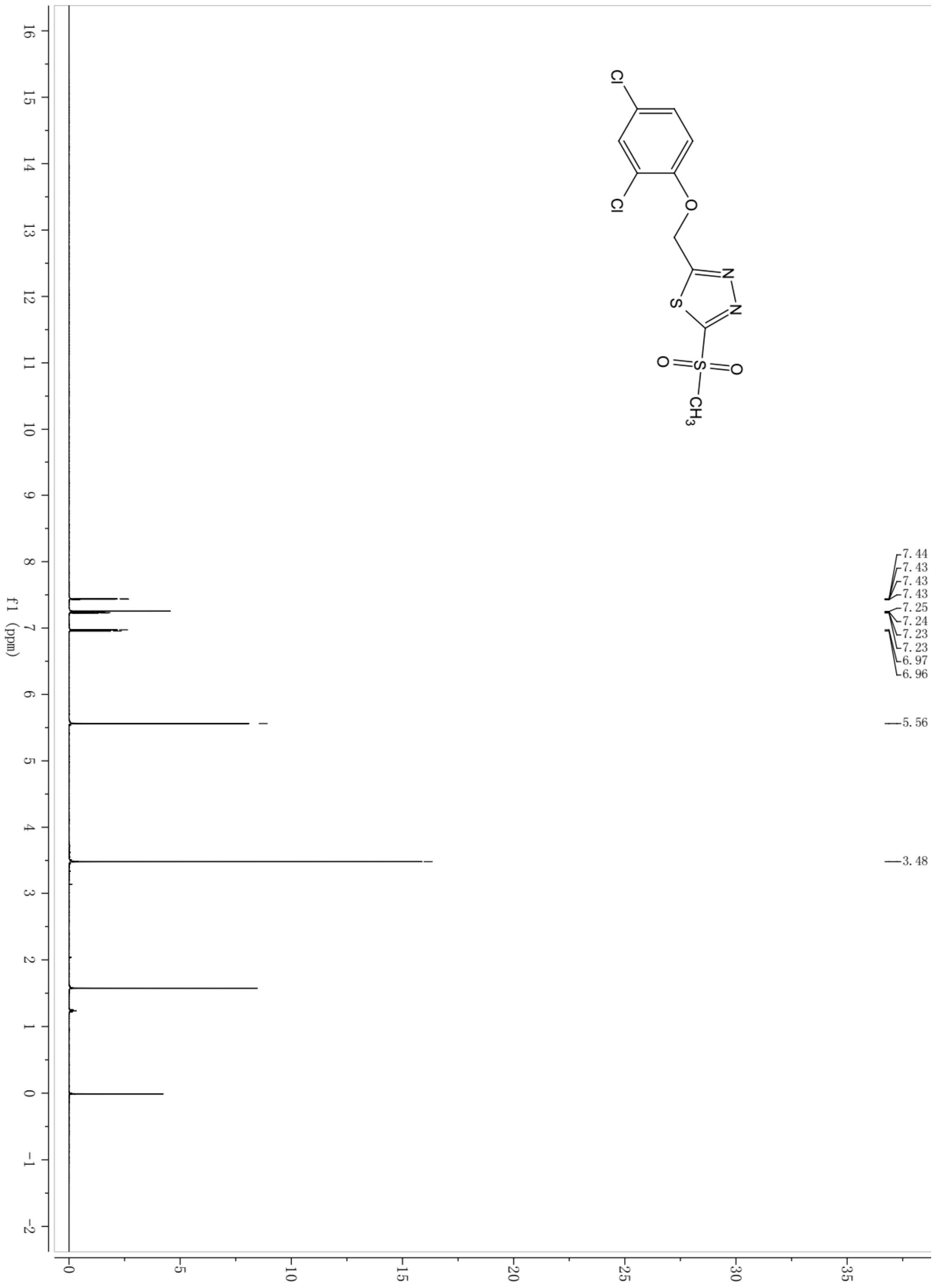

Figure S35. ^{1}H NMR spectrum of compound **5II-4**.

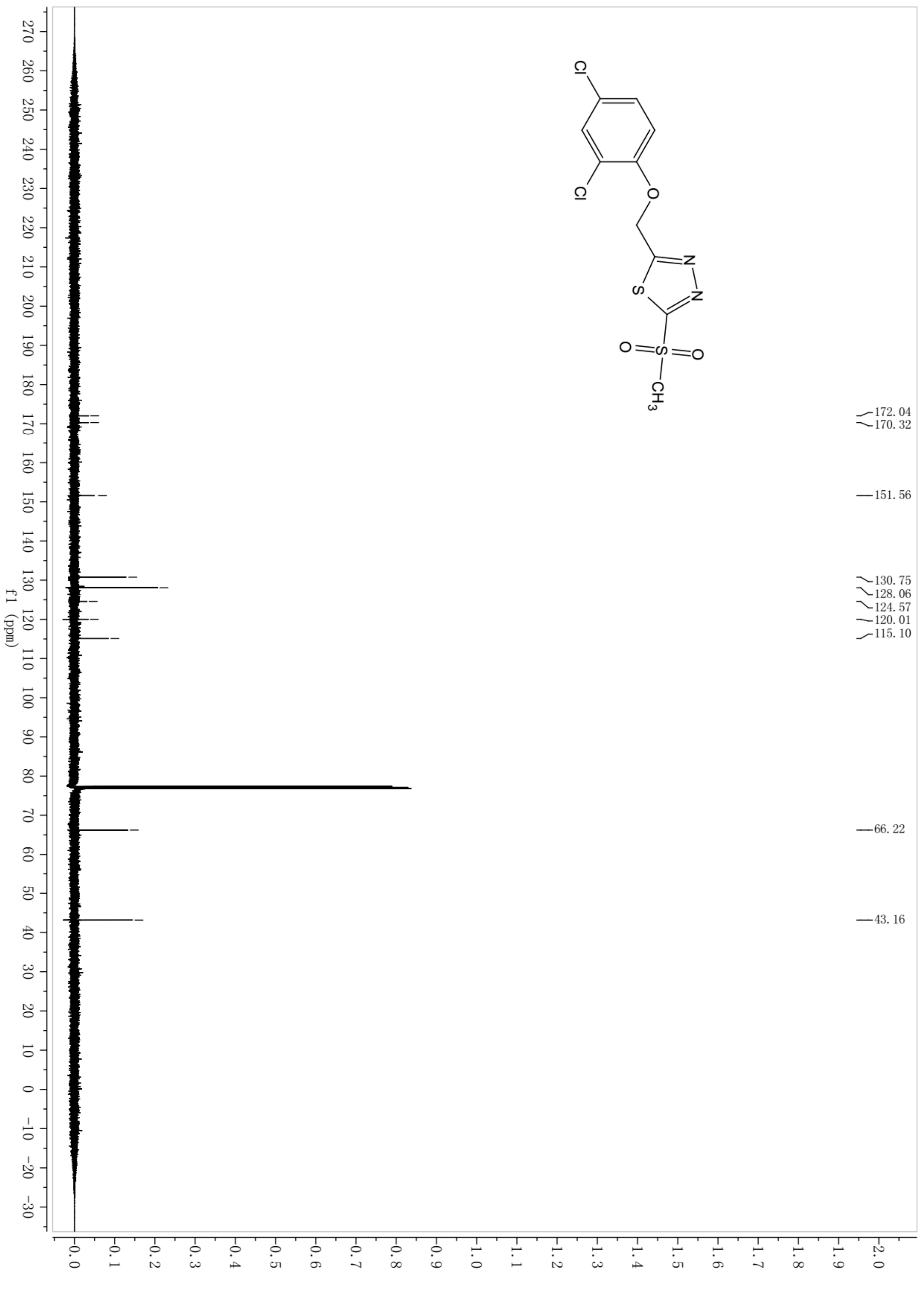

Figure S36. ^{13}C NMR spectrum of compound **5II-4**.

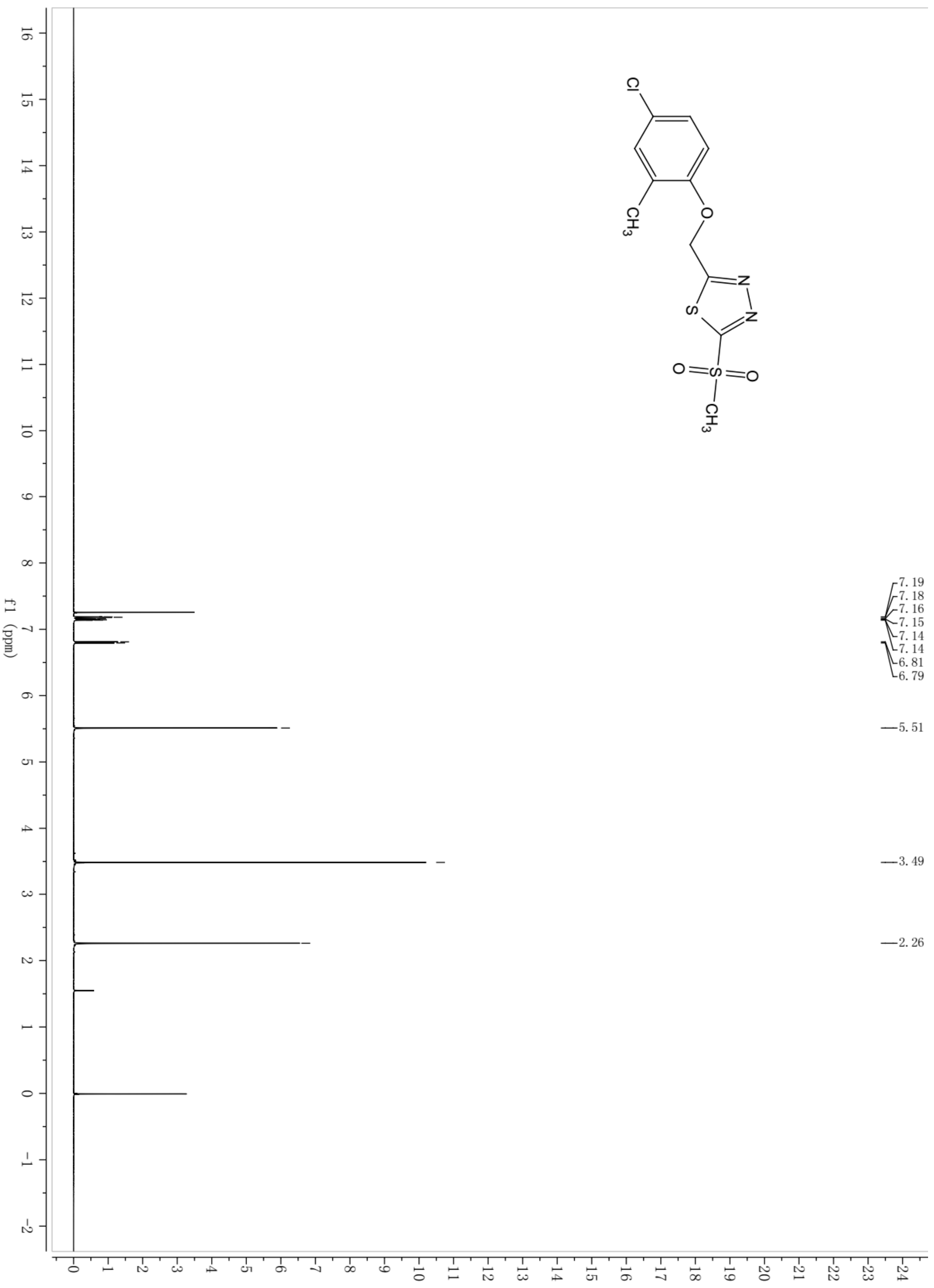

Figure S37. ^{1}H NMR spectrum of compound **5II-5**.

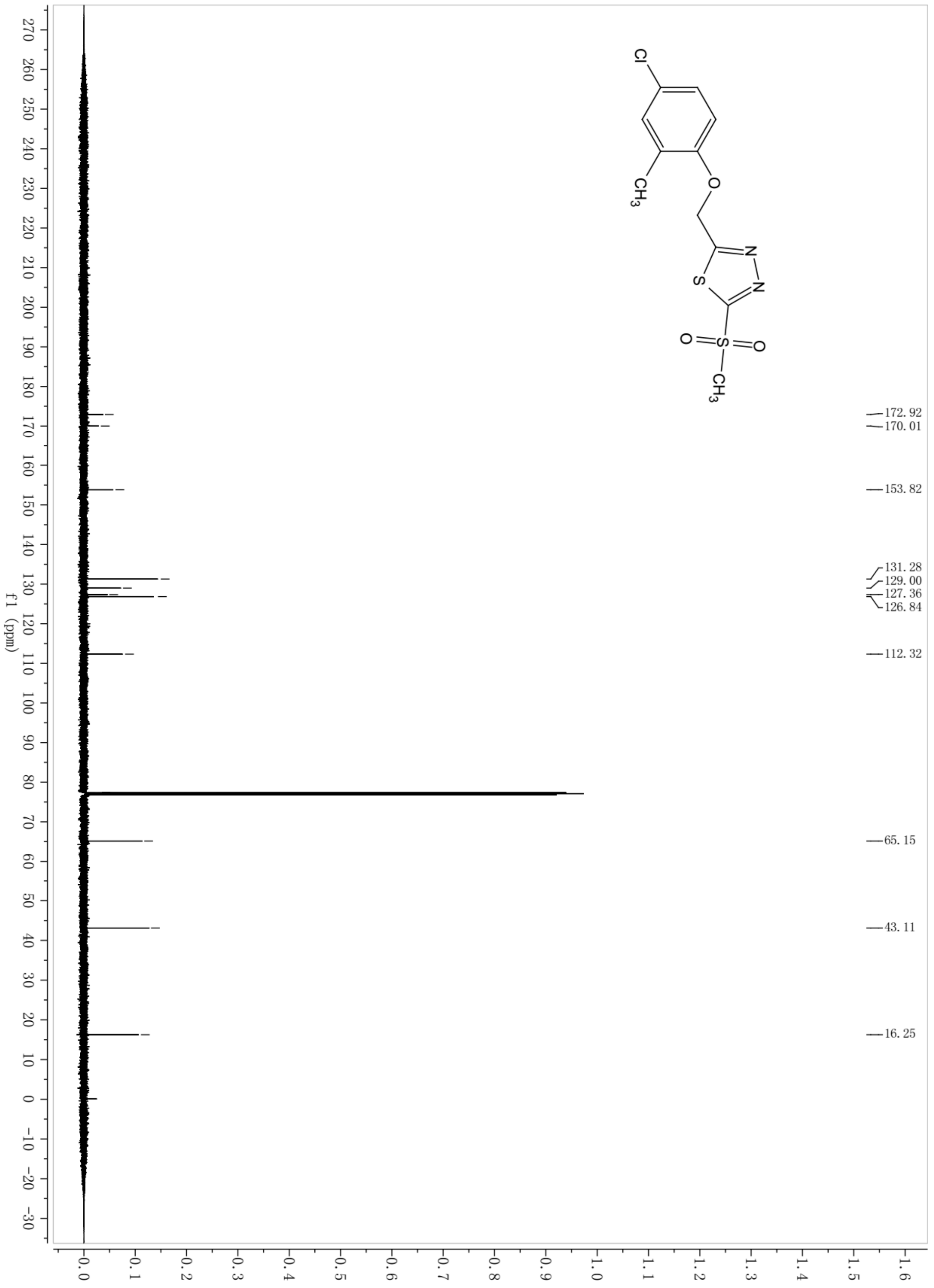

Figure S38. ^{13}C NMR spectrum of compound **5II-5**.

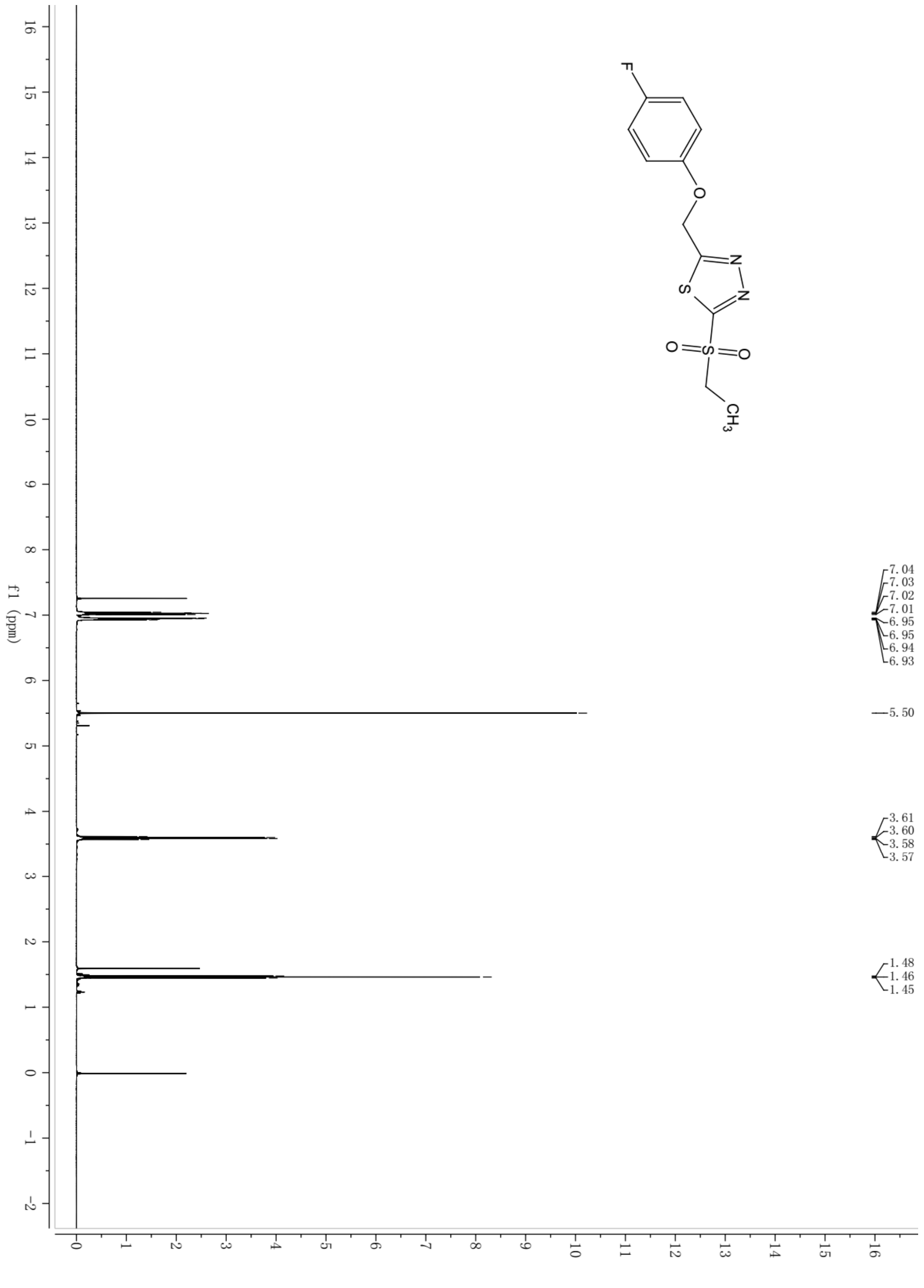

Figure S39. ^{1}H NMR spectrum of compound **5II-6**.

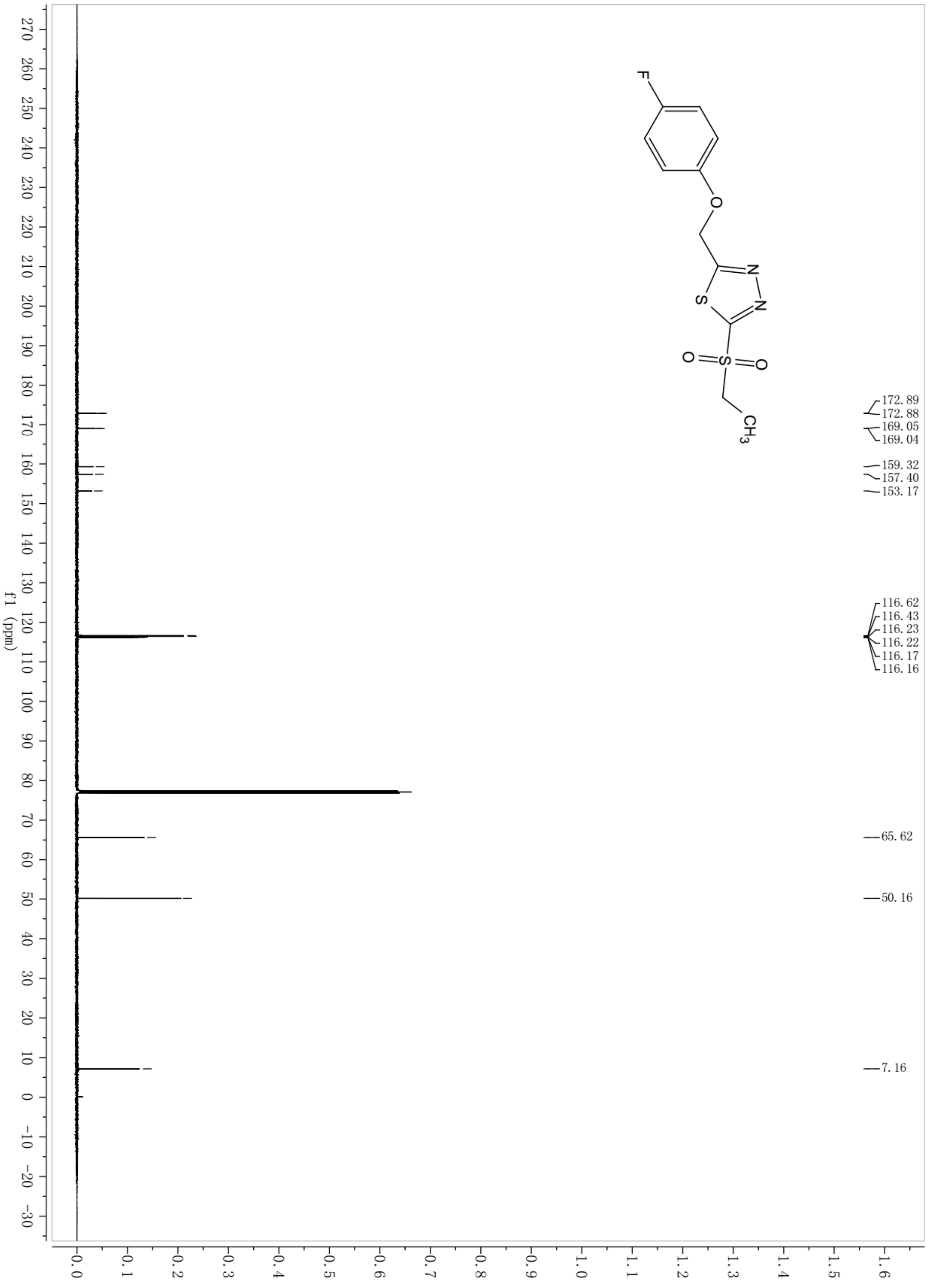

Figure S40. ^{13}C NMR spectrum of compound **5II-6**.

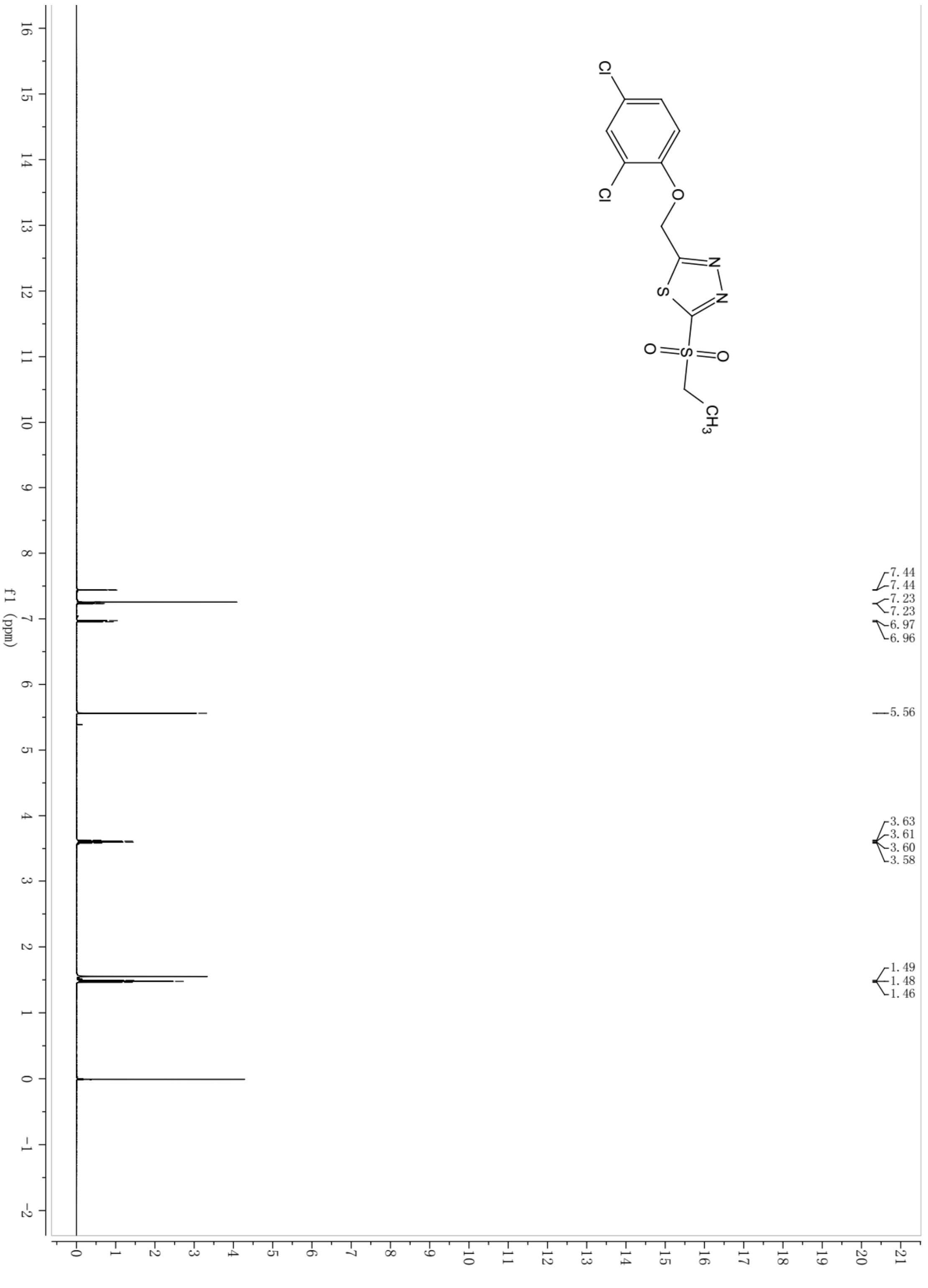

Figure S41. ^{1}H NMR spectrum of compound **5II-7**.

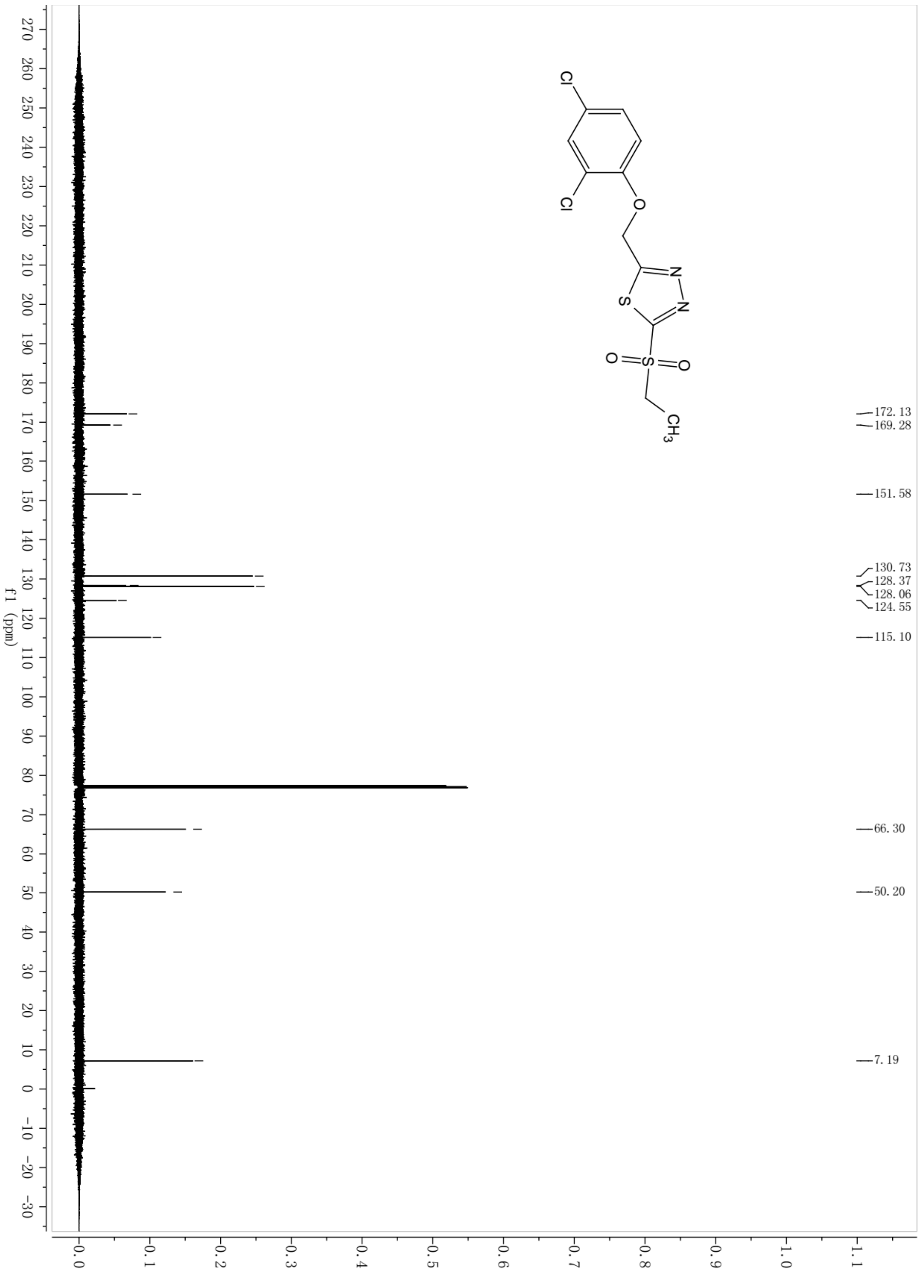

Figure S42. ^{13}C NMR spectrum of compound **5II-7**.

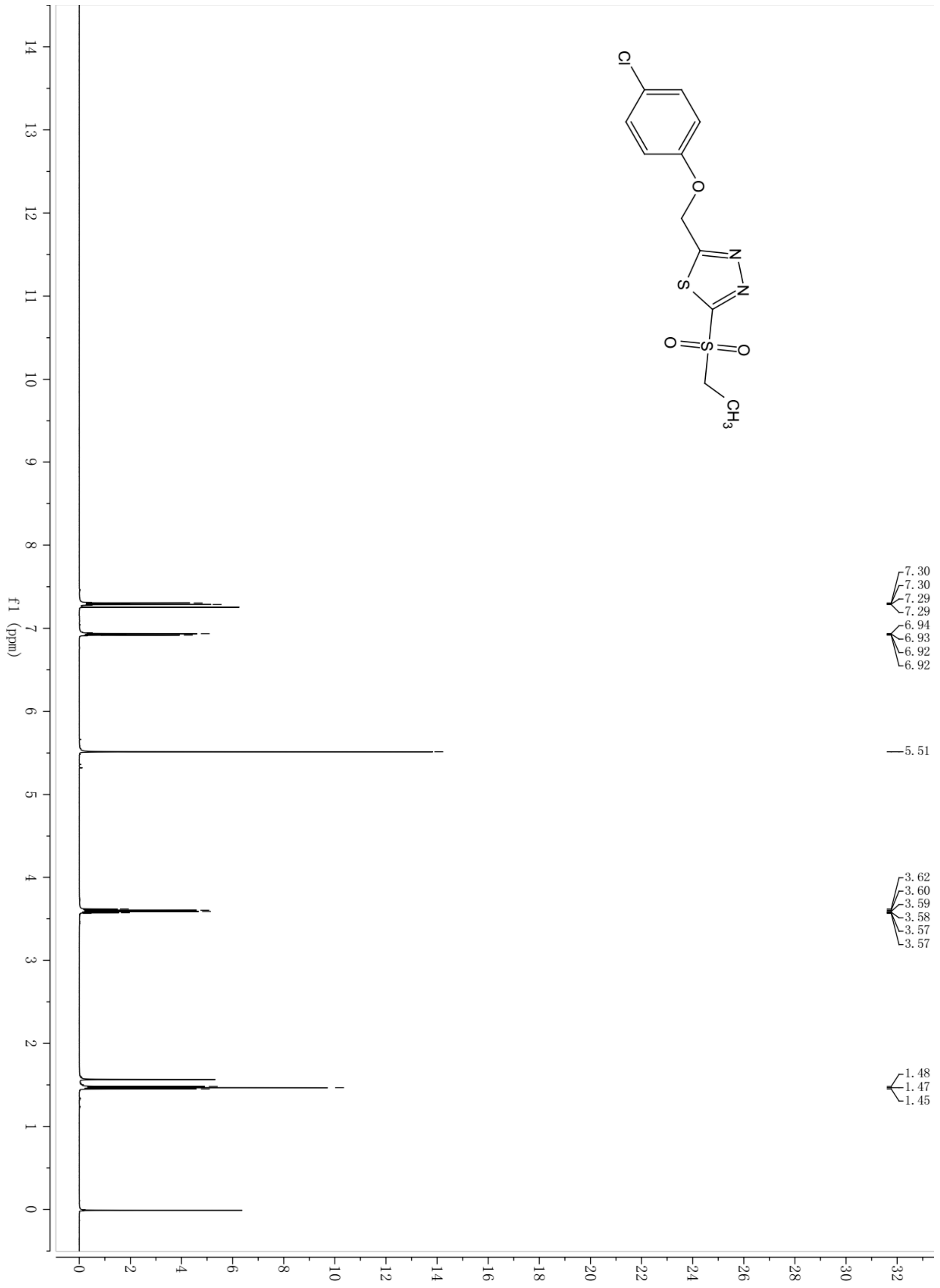

Figure S43. ^{1}H NMR spectrum of compound **5II-7**.

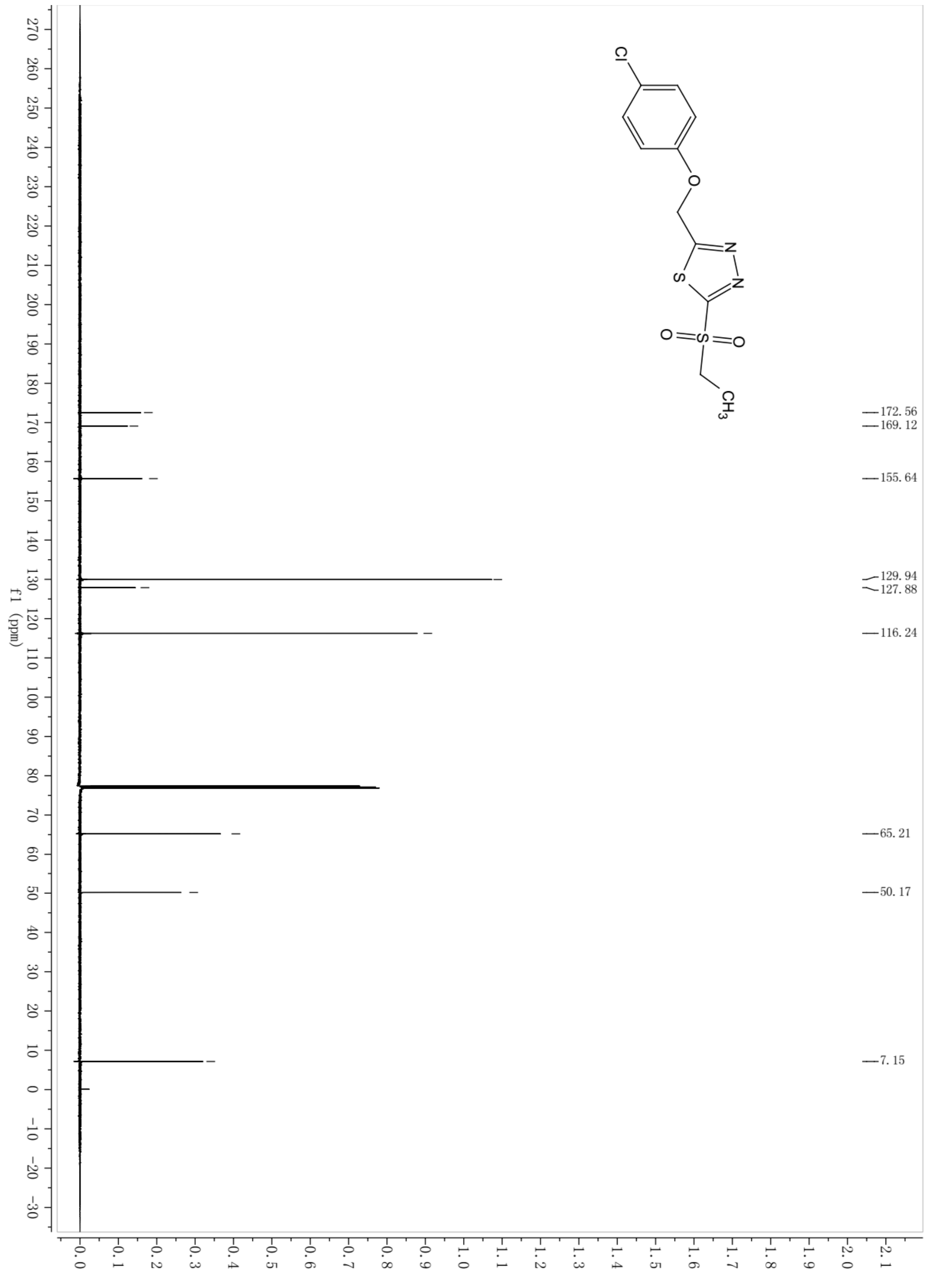

Figure S44. ^{13}C NMR spectrum of compound **5II-7**.

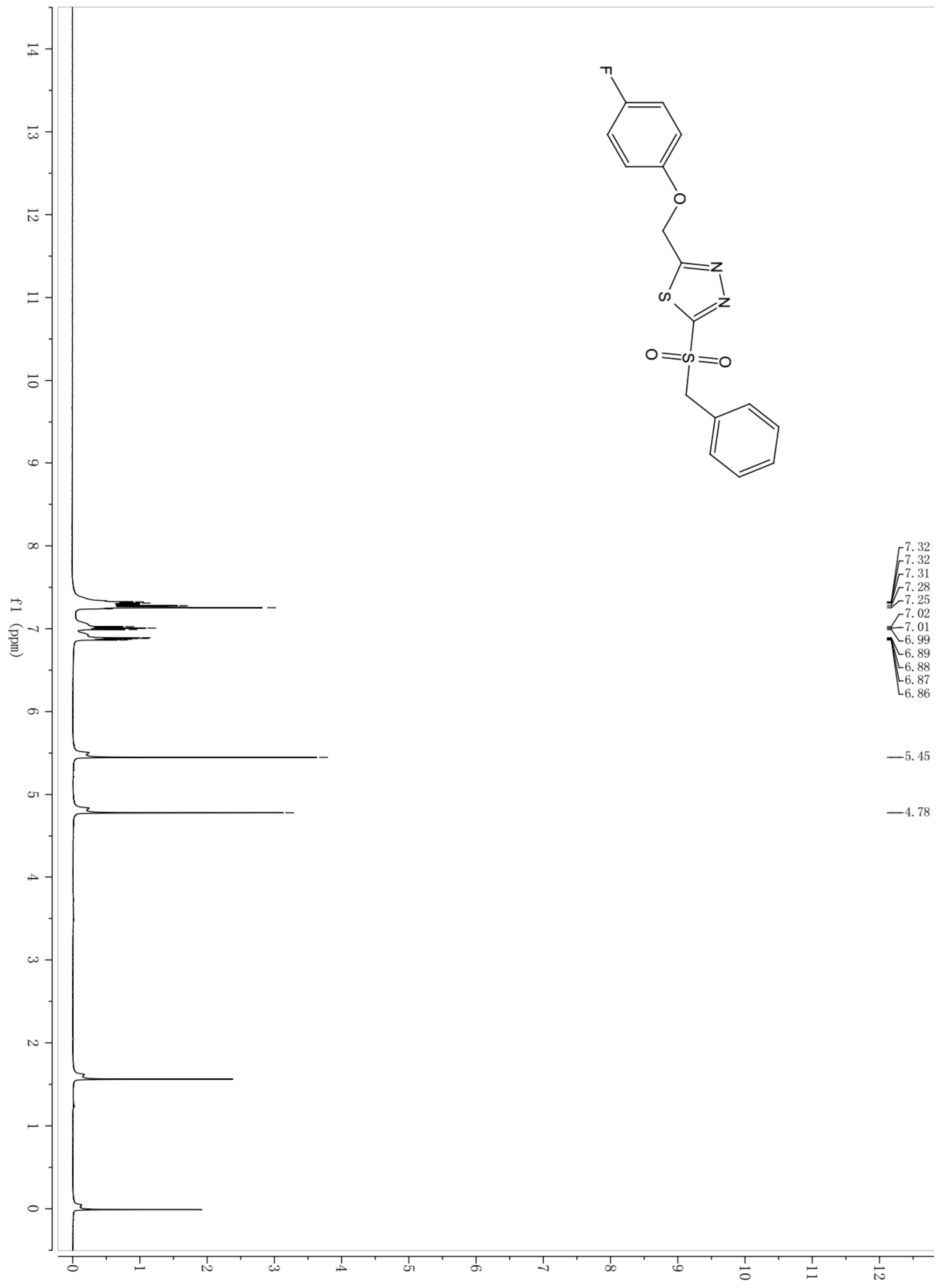

Figure S45. ^{1}H NMR spectrum of compound **5II-8**.

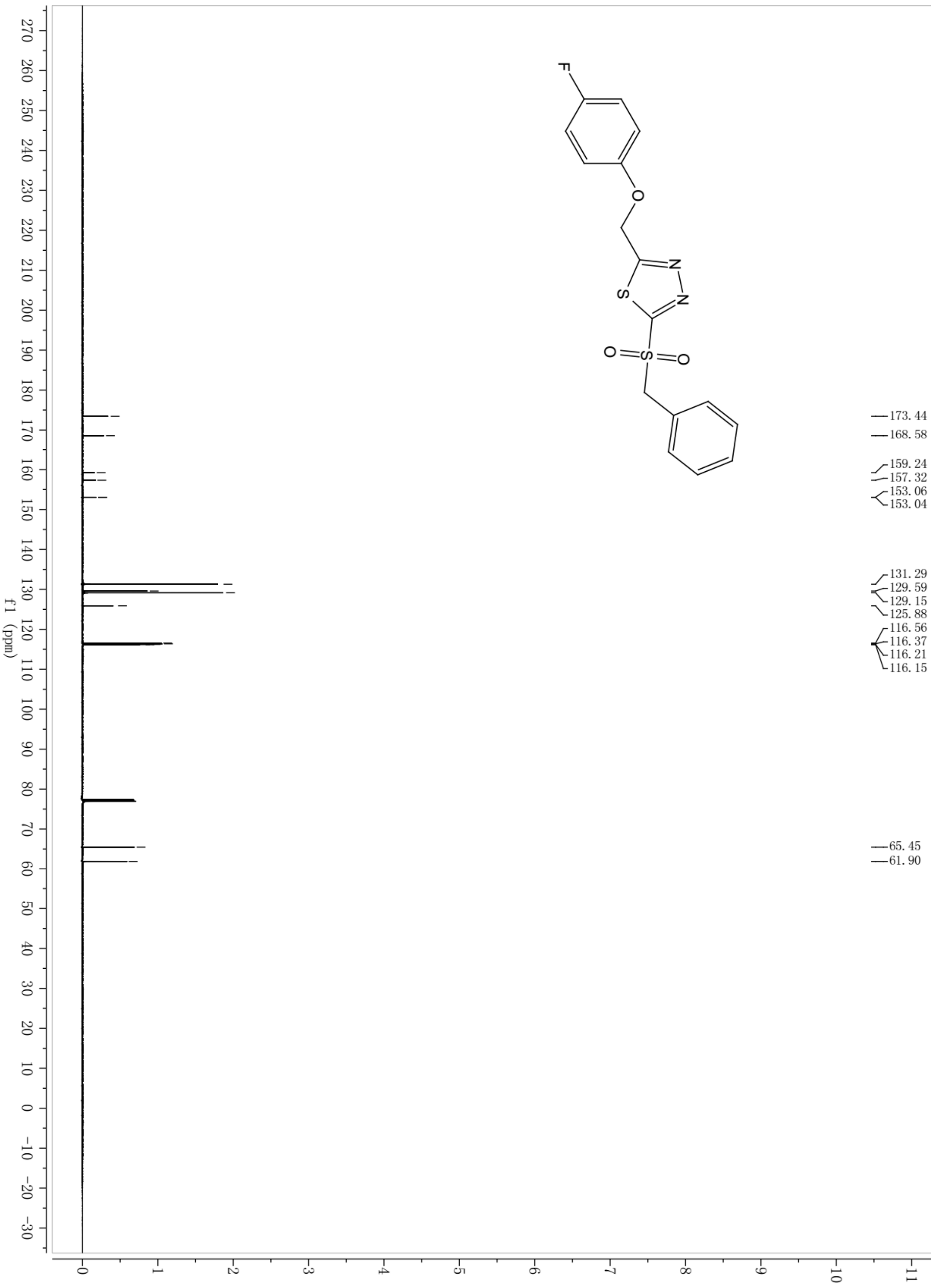

Figure S46. ^{13}C NMR spectrum of compound **5II-8**.

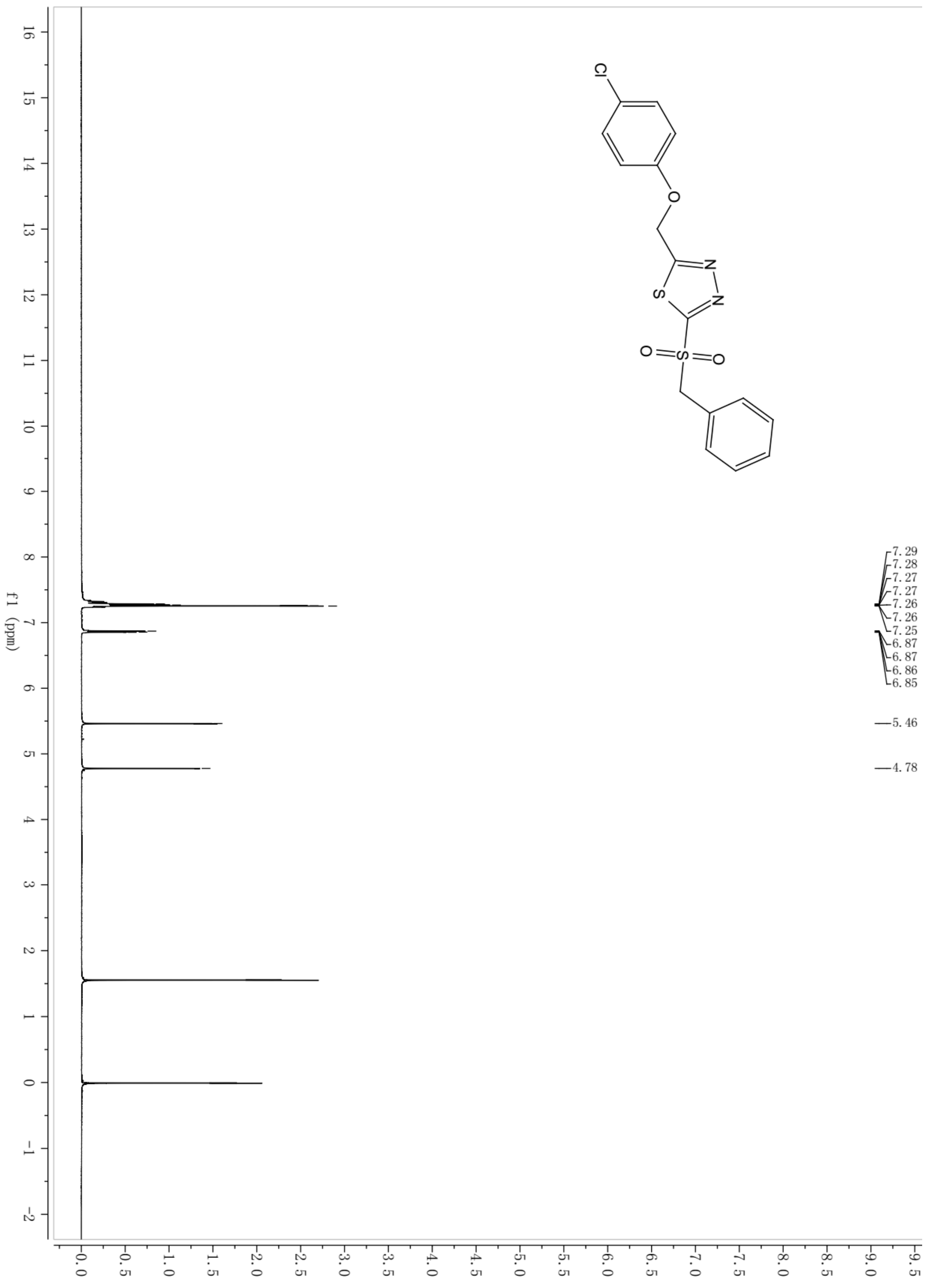

Figure S47. ^{1}H NMR spectrum of compound **5II-9**.

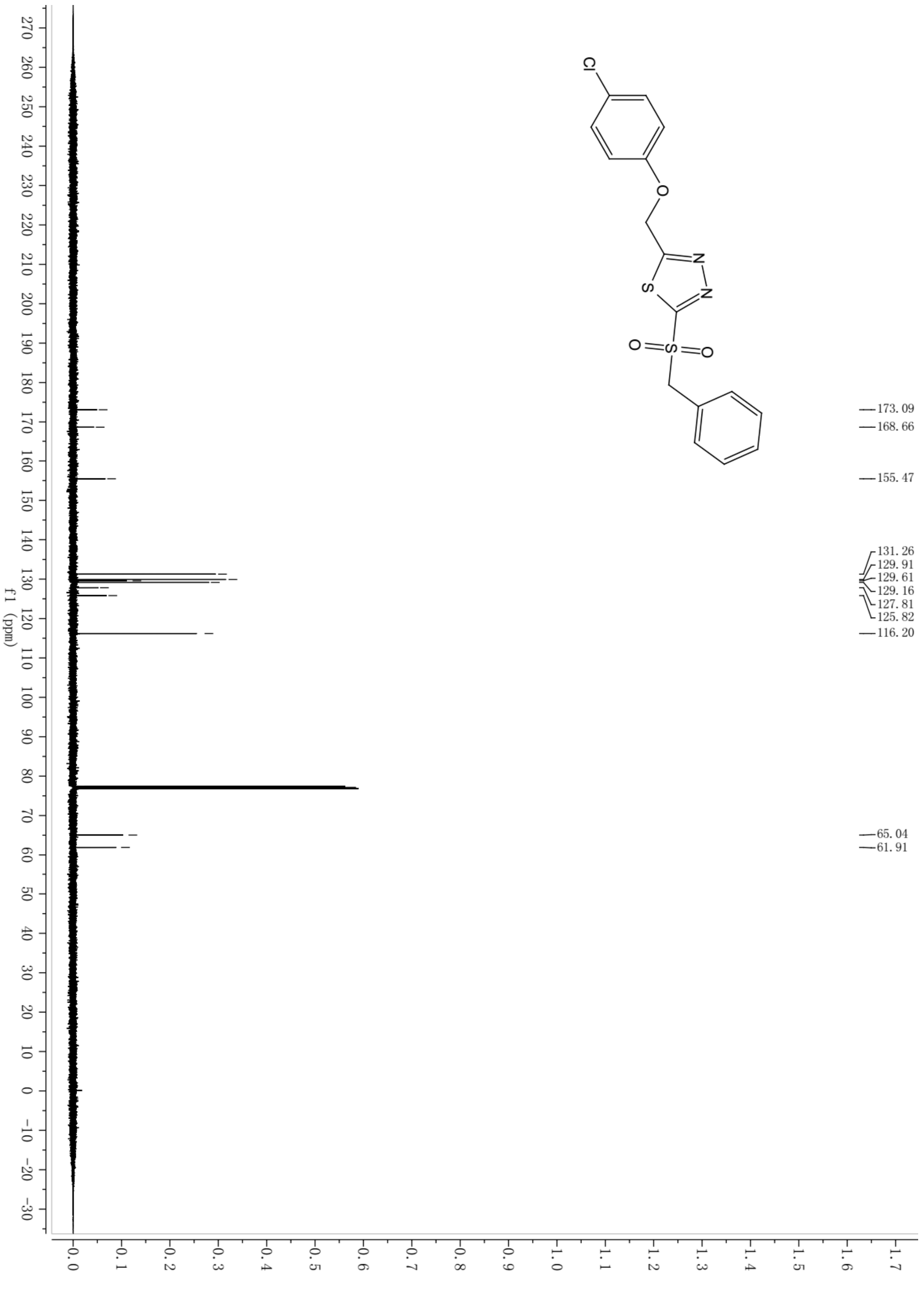

Figure S48. ^{13}C NMR spectrum of compound **5II-9**.

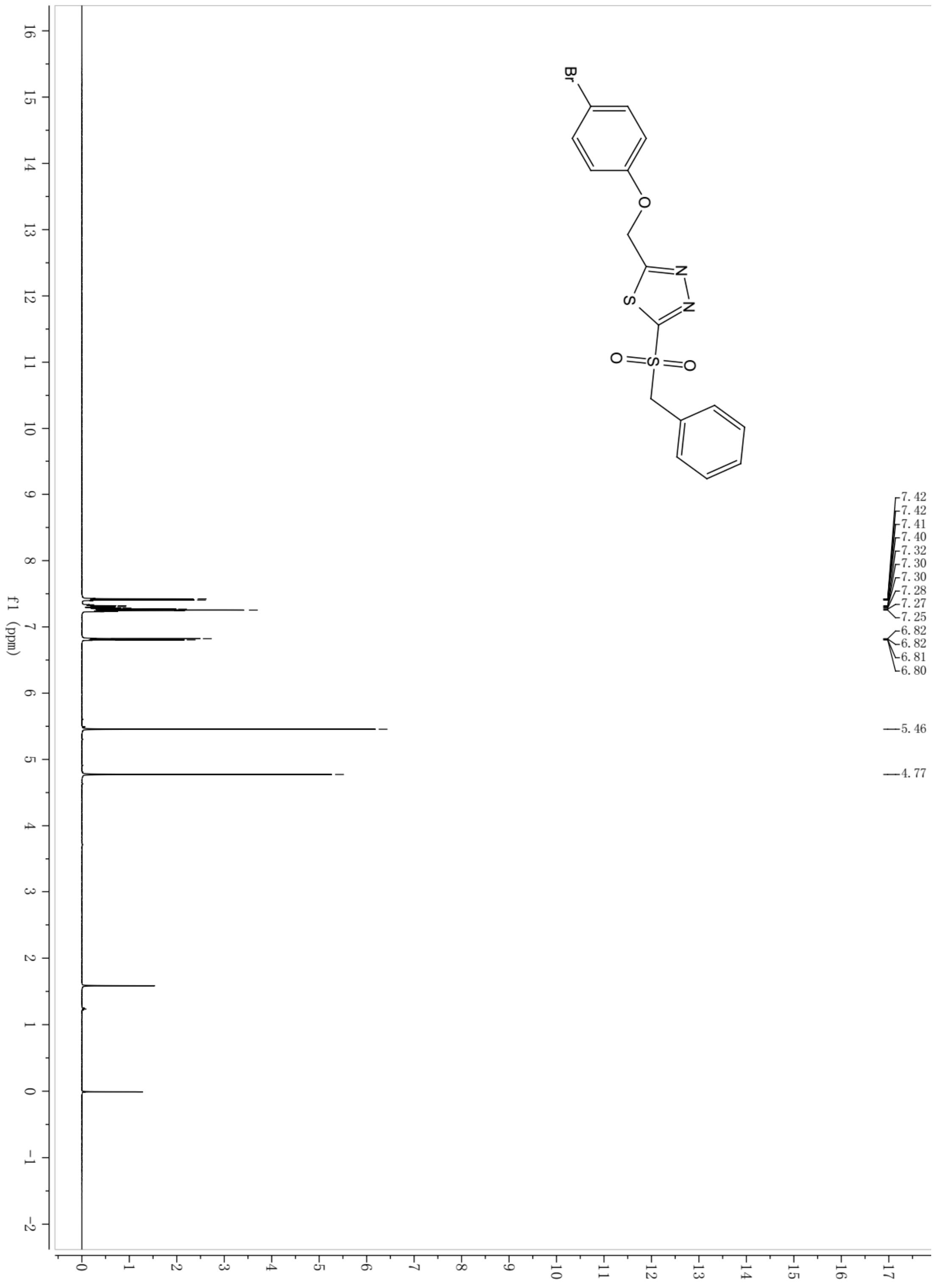

Figure S49. ^{1}H NMR spectrum of compound **5II-10**.

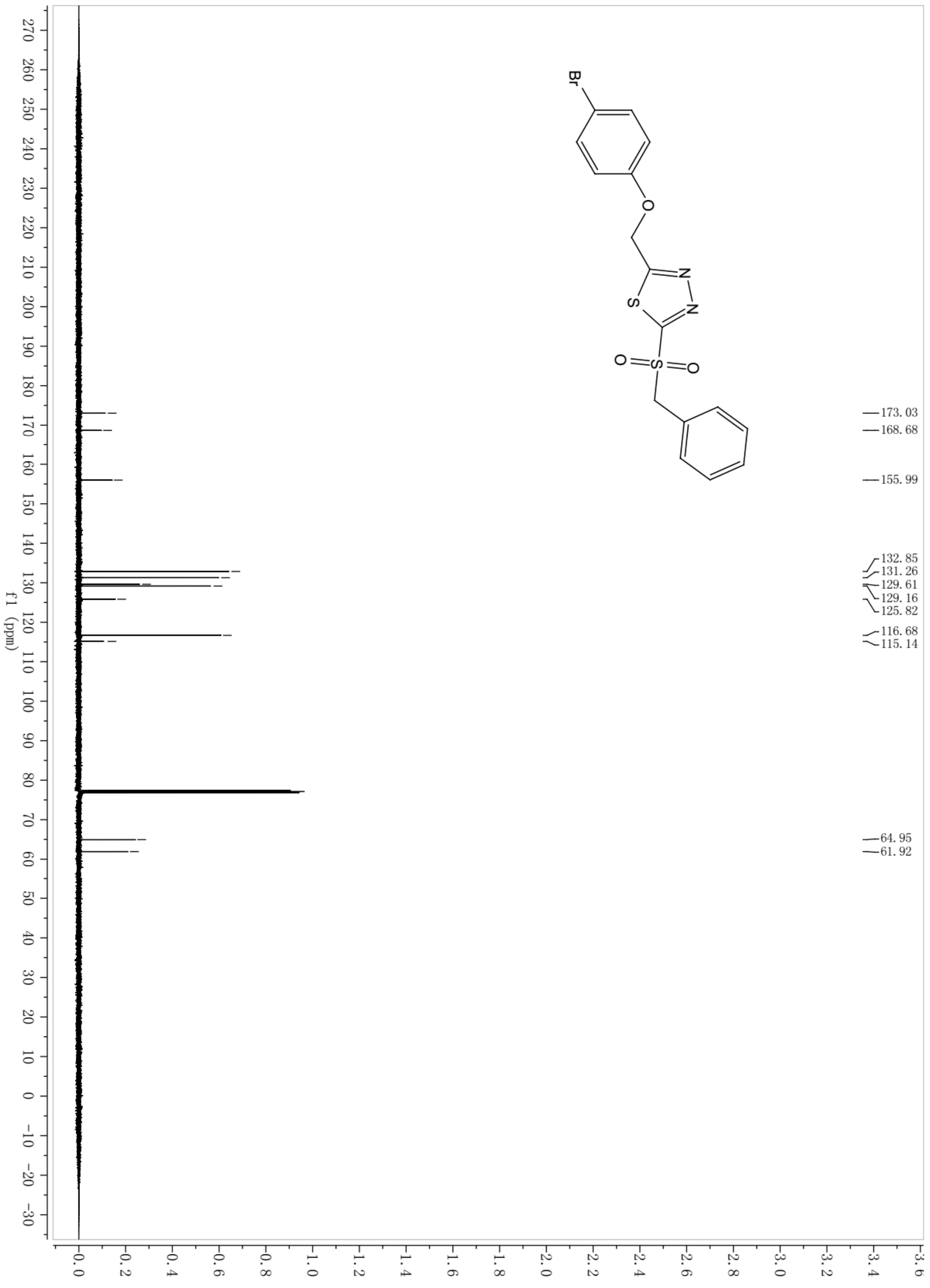

Figure S50. ^{13}C NMR spectrum of compound **5II-10**.

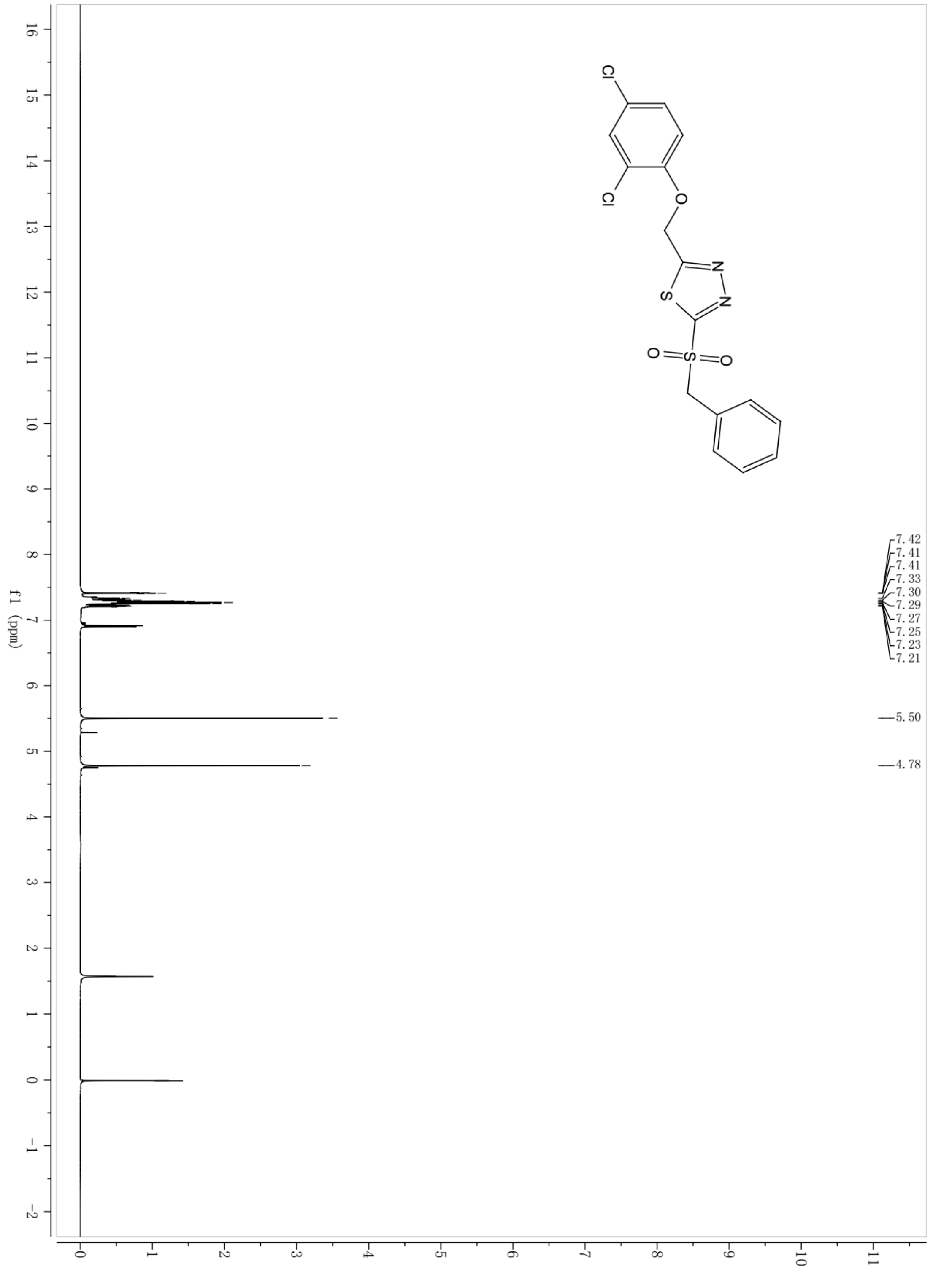

Figure S51. ^{1}H NMR spectrum of compound **5II-11**.

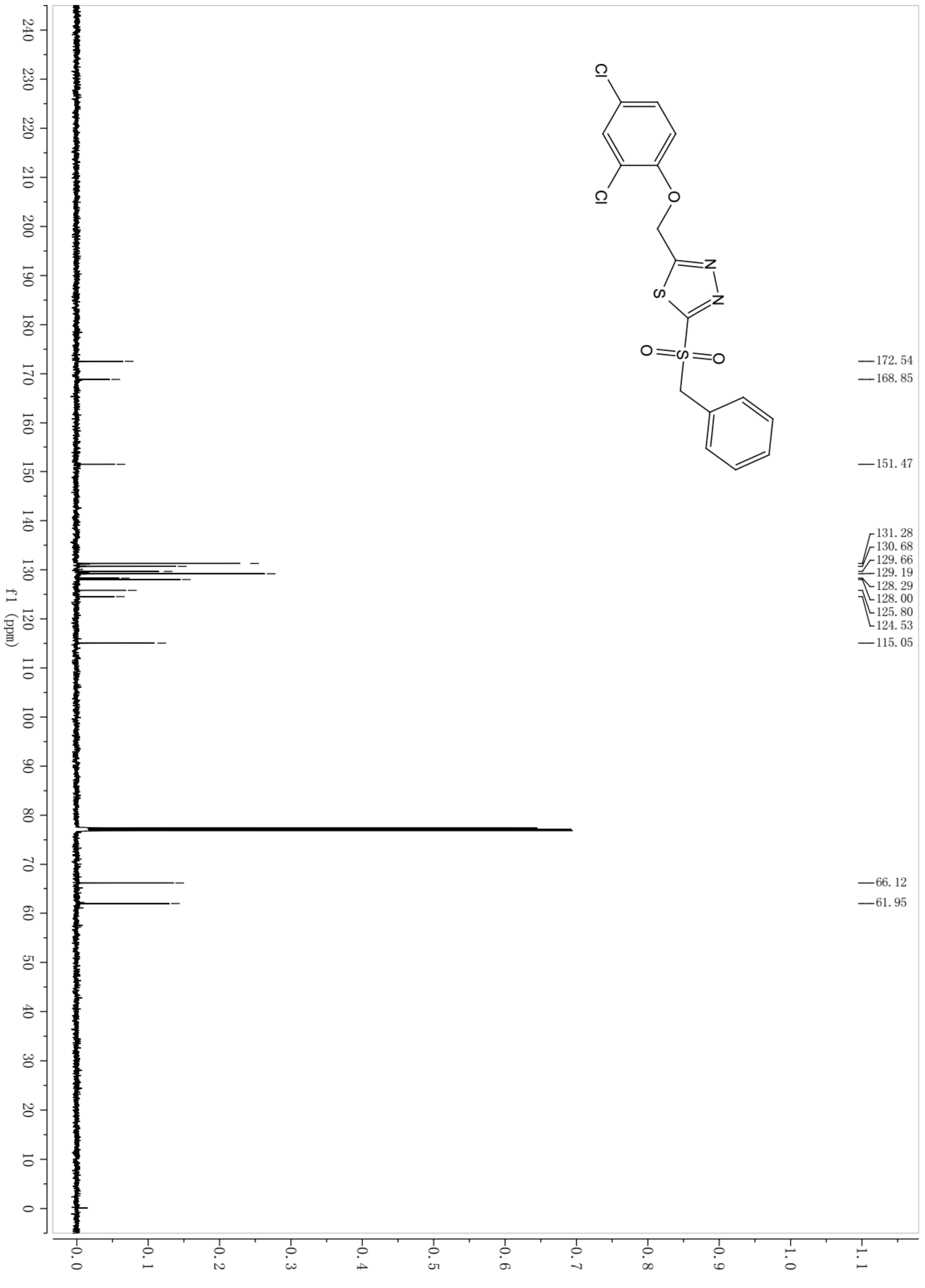

Figure S52. ^{13}C NMR spectrum of compound **5II-11**.

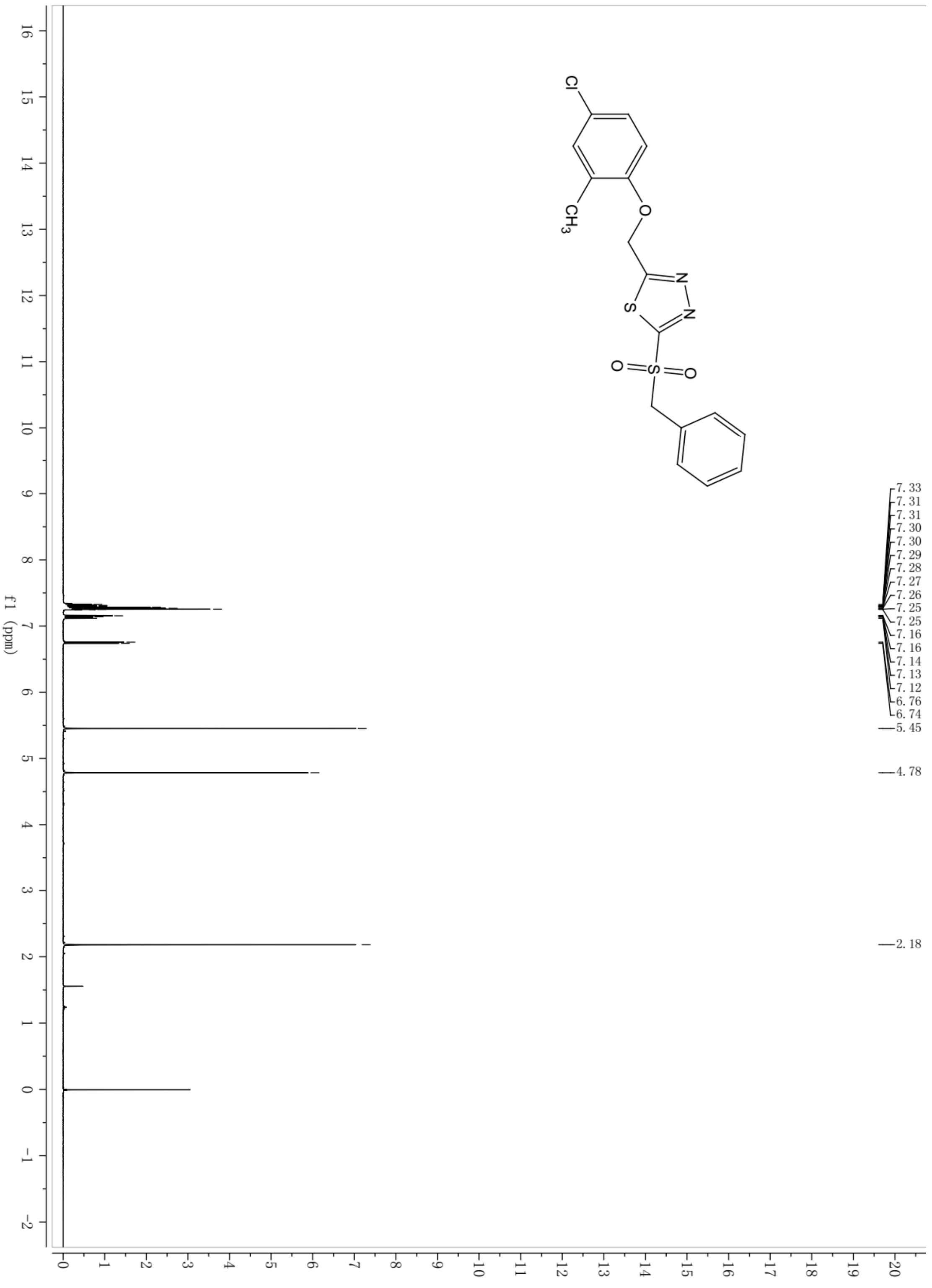

Figure S53. ^{1}H NMR spectrum of compound **5II-12**.

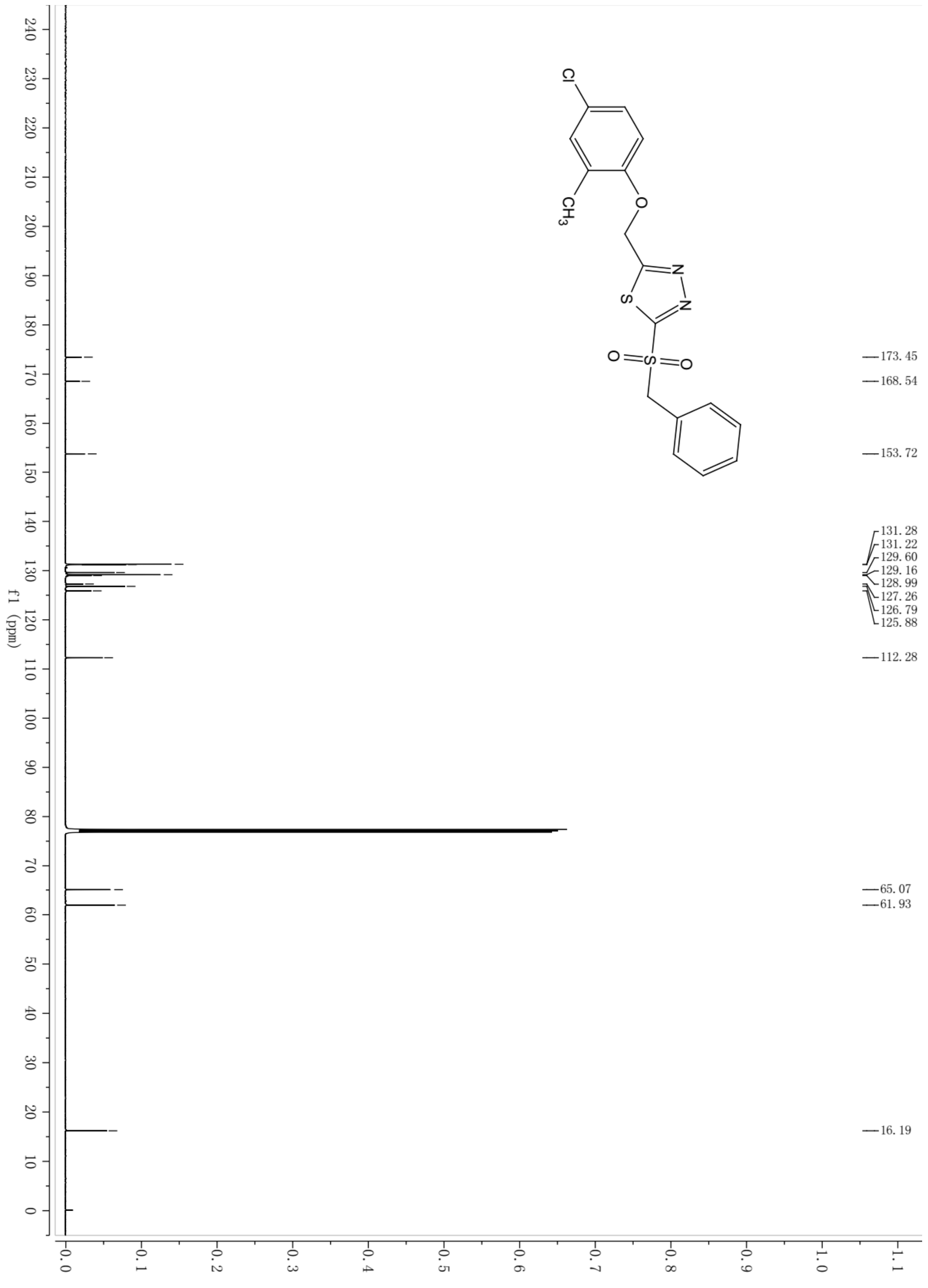

Figure S54. ^{13}C NMR spectrum of compound **5II-12**.
